# Imidazole-Functionalized Thieno[3,2‑*c*]quinolines as Promising Antiproliferative Agents: Design, Synthesis, NCI-60 Screening, and Computational Analysis

**DOI:** 10.1021/acsomega.6c02681

**Published:** 2026-05-07

**Authors:** Gabriele La Monica, Alessia Bono, Federica Alamia, Dennis Tocco, Antonino Lauria, Annamaria Martorana

**Affiliations:** a Dipartimento di Scienze e Tecnologie Biologiche Chimiche e Farmaceutiche “STEBICEF”, 18998 University of Palermo, Viale delle Scienze−Ed. 17, Palermo 90128, Italy; b Fondazione Umberto Veronesi (FUV), via Solferino 19, Milano 20121, Italy

## Abstract

Polycondensed thieno­[3,2-*c*]­quinoline scaffolds represent promising yet underexplored frameworks for anticancer drug discovery. Building on previously reported derivatives of type **7**, a new series of imidazole-functionalized thieno­[3,2-*c*]­quinoline-2-carboxylates (**10a–j**) was rationally designed to optimize physicochemical, drug-like, and safety-related properties and synthesized through a multistep synthetic sequence. Antiproliferative activity was evaluated in vitro by using the NCI-60 human tumor cell line panel. Six derivatives displayed low-to-submicromolar mean GI_50_ values (0.98–5.55 μM), with compound **10c** emerging as the most potent analogue (mean GI_50_ < 1 μM) across multiple aggressive tumor models. Integrated computational analyses, including docking, binding free-energy molecular mechanics with generalized Born and surface area calculations, molecular dynamics simulations, and density functional theory studies, supported enhanced interaction profiles and electronic adaptability of the optimized core relative to the parent series. Overall, the imidazole-functionalized thienoquinoline hybrids exhibit broad antiproliferative activity and improved predicted developability, identifying this chemotype as a valuable platform for further anticancer optimization and structure–activity investigations.

## Graphical abstract

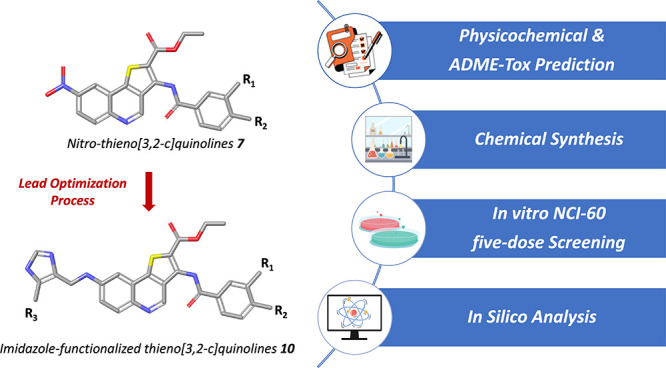

## 1 Introduction

Polycyclic quinoline derivatives, in which the heteroaromatic core is fused with additional aromatic or heteroaromatic rings, have emerged as valuable scaffolds in anticancer drug discovery.^1–3^ Their rigid and π-conjugated frameworks favor productive interactions with flat or hydrophobic binding sites commonly found in oncogenic targets, while also influencing key pharmacokinetic properties such as lipophilicity and cellular permeability.^4–7^ Several quinoline-fused systems have progressed to clinical use or advanced development stages, including DNA-intercalating agents, topoisomerase inhibitors, kinase inhibitors, and immunomodulatory compounds, underscoring the pharmacological relevance of this heteroaromatic architecture.^4,5^

Within this context, thienoquinoline derivatives, featuring a thiophene ring fused to the quinoline core, represent a comparatively underexplored chemotype. Advances in synthetic methodologies have enabled access to structurally diverse thienoquinoline frameworks, which have attracted attention not only for medicinal chemistry applications but also for their distinctive photochemical properties.^8–11^

From a medicinal chemistry perspective, thieno­[3,2-*c*]­quinoline systems are of particular interest because their fusion geometry resembles that of well-established anticancer imidazo­[4,5-*c*]­quinolines, suggesting potentially analogous activities. Moreover, replacement of a nitrogen-containing heterocycle with a sulfur-based ring may modulate chemical and metabolic stability relative to the corresponding aza-analogues.^8–11^ Consistently, several thieno­[3,2-*c*]­quinoline derivatives have been reported as modulators of cancer-relevant targets (Figure 1), including PI3K inhibitors (compounds **1** and **2**),^12,13^ dual CK2/PIM kinase inhibitors (compound **3**),^14^ Mer kinase inhibitors (compound **4**),^15^ and PARP1 inhibitors (compounds **5** and **6**).^16,17^ These studies have been primarily conducted at a preclinical level, mainly through in vitro investigations, providing initial evidence of the significant potential of the thienoquinoline framework in the anticancer field.

**Figure 1:**
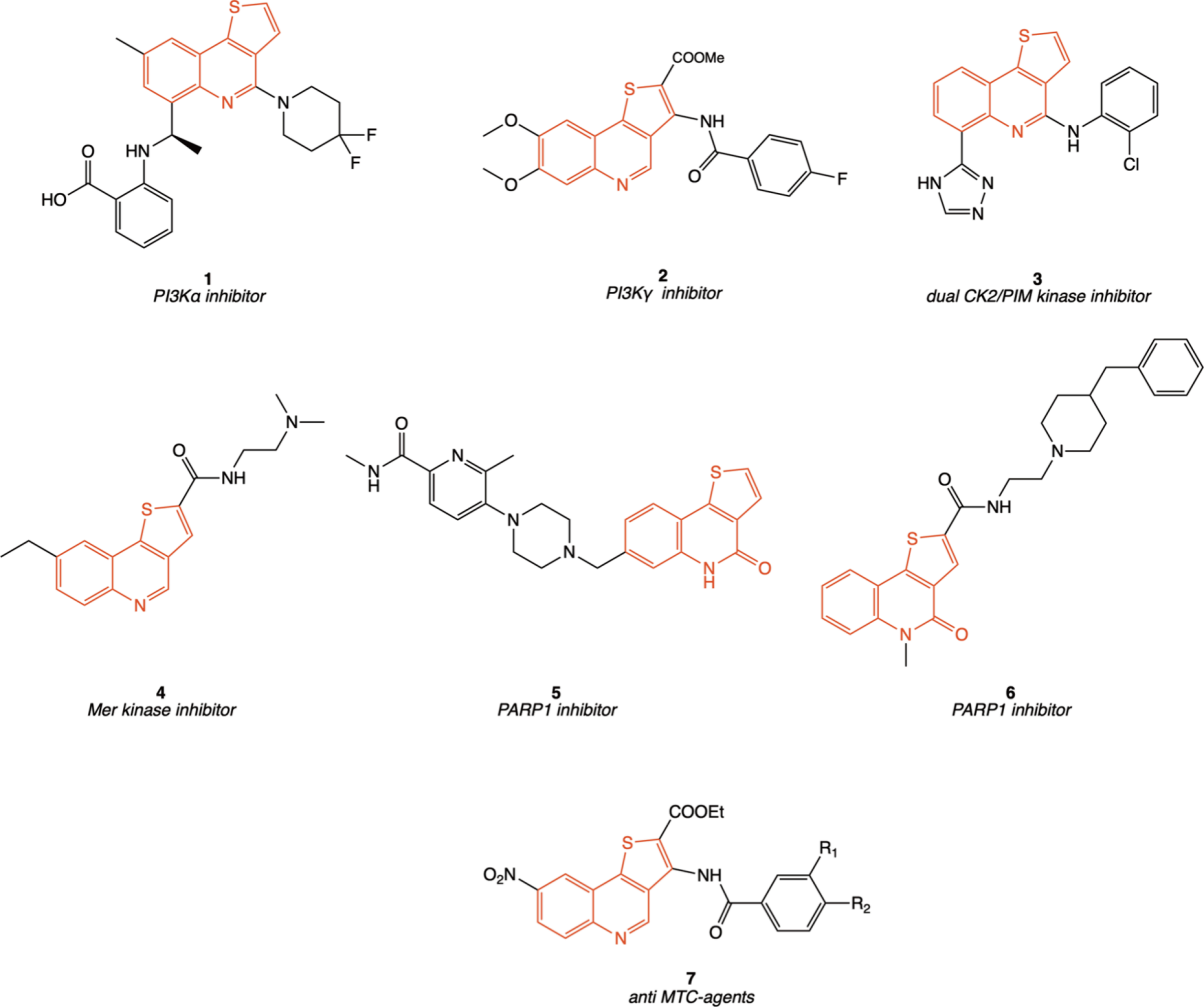
Representative examples of biologically active thieno­[3,2-*c*]­quinoline derivatives reported in the literature: compounds **1** and **2,** PI3K inhibitors; compound **3**, dual CK2/PIM kinase inhibitor; compound **4**, targeting Mer kinase; compounds **5** and **6,** PARP1 inhibitors; compounds of type **7** with activity against thyroid cancer cells.

Despite these promising precedents, systematic scaffold optimization of thieno­[3,2-*c*]­quinolines toward improved developability and broad phenotypic antiproliferative profiling remains limited. In particular, the impact of targeted functional-group modulation on the physicochemical balance, predicted safety parameters, and multitarget anticancer activity has not been comprehensively investigated.

In our previous work, we reported a series of 8-nitroquinoline derivatives (**7**), rationally designed through structure-based in silico approaches and evaluated in cellular models of medullary thyroid carcinoma (MTC), where they displayed low-to-mid micromolar antiproliferative activity.^18^ These findings validated the thienoquinoline chemotype in a biologically relevant cancer model and provided a foundation for further optimization.

Guided by these results, and inspired by our earlier lead-optimization studies on nitro-benzo­[*b*]­thiophene and nitro-benzo­[*b*]­furan derivatives (**8** and **9**), in which imidazole incorporation improved aqueous solubility and antiproliferative activity,^19,20^ we designed a new library of imidazole-functionalized thieno­[3,2-*c*]­quinoline hybrids (type **10**, Figure 2) starting from the previously developed parent thienoquinoline derivatives (type **7**, Figure 2).

**Figure 2:**
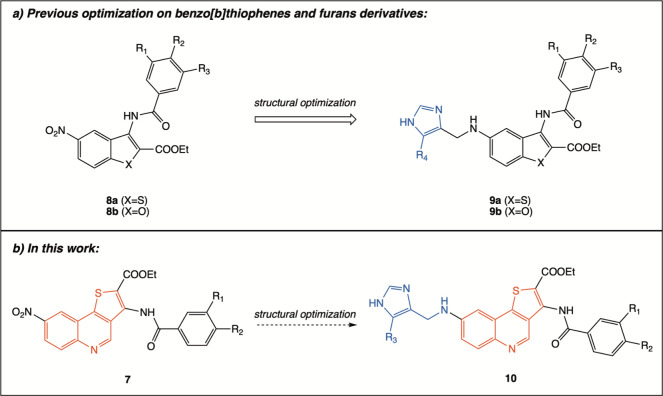
Lead-optimization strategy adopted in this study. Starting from previously reported 8-nitroquinoline derivatives of type **7** (parent compounds), introduction of an imidazole fragment was pursued following the same conceptual approach previously applied to benzofuran and benzothiophene series (a, compounds **8a,b** → **9a,b**), leading to the design of the new library of imidazole-functionalized thienoquinoline hybrid derivatives of (b) type **10**.

The introduction of the imidazole fragment was conceived according to a molecular hybridization strategy, which combines two pharmacologically relevant frameworks within a single framework. The thieno­[3,2-*c*]­quinoline core represents a rigid π-conjugated system with demonstrated antiproliferative potential, whereas the imidazole moiety is a well-recognized privileged structure in anticancer drug discovery, frequently associated with enhanced polarity, hydrogen-bonding capability, and multitarget engagement.^21–24^ The resulting hybrid architecture was therefore expected to modulate the electronic distribution and interaction capacity while preserving the structural features responsible for the antiproliferative profile of the parent scaffold.

Rather than focusing on a single predefined molecular target, the new hybrid compounds were conceived as phenotype-driven anticancer agents, potentially characterized by a multitarget interaction profile consistent with the structural features of the scaffold and the complexity of cancer signaling networks. To evaluate their translational relevance, the rationally designed library was synthesized and assessed using the NCI-60 human tumor cell line panel, enabling comparison across diverse cancer types. Complementary in silico physicochemical profiling and computational studies were performed to rationalize the effects of imidazole functionalization on developability and to explore plausible target interactions underlying the observed cellular activity.

## 2 Results and Discussion

### 2.1 Physicochemical and ADME-Tox Property Prediction

To rationalize the structural evolution from nitro-substituted thienoquinoline derivatives to imidazole-containing analogues and to support the improved developability of the optimized series, a preliminary in silico analysis of physicochemical and ADME-Tox-related properties was performed. The chemical structures and substitution patterns of the previously reported derivatives **7a**–**e** and the newly designed imidazole-thienoquinoline compounds **10a–j** are shown in Figure 3.

**Figure 3:**
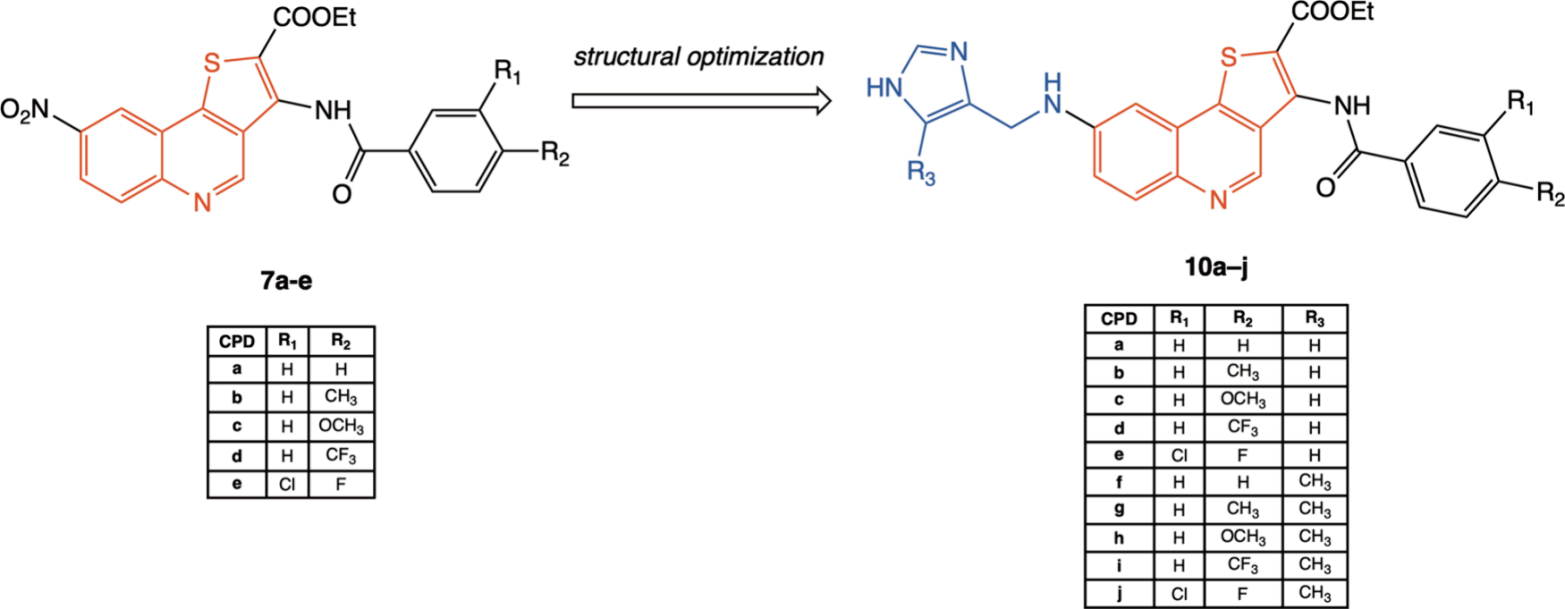
Chemical structure of lead thieno­[3,2-*c*]­quinoline-2-carboxylates **7a**–**e** and optimized imidazole-thienoquinoline hybrid compounds **10a**–**j**.

Prior to profiling, all ligands were prepared under standardized conditions to generate the most representative protonation and tautomeric states at physiological pH using LigPrep (Maestro, Schrödinger LLC, Release 2017-1).^25^ Particular attention was devoted to the imidazole-containing derivatives, whose ionization state may significantly influence the polarity and absorption-related descriptors. Under physiological conditions, the imidazole fragment was predominantly protonated, contributing to an increased molecular polarity.

Physicochemical and ADME parameters were predicted using QikProp^26^ and SwissADME,^27^ and the most relevant descriptors are summarized in Tables 1 and 2, while complete data sets are reported in the Supporting Information (Tables S1 and S2).

**Table 1:** Key Structural, Physicochemical, and ADME-Related Properties Predicted In Silico for the Nitro-Substituted Thienoquinoline Derivatives (**7a**–**e**) and the Imidazole-Thienoquinoline Hybrids (**10a**–**j**)­^a^

CPD	MW^#^	logP_o/w_^#^	DM^#^	SASA^#^	HBD^#^	HBA^#^	RB^#^	rtvFG^#^	QPP-Caco^#^	QPP-MDCK^#^	HOA%^#^	P-gp substrate
**7a**	421.43	5.38	0.37	686.53	0	5.5	5	0	89.92	51.34	82.65	no
**7b**	435.45	5.69	0.49	715.42	0	5.5	5	0	90.11	51.41	84.44	no
**7c**	451.45	5.39	1.29	722.56	0	6.25	6	0	90.54	51.72	83.01	no
**7d**	489.43	6.40	3.43	786.71	0	5.5	5	0	90.61	318.76	78.38	no
**7e**	473.86	6.18	3.78	715.18	0	5.5	5	0	91.66	205.00	86.88	no
**10a**	471.53	4.78	10.56	772.49	2	7.5	7	0	177.51	96.02	90.90	no
**10b**	485.56	5.09	10.40	813.75	2	7.5	7	0	162.94	97.51	92.18	no
**10c**	501.56	4.79	13.20	830.66	2	8.25	8	0	170.05	101.82	78.89	no
**10d**	539.53	5.8	11.49	821.57	2	7.5	7	0	178.56	419.45	70.62	no
**10e**	523.97	5.57	12.83	799.40	2	7.5	7	0	177.92	372.57	81.86	no
**10f**	485.56	5.09	14.53	823.17	2	7	7	0	205.58	125.21	96.15	no
**10g**	499.59	5.40	11.44	816.95	2	7	7	0	212.68	116.24	96.90	no
**10h**	515.59	5.10	14.77	860.32	2	7.75	8	0	206.80	126.20	83.79	no
**10i**	553.56	6.11	12.30	858.44	2	7	7	0	200.17	527.45	75.00	no
**10j**	537.99	5.88	14.69	849.18	2	7	7	0	218.88	526.36	74.76	no

a [#] Abbreviations. RB: number of rotatable bonds (optimal values in the range 0–15). rtvFG: number of reactive functional groups. MW: molecular weight. DM: dipole moment. SASA: total solvent-accessible surface area (optimal values in the range 300–1000). HBD: hydrogen bond donors. HBA: hydrogen bond acceptors. Log *P*_o/w_: partition coefficient octanol/water. QPP-Caco-2: predicted apparent Caco-2 cell permeability in nm/sec (<25 poor, >500 great). QPP-MDCK: predicted apparent MDCK cell permeability in nm/sec (<25 poor, >500 great). HOA%: predicted human oral absorption on 0 to 100% scale. P-gp: glycoprotein P.

**Table 2:** Drug-Likeness Rules and Alerts Predicted In Silico for the Nitro-Substituted Thienoquinoline Derivatives (**7a**–**e**) and the Imidazole-Containing Analogues (**10a**–**j**)­^a^

CPD	QP Stars^#^	LRoF	VV	PAINS	Brenk alerts
**7a**	2	0	1	0	2
**7b**	2	0	1	0	2
**7c**	1	0	1	0	2
**7d**	2	0	1	0	2
**7e**	2	0	1	0	2
**10a**	0	0	0	0	0
**10b**	1	0	0	0	0
**10c**	2	1	1	0	0
**10d**	1	1	0	0	0
**10e**	2	1	0	0	0
**10f**	2	0	0	0	0
**10g**	1	1	0	0	0
**10h**	2	1	1	0	0
**10i**	1	1	0	0	0
**10j**	2	1	0	0	0

a [#] Abbreviations. QP stars: number of property or descriptor values that fall outside the 95% range of similar values for known drugs (optimal values in the range 0–5). LRoF: Lipinski rule of five. VV: Veber violations. PAINS: pan-assay interference compounds.

Comparison of the two series revealed a consistent shift in the polarity upon imidazole incorporation. In particular, compounds of type **10** displayed lower average predicted log *P*_o/w_ values (≈ 5.3) compared to the nitro-substituted analogues (≈5.8), accompanied by increased dipole moment (DM) and solvent-accessible surface area (SASA) values (Table 1). These changes indicate enhanced polarity and solvent exposure without exceeding the optimal physicochemical ranges typically associated with drug-like molecules.

The introduction of the imidazole moiety also increased the number of hydrogen-bond donors and acceptors, modulating the polarity–permeability balance while maintaining favorable membrane transport properties. Predicted QPP-Caco and QPP-MDCK values for compounds **10a**–**j** remained within acceptable ranges, and high predicted human oral absorption (HOA%) values were observed (generally >80%). None of the compounds was predicted to be a *P*-glycoprotein substrate, suggesting limited liability toward active efflux mechanisms.

Drug-likeness analysis (Table 2) further supported the suitability of the optimized series. Imidazole-containing derivatives displayed QikProp Stars values between 0 and 2, indicating that their predicted properties largely fall within the range of approved drugs. Compliance with Lipinski’s rule of five and Veber’s criteria was satisfactory, with no more than one violation per compound. Importantly, no PAINS or Brenk structural alerts were identified for type **10** derivatives.

To further assess the safety profile of the optimized imidazole-thienoquinoline derivatives **10a**–**j**, an extended in silico toxicity evaluation was performed using the ProTox-3.0 Web server. The analysis encompassed a broad range of in vitro/vivo toxicity end points, including acute oral toxicity (LD_50_ and toxicity class), organ toxicity (hepatotoxicity, neurotoxicity, cardiotoxicity, respiratory, and nephrotoxicity), as well as additional toxicological end points such as mutagenicity, carcinogenicity, cytotoxicity, immunotoxicity, and blood–brain barrier (BBB) permeability. Moreover, the platform enabled the investigation of toxicity-related pathways, molecular initiating events (MIEs), and potential off-target interactions associated with adverse effects.^28–30^

As summarized in Table S3 (see the Supporting Information), all compounds were predicted to belong to toxicity class 4, with LD_50_ values ranging from 595 to 1100 mg/kg, consistent with a moderate toxicity profile. According to the Globally Harmonized System (GHS) classification, this corresponds to compounds with relatively low acute toxicity. As further evidenced by Table S3 and Figures S1 and S2 (toxicity radar charts and predicted off-target binding profiles), out of a total of 61 evaluated toxicity end points, the investigated compounds exhibited a limited number of predicted active alerts, with an average of 4.6 alerts per compound (range 4–6 across the **10a**–**j** series), supporting an overall acceptable toxicity profile.

Within the general toxicity alerts, carcinogenicity, mutagenicity, cytotoxicity, clinical toxicity, ecotoxicity, and nutritional toxicity were consistently predicted as inactive, while immunotoxicity showed sporadic alerts. From a mechanistic perspective, no significant activation was observed across Tox21 nuclear receptor signaling and stress response pathways, with all compounds predicted as inactive.

Analysis of molecular initiating events (MIEs) further supported the absence of major liabilities, as the vast majority of targets—including key neuronal receptors (GABA, NMDA, AMPA, and kainate), endocrine receptors, and enzymatic systems—were predicted as inactive with only isolated alerts. In addition, no relevant interactions were predicted for cytochrome P450 isoforms (CYP1A2, CYP2C19, CYP2C9, CYP2D6, CYP3A4, and CYP2E1), suggesting a low risk of metabolism-related drug–drug interactions.

Consistently, off-target profiling (Figure S2) did not reveal significant binding toward a panel of 16 well-known targets associated with adverse drug effects.

The predicted toxicity profiles indicate the absence of major or systematic toxicity liabilities, supporting a favorable safety profile for the investigated compounds. Notably, the mean prediction probability was ≥ 0.75 for all compounds, further supporting the overall robustness of the toxicity assessment.

Overall, imidazole functionalization improved the predicted developability profile of the thieno­[3,2-*c*]­quinoline core without compromising key drug-likeness parameters.

### 2.2 Synthesis of the New Series of Imidazole-Containing Thieno­[3,2-*c*]­quinoline-2-carboxylates **10a**–**j**

The imidazole–thienoquinoline derivatives **10a**–**j,** selected based on their favorable predicted physicochemical and ADME profiles, were synthesized according to the route outlined in Scheme 1 through a seven-step sequence. The strategy proceeded via the previously reported nitro and amino intermediates **7a**–**e** and **16a**–**e**.^18^

**Scheme 1:**
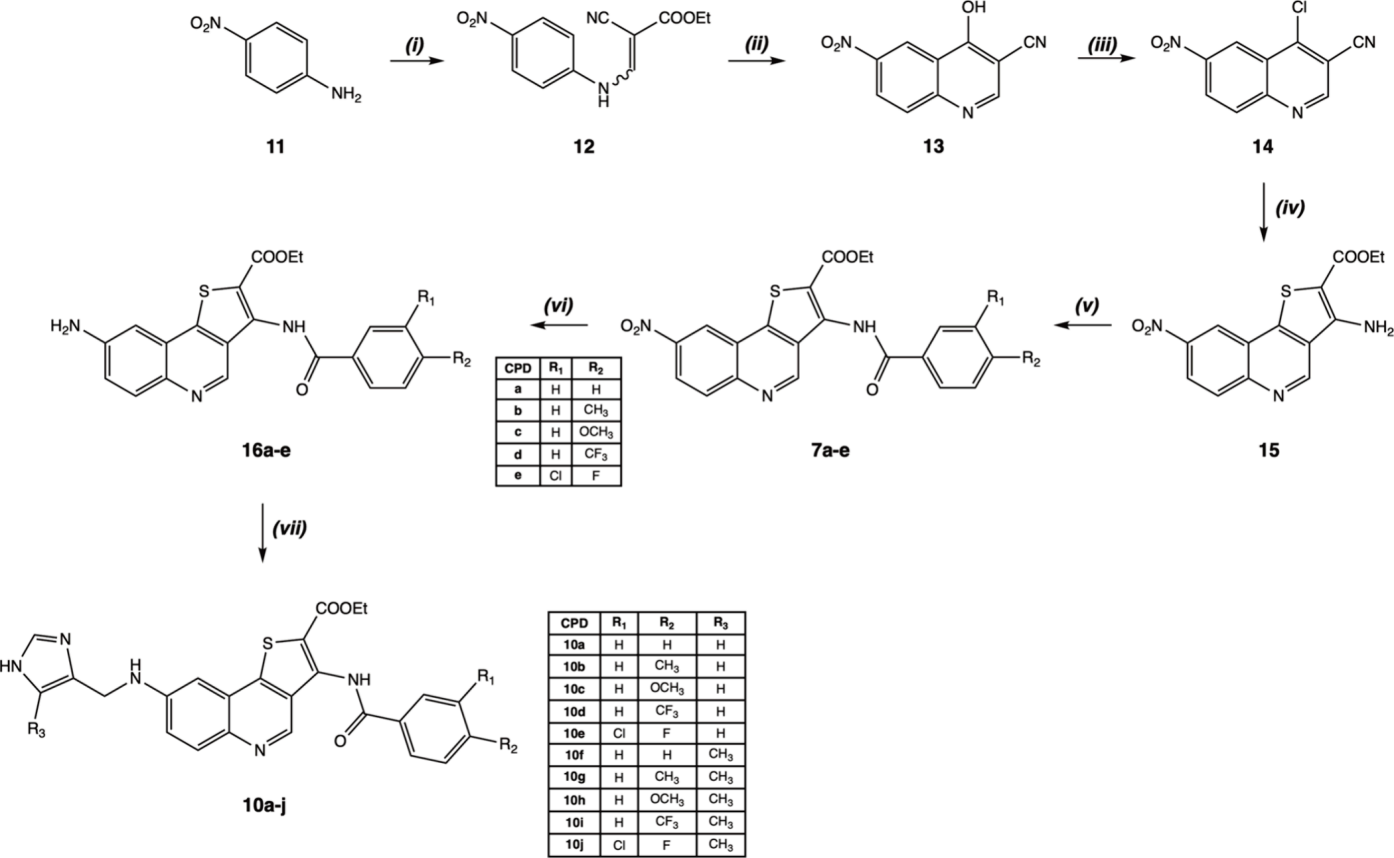
Reagents and Conditions^a^

a (i) Ethyl-2-cyano-3-ethoxyacrylate, toluene, reflux 12 h; (ii) Dowtherm A, N_2_, reflux, 10 h; (iii) POCl_3_, reflux, 8 h; (iv) ethyl thioglycolate, NEt_3_, dry DMSO, rt 2h; (v) appropriate benzoyl chloride (4 equiv), pyridine/DMAP (2:2 equiv), dry DCM, rt 12–24 h; (vi) 10% Pd/C, H_2_, ethanol, rt 24 h; (vii) 4-imidazolecarboxaldehyde or 4-methyl-5-imidazolecarboxaldehyde (1.28 equiv), NaBH_3_CN (1.28 equiv), ethanol/AcOH (cat.), rt 2 h.

Assembly of the quinoline core was achieved using the Gould–Jacobs protocol.^31^ Condensation of *p*-nitroaniline (**11**) with ethyl 2-cyano-3-ethoxyacrylate afforded intermediate **12**, which underwent thermal cyclization to yield 4-hydroxy-6-nitroquinoline **13** in high yield. Subsequent chlorination at C4 (**14**) enabled construction of the thieno­[3,2-*c*]­quinoline core via nucleophilic substitution with ethyl thioglycolate followed by intramolecular cyclization, providing intermediate **15** in quantitative yield.

Acylation of the resulting amino functionality with appropriately substituted benzoyl chlorides furnished nitro derivatives **7a**–**e**, which were converted to the corresponding amino intermediates **16a**–**e** by catalytic hydrogenation, as previously described.^18^

Introduction of the imidazole fragment was accomplished in the final step by reductive amination of intermediates **16a**–**e** with imidazole aldehydes under mild acidic conditions, using NaBH_3_CN as a reducing agent. This chemoselective transformation enabled introduction of the imidazole moiety without affecting the polycondensed thienoquinoline core. The target compounds **10a**–**j** were isolated in moderate to good yields (28–65%).

All final derivatives were fully characterized by NMR spectroscopy and high-resolution mass spectrometry, confirming the integrity of the scaffold and the successful incorporation of the imidazole unit. Detailed procedures and spectroscopic data are provided in Section 4 and in the Supporting Information (Figures S3–S24).

### 2.3 In Vitro Broad Antiproliferative Evaluation: One-to-Five Dose NCI-60 Human Tumor Cell Line Screen

The NCI-60 human cancer cell line panel represents one of the most extensively validated platforms for the preclinical evaluation of anticancer agents and has been recently updated to its advanced HTS384 version to ensure even better reliability.^32,33^ The screening panel comprises 60 human tumor cell lines organized into nine disease-oriented subpanels, including leukemia, melanoma, and tumors of the lung, colon, central nervous system (CNS), ovary, breast, prostate, and kidney.^33,34^ This system enables the systematic identification and characterization of compounds with growth-inhibitory or cytotoxic activity, representing a key opportunity for researchers working in the anticancer drug discovery area.

To assess the antiproliferative potential of the new thienoquinoline derivatives described in this study, **10a**–**j**, the complete library of synthesized compounds was submitted to the National Cancer Institute for biological evaluation. In accordance with the compound selection criteria reported in Section 4 and in ref 35, compounds **10a**–**c**,**e**–**h** were accepted and initially tested in the single-dose screening protocol, at a single concentration of 10^–5^ M.^36^

In the one dose assay, biological responses are expressed as percentage growth (G%) relative to the untreated control cells. Values above 100% indicate a lack of growth inhibition, whereas *G*% values between 0 and 100% reflect increasing levels of antiproliferative activity. Negative *G*% values indicate cytotoxic effects (i.e., net cell loss) rather than solely antiproliferative activity. For each compound, the NCI provided a bar graph and a mean graph summarizing the activity profile across the 60 tumor cell lines. The complete data set for all tested compounds is reported in the Supporting Information (Table S4). Table 3 reports, for each compound, the mean *G*% values calculated for the nine disease-oriented subpanels.

**Table 3:** Growth Percentage (*G*%) Values Determined for Compounds **10a**–**c**,**e**–**h** across the NCI-60 Human Tumor Cell Line Panel in the One-Dose Assay (10 μM)^a^

	*G*% value at 10 μM						
	10a	10b	10c	10e	10f	10g	10h
panel	(850139)	(850141)	(850143)	(850145)	(850140)	(850142)	(850144)
leukemia	–75.81	–63.62	–96.45	82.77	–94.04	–66.93	–67.35
NSCLC	–3.48	–77.45	–95.98	67.93	–87.74	–45.08	–90.67
colon cancer	–27.99	–68.13	–95.27	85.56	–92.30	–76.75	–90.47
CNS cancer	–17.51	–88.85	–97.18	67.01	–75.95	–54.81	–93.74
melanoma	–45.58	–76.43	–97.47	78.60	–97.25	–86.72	–97.22
ovarian cancer	–15.02	–72.51	–91.16	62.72	–83.14	–56.84	–81.66
renal cancer	–61.09	–92.10	–97.71	63.45	–96.80	–84.58	–97.07
prostate cancer	19.06	–52.60	–95.80	74.76	–89.82	–52.03	–90.85
breast cancer	–39.25	–94.61	–97.17	55.27	–92.26	–85.20	–97.42
overall mean *G*%	–29.63	–76.26	–96.02	70.89	–89.92	–67.66	–89.60

a For each compound (column), the mean *G*% value is reported for each of the nine disease-oriented subpanels. The corresponding NSC codes assigned by the National Cancer Institute are reported in parentheses.

Six out of the seven tested compounds (**10a**–**c,f**–**h**) exhibited marked antiproliferative and cytotoxic effects across a majority of tumor subpanels, as reflected by consistently negative *G*% values in the single-dose assay. In contrast, compound **10e**, bearing a 3-chloro-4-fluoro substitution pattern on the aromatic ring, displayed a markedly weaker activity profile with predominantly positive *G*% values and an overall mean growth percentage of approximately 70%, indicating limited antiproliferative efficacy at the tested concentration. Notably, the corresponding nitro precursor **7e** had previously shown a similar activity profile, suggesting that, in this specific substitution pattern, imidazole functionalization did not significantly enhance the antiproliferative profile. These findings point to a substitution-dependent structure–activity relationship within the series, indicating that the beneficial effect of imidazole incorporation is context-dependent.

Following the NCI screening workflow, the most active derivatives identified in the single-dose assay, **10a**–**c**,**10f**–**h**, were selected for further evaluation in the five-dose assay (from 10^–8^ to 10^–4^ M). This secondary screening enabled a quantitative assessment of their antiproliferative profiles through the determination of growth-inhibitory parameters across the entire NCI-60 panel.

The resulting dose–response data allowed the estimation of three parameters, GI_50_ (growth inhibition 50%) TGI (total growth inhibition), and LC_50_ (lethal concentration 50%) values for each compound against all 60 cancer cell lines, providing a more detailed characterization of their potency and cytotoxic potential. For each compound, graphical representations of the dose–response behavior and bar-plot summaries of the activity across the panel were also provided by the NCI and were used to support the comparative analysis of the selected derivatives (Tables S5–S7). The detailed GI_50_ values obtained for the six selected imidazole-containing thienoquinoline derivatives across the entire NCI-60 cancer cell line panel are reported in Table 4, providing a comprehensive overview of their antiproliferative potency at the individual cell line level. Submicromolar values are highlighted to facilitate the identification of the most sensitive cancer cell lines and the most active compounds.

**Table 4:**
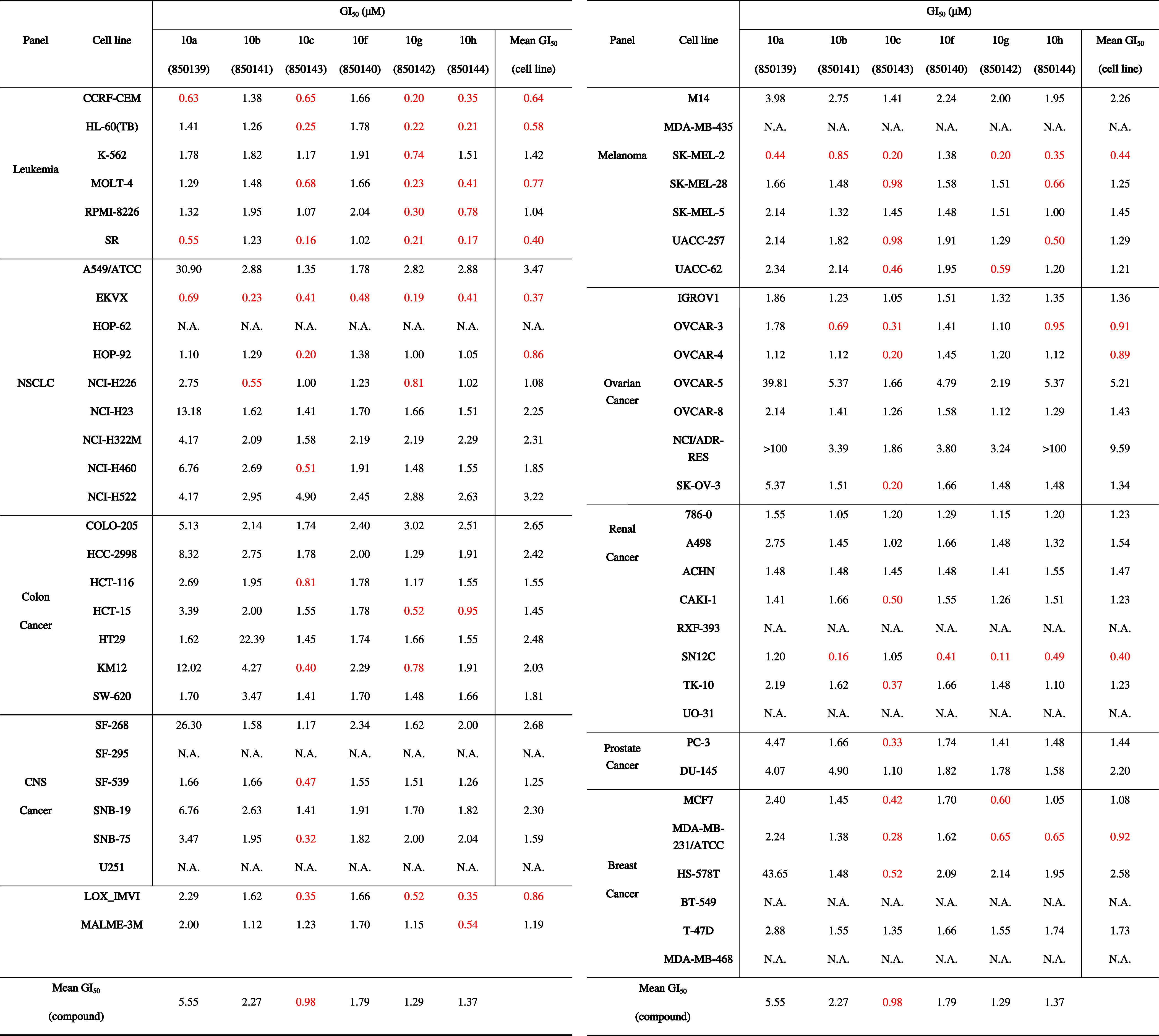
GI_50_ Values (μM) Determined for the Selected Imidazole-Containing Thienoquinoline Derivatives **10a**–**c**,**f**–**h** across the Full NCI-60 Human Tumor Cell Line Panel in the Five-Dose Assay^a^

a N.A.: not available. For each compound, the GI_50_ values obtained for all 60 individual cancer cell lines are reported. The corresponding NSC codes assigned by the National Cancer Institute are reported in parentheses. Mean values are provided both per compound (columns), representing the average GI_50_ across all tested cell lines, and per cell line (rows), corresponding to the average GI_50_ of the tested compounds against each cell line. GI_50_ values below 1 μM are highlighted in red to facilitate identification of the most sensitive cancer cell lines and the most potent compounds.

Overall, all six compounds exhibited submicromolar antiproliferative activity against a large fraction of the tested cancer cell lines (highlighted in red in Table 4), with mean GI_50_ values in the range 0.98–5.55 μM, confirming the robust antiproliferative profile suggested by the one-dose screening. Compound **10c** was the most active derivative, with a mean GI_50_ < 1 μM and potent and broad antiproliferative activity across the majority of leukemia, melanoma, renal, breast, and lung cancer cell lines. In particular, pronounced activity was observed in leukemia (HL-60­(TB), SR), NSCLC (HOP-92, EKVX), melanoma (SK-MEL-2, LOX-IMVI), ovarian (OVCAR-3, OVCAR-4), renal (SN12C), and breast cancer (MDA-MB-231) cell lines. In Figure 4, the dose–response curves provided by the NCI for compound **10c** (NSC850143) are reported.

**Figure 4:**
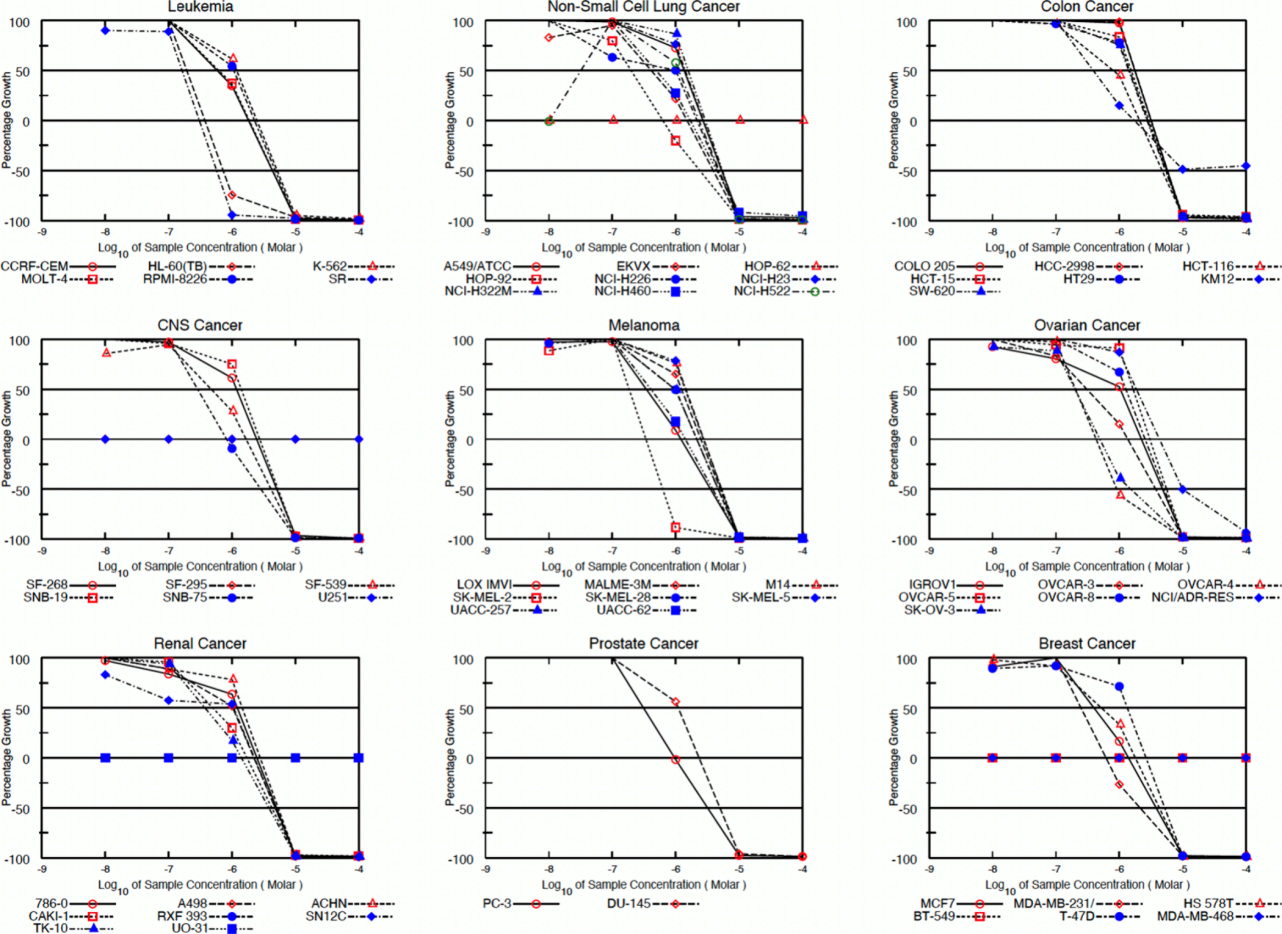
Dose–response curves provided by NCI for compound **10c** across the NCI-60.

When considering the most sensitive disease-oriented subpanels, the mean GI_50_ values of the six selected compounds were below 1 μM for the majority of cell lines within the leukemia panel, with particularly pronounced activity against the aggressive CCRF-CEM, HL-60­(TB), MOLT-4, and SR models. Submicromolar potency was also consistently observed against highly aggressive nonsmall-cell lung cancer (NSCLC) cell lines, including EKVX and HOP-92, melanoma cells LOX-IMVI and SK-MEL-2, ovarian cancer models OVCAR-3 and OVCAR-4, renal cancer SN12C, and the triple-negative breast cancer (TNBC) cell line MDA-MB-231. Notably, all of these models are representative of poorly differentiated and clinically aggressive tumors, highlighting a broad and consistent antiproliferative activity profile across several clinically aggressive tumor models.

Analysis of the TGI data (see Table S6 for the complete data set) revealed that all six compounds were able to achieve complete inhibition of cell growth at low micromolar concentrations, with TGI values generally falling in the submicromolar to low micromolar range (2–15.5 μM).

To further assess the balance between growth inhibition and nonspecific cytotoxicity, the cytostatic selectivity index (CSI; LC_50_/GI_50_) was computed; the analysis of CSI values reported in Table 5 highlights that the majority of the imidazole-containing thienoquinoline derivatives display moderate to high selectivity for growth inhibition over cell killing (see Table S7 for the complete LC_50_ data set). In particular, several compounds exhibited moderate CSI values in a range of 10–100 across multiple tumor models (highlighted in yellow), while, in selected aggressive cancer cell lines, CSI values exceeding 100 were observed—EKVX and NCI-H226 (NSCLC panel), KM12 (colon cancer panel), OVCAR-3 (ovarian cancer panel), and SN12C (renal cancer panel)—indicative of a pronounced cytostatic profile (highlighted in green), whereby the optimized derivatives retain submicromolar antiproliferative potency while requiring much higher concentrations to induce overt cytotoxicity. This behavior is consistent with a predominantly cytostatic mode of action in several tumor models, which is in line with a phenotype-driven antiproliferative profile.

**Table 5:**
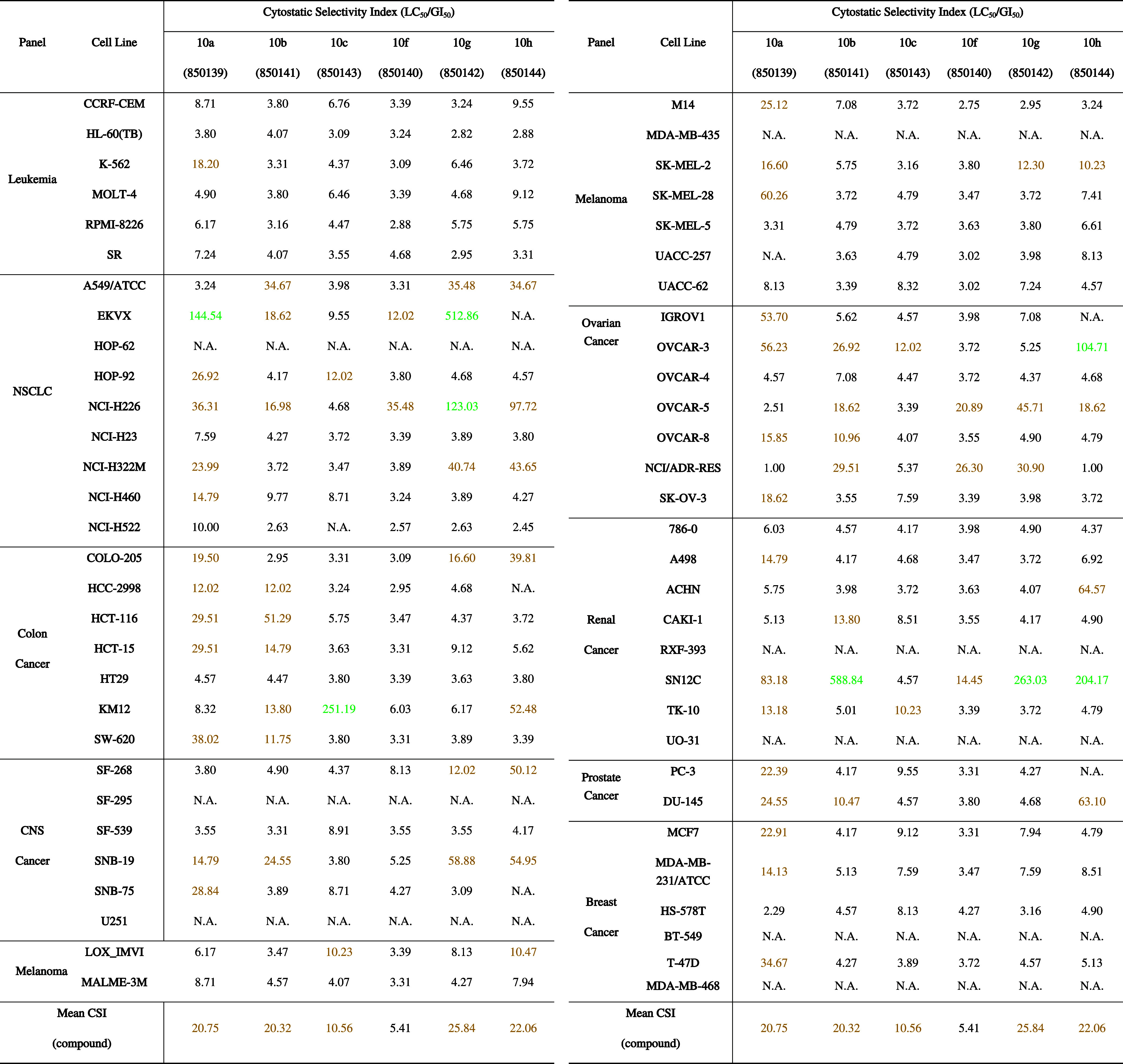
Cytostatic Selectivity Index (CSI) Values Calculated as the LC_50_/GI_50_ Ratio for Selected Imidazole-Containing Thienoquinoline Derivatives **10a**–**c**,**f**–**h** across the NCI-60 Cancer Cell Line Panel^a^

a CSI values were used to estimate the balance between growth-inhibitory (cytostatic) and cytotoxic effects. Values reported in black indicate low selectivity between cytostatic and cytotoxic activity (CSI < 10), and values highlighted in yellow correspond to moderate selectivity (10 ≤ CSI ≤ 100), whereas values highlighted in green indicate high cytostatic selectivity (CSI > 100). Mean CSI values are given for each compound (column) across the entire NCI-60.

### 2.4 In Silico Insights

#### 2.4.1 Molecular Docking and Free-Binding Energy Analysis

To gain insight into the potential interaction networks underlying the broad antiproliferative activity observed in the five-dose NCI-60 assay, computational studies were performed on compounds **10a**–**c**,**f**–**h**. The initial docking campaign was conceived to explore plausible binding modes within oncogenic targets for which thieno­[3,2-*c*]­quinoline inhibitors have been previously reported (compounds **1**–**6**, Figure 1), rather than to assign a definitive molecular target. An induced fit docking (IFD) study was then conducted against six selected proteins: PI3Kα (PDB ID: 8EXL),^37^ PI3Kγ (PDB ID: 3L08),^38^ casein kinase 2 (CK2, PDB ID: 3NGA),^39^ PIM1 kinase (PDB ID: 5O11),^40^ Mer kinase (PDB ID: 3TCP),^41^ and PARP1 (PDB ID: 7KK4).^42^ These crystal structures were selected based on the availability of cocrystallized inhibitors, enabling direct comparison with experimentally validated binding modes.

For each target, the Docking Score, Prime Energy, and IFD Score were calculated. Table 6 reports the ranges obtained for: the six type **10** derivatives evaluated in the five-dose assay (**10a**–**c**,**f**–**h**); the corresponding nitro precursors (type **7**, parent compounds); literature thienoquinoline reference inhibitors (compounds **1**–**6**) and the cocrystallized ligands. Complete data sets are provided in Table S8 (Supporting Information).

**Table 6:** Induced Fit Docking (IFD) results for compounds **10a**–**c,f**–**h** against PI3Kα (PDB ID: 8EXL), PI3Kγ (3L08), CK2 (3NGA), PIM1 (5O11), Merkinase (3TCP), and PARP1 (7KK4)­^a^

target	compound	Docking Score	Prime Energy	IFD Score
PI3Kα (PDB ID: 8EXL)*	type **10** derivatives	–12.315 ÷ −10.050	–40568.2 ÷ −40533.9	–2040.65 ÷ −2036.83
	type **7** derivatives	–11.533 ÷ −10.143	–40512.1 ÷ −40486.1	–2037.14 ÷ −2034.63
	ref. TQ inhibitor (cpd. **1**)	–9.847	–40543.4	–2037.10
	cocryst. ligand	–9.526	–40506.2	–2034.85
PI3Kγ (PDB ID: 3L08)	type **10** derivatives	–11.795 ÷ −8.264	–36420.0 ÷ −36378.8	–1832.83 ÷ −1828.39
	type **7** derivatives	–9.279 ÷ −6.958	–36363.3 ÷ −36341.0	–1826.64 ÷ −1825.12
	ref. TQ inhibitor (cpd. **2**)	–7.296	–36376.3	–1826.11
	cocryst. ligand	–11.925	–36429.2	–1833.48
Casein Kinase 2 (PDB ID: 3NGA)*	type **10** derivatives	–10.798 ÷ −9.054	–15542.2 ÷ −15510.8	–787.38 ÷ −785.15
	type **7** derivatives	–10.760 ÷ −8.652	–15488.7 ÷ −15445.7	–785.20 ÷ −780.95
	ref. TQ inhibitor (cpd. **3**)	–10.085	–15454.3	–782.80
	cocryst. ligand	–11.884	–15525.4	–788.16
PIM1 kinase (PDB ID: 5O11)*	type **10** derivatives	–10.728 ÷ −8.753	–12234.3 ÷ −12193.9	–621.70 ÷ −618.64
	type **7** derivatives	–7.052 ÷ −6.125	–12180.2 ÷ −12143.8	–615.13 ÷ −613.65
	ref. TQ inhibitor (cpd. **3**)	–7.527	–12171.9	–616.12
	cocryst. ligand	–9.040	–12197.6	–618.92
Mer kinase (PDB ID: 3TCP)	type **10** derivatives	–9.738 ÷ −8.240	–11218.8 ÷ −11185.7	–570.53 ÷ −568.05
	type **7** derivatives	–8.581 ÷ −7.658	–11197.0 ÷ −11145.7	–568.33 ÷ – 564.94
	ref. TQ inhibitor (cpd. **4**)	–8.412	–11219.3	–569.38
	cocryst. ligand	–9.970	–11305.4	–575.24
PARP1 (PDB ID: 7KK4)*	type **10** derivatives	–9.959 ÷ −8.333	–14736.7 ÷ −14715.2	–746.82 ÷ −744.18
	type **7** derivatives	–8.818 ÷ −7.432	–14696.2 ÷ −14658.2	–742.24 ÷ −740.62
	ref TQ inhibitors (cpds. **5** and **6**)	–11.892 ÷ −11.530	–14693.7 ÷ −14680.1	–746.57 ÷ −745.53
	cocryst. ligand	–13.448	–14637.3	–745.31

a Targets marked with an asterisk (*) were selected for MM-GBSA analysis. For each selected target, the best-ranked type **10** derivative was chosen as the representative ligand for MM-GBSA analysis (**10c** for PI3Kα and CK2; **10h** for PIM1 and PARP1). Cocrystallized ligands used as references were: taselisib (PI3Kα, CID 51001932), omipalisib (PI3Kγ, CID 25167777), CX-4945 (CK2/PIM1, CID 24748573), UNC569 (Mer, CID 53355503), and olaparib (PARP1, CID 23725625). Reported values represent the ranges of Docking Score, Prime Energy, and IFD Score calculated for: (i) type **10** derivatives evaluated in the five-dose NCI-60 assay, (ii) corresponding nitro precursors (type **7**), (iii) literature thienoquinoline reference inhibitors (compounds **1**–**6**), and (iv) cocrystallized ligands.

For four of the six targets (PI3Kα, CK2, PIM1, and PARP1), the imidazole-functionalized derivatives consistently exhibited more favorable Docking Score, Prime Energy, and IFD Score ranges compared to their nitro precursors (type **7**). In several cases, the calculated values were comparable to or exceeded those obtained for both literature thienoquinoline inhibitors and the respective cocrystallized ligands. These findings indicate that imidazole incorporation enhances the predicted interaction strength and conformational adaptability within catalytic sites. By contrast, PI3Kγ and Mer kinases did not show a clear improvement upon structural optimization.

Overall, the IFD results confirm in silico the positive impact of the **7** → **10** structural evolutions observed experimentally, particularly for targets implicated in oncogenic signaling in multidrug-resistant cancer phenotypes.

To further validate the predicted binding poses and refine the energetic ranking, the molecular mechanics with generalized Born and surface area (MM-GBSA) method was applied to calculate the ligand binding energies for the four most promising targets (PI3Kα, CK2, PIM1, and PARP1). For each protein, the top-ranked compound of type **10** was selected as a representative of the optimized series: compound **10c** for PI3Kα and CK2 and compound **10h** for PIM1 and PARP1.

The calculated energetic contributions, Prime Energy, Prime Coulomb, Prime Lipo, Prime Hbond, Prime Solv GB, and Prime vdW, are summarized in Table 7 and compared with those of the corresponding cocrystallized inhibitors.

**Table 7:** MM-GBSA Energetic Components Calculated for the Top-Ranked Type **10** Derivatives (**10c** for PI3Kα and CK2; **10h** for PIM1 and PARP1) in Comparison with the Respective Cocrystallized Inhibitors^b^

target	compound^a^	Prime Energy	Prime Coulomb	Prime Lipo	PrimeH-bond	Prime Solv GB	Prime vdW
PI3Kα (PDB ID: 8EXL)	10c	–40568.2	–28257.74	–5370.41	–420.16	–5243.1	–5090.4
	cocryst. ligand	–40506.2	–28343.85	–5364.23	–425.44	–5113.42	–5104.46
casein kinase 2 (PDB ID: 3NGA)	10c	–15529.8	–10527.53	–2042.36	–197.15	–2409.44	–1872.39
	cocryst. ligand	–15525.4	–10604.88	–2016.33	–190.81	–2324.94	–1874.85
PIM1 kinase (PDB ID: 5O11)	10h	–12234.3	–8463.91	–1757.41	–138.06	–1764.71	–1419.26
	cocryst. ligand	–12197.6	–8323.29	–1715.8	–138.55	–1878.27	–1397.48
PARP1 (PDB ID: 7KK4)	10h	–14736.7	–10605.08	–1900.39	–132.06	–1886.76	–1746.25
	cocryst. ligand	–14637.3	–10611.77	–1900.33	–133.12	–1800.61	–1746.43

a Cocrystallized ligands used as references were: taselisib (PI3Kα, CID 51001932), CX-4945 (CK2/PIM1, CID 24748573), and olaparib (PARP1, CID 23725625).

b Reported parameters include Prime Energy, Prime Coulomb, Prime Lipo, Prime Hbond, Prime Solv GB, and Prime vdW contributions (kcal/mol unit).

In all four targets, compounds **10c** and **10h** displayed interaction energy profiles comparable to and in some cases more favorable than those of the reference ligands. Enhanced Coulombic and hydrogen-bond contributions were observed for the imidazole-containing derivatives, consistent with their increased polarity and hydrogen-bonding capacity. The van der Waals and lipophilic terms remained favorable, reflecting preservation of the hydrophobic anchoring provided by the thienoquinoline core.

These results support the ability of the optimized derivatives to form stable protein–ligand complexes and corroborate the improved docking performance observed in the IFD analysis.

#### 2.4.2 Molecular Dynamics Simulation Studies: Ligand-Protein Complex Stability and Interaction Pattern Analysis

To further evaluate the dynamic stability of the predicted binding modes, 100 ns molecular dynamics simulations were performed for the top-ranked complexes of PI3Kα-**10c**, CK2-**10c**, PIM1-**10h**, and PARP1-**10h**. The corresponding cocrystallized inhibitors were simulated under identical conditions to enable direct comparison (Figure 5; see also Figure S25).

**Figure 5:**
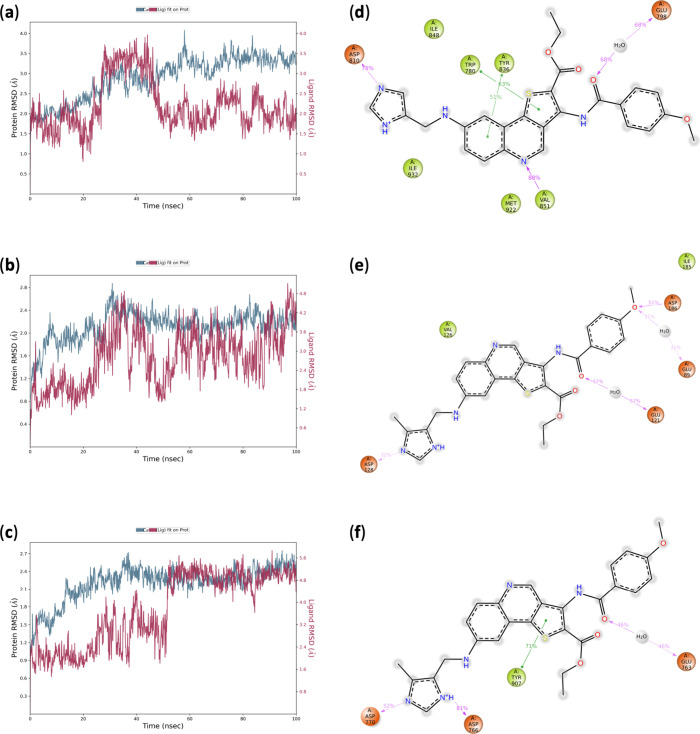
Molecular dynamics (100 ns) analysis of the selected protein–ligand complexes. (a–c) Left panels: protein backbone RMSD (blue) and ligand RMSD (red) plotted over simulation time for PI3Kα-**10c**, PIM1-**10h**, and PARP1-**10h** systems. (d–f) Right panels: representative protein–ligand interaction diagrams showing persistent hydrogen bonds, electrostatic, and hydrophobic contacts observed during the trajectories, for PI3Kα-10c, PIM1-10h, and PARP1-10h systems, respectively.

For PI3Kα-**10c**, an initial conformational adaptation phase was observed during the first ∼40 ns, followed by stabilization of the protein backbone RMSD around 3.0–3.5 Å. The ligand RMSD remained within ∼2.0–2.5 Å after equilibration, indicating retention within the catalytic pocket. Interaction analysis revealed persistent hydrogen bonds and electrostatic contacts involving residues such as Trp780, Glu798, Asp810, Tyr836, and Val851 alongside stable hydrophobic interactions with aromatic residues in the ATP-binding region. The behavior closely mirrored that of the cocrystallized inhibitor, supporting a comparable binding mode.

In the PIM1-**10h** system, the protein backbone RMSD stabilized around 2.0–2.3 Å after initial equilibration. The ligand RMSD exhibited moderate fluctuations, reaching values up to ∼3.5–4.0 Å during the simulation, indicative of conformational adjustments within the binding pocket. Persistent hydrogen-bond interactions and hydrophobic contacts were maintained throughout the simulation, with interaction occupancies comparable to those observed for the reference ligand. These data indicate a stable and well-accommodated binding pose.

For PARP1-**10h**, moderate ligand RMSD fluctuations were observed; however, key hydrogen-bond interactions remained preserved during the simulation time and the ligand remained anchored within the nicotinamide-binding pocket. The overall dynamic profile was consistent with a stable protein–ligand complex and comparable to that of the cocrystallized inhibitor.

In contrast, the CK2-**10c** complex exhibited substantial ligand RMSD fluctuations (>8 Å), indicating significant positional instability during the simulation (not shown).

Collectively, the MD simulations confirm the dynamic stability of selected imidazole-functionalized thienoquinolines within the PI3Kα, PIM1, and PARP1 catalytic domains, supporting the hypothesis that scaffold optimization enhances interaction persistence under physiologically relevant conditions. Moreover, trajectory analyses demonstrated that the imidazole nucleus plays a key role in establishing and maintaining hydrogen-bond and electrostatic interactions, particularly with charged and polar residues within the catalytic pockets, thereby contributing to the overall stability and adaptability of the complexes.

#### 2.4.3 Density Functional Theory (DFT) Analysis

To further rationalize the positive electronic impact of imidazole incorporation, density functional theory (DFT) calculations were performed on compounds **10c** and **10h**, selected as representative members of the optimized imidazole-functionalized series, and on the corresponding nitro precursor **7c**, a representative of the parent chemotype. Frontier molecular orbital (FMO) energies and global reactivity descriptors derived from Koopmans’ approximation are summarized in Table 8, while the molecular electrostatic potential (MEP) surfaces are shown in Figure 6.

**Table 8:** Frontier Molecular Orbital Energies (HOMO and LUMO) and Global Reactivity Descriptors Calculated at the DFT Level for Representative Compounds **7c**, **10c**, and **10h** ^a^

compound	HOMO	LUMO	Δ*E*	μ	η	*S*	ω
**7c**	–0.281817	–0.073481	0.208336	–0.177649	0.104168	4.80	0.151
**10c**	–0.320043	–0.161914	0.158129	–0.240979	0.079065	6.33	0.367
**10h**	–0.321343	–0.153759	0.167584	–0.237551	0.083792	5.97	0.337

a The HOMO–LUMO gap (Δ*E*), chemical potential (μ), chemical hardness (η), softness (*S*), and electrophilicity index (ω) were derived using Koopmans’ approximation from frontier orbital energies.

**Figure 6:**
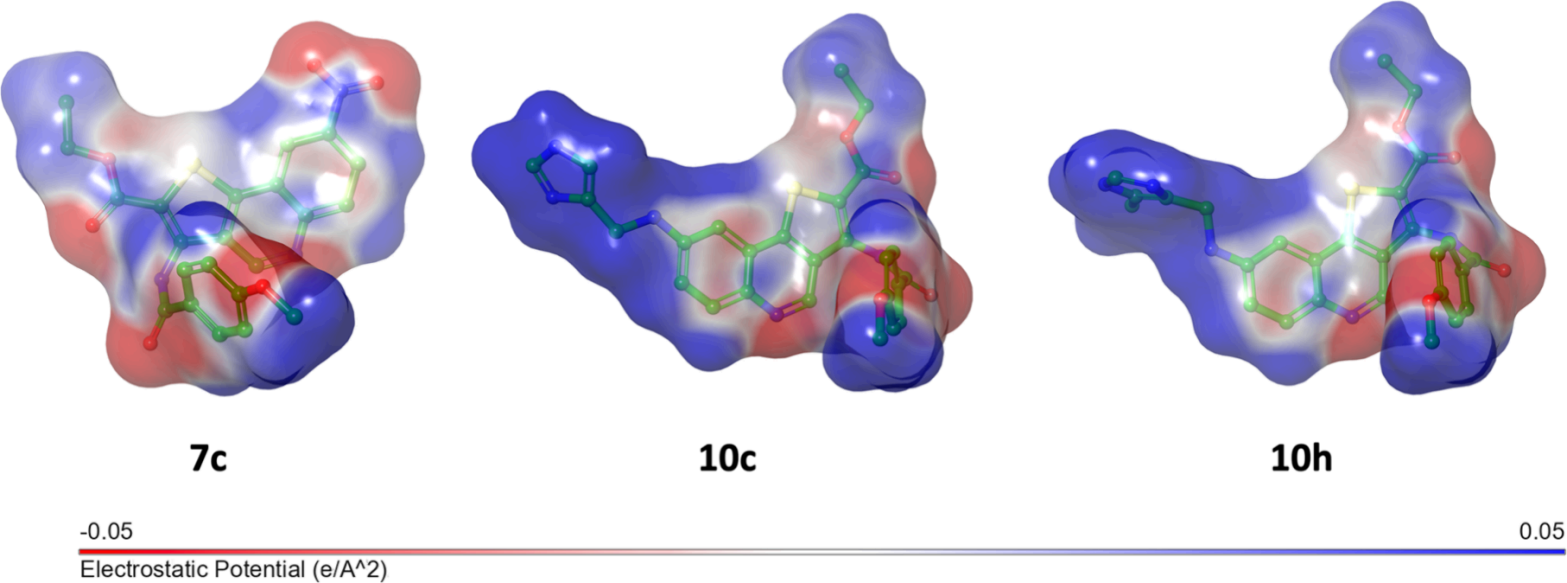
Molecular electrostatic potential (MEP) surfaces of representative compounds **7c** (left), **10c** (center), and **10h** (right) mapped onto the electron density isosurface at the DFT-optimized geometries. The color scale ranges from −0.05 (red, negative electrostatic potential) to +0.05 (blue, positive electrostatic potential) e·Å^–2^.

Relative to nitro precursor **7c**, both **10c** and **10h** exhibited more negative HOMO and LUMO energies, indicating increased electronic stabilization of the optimized structures. Notably, the lower LUMO energies observed for the imidazole-containing derivatives are reflected in a reduced HOMO–LUMO gap (Δ*E*) and decreased chemical hardness (η), accompanied by an increase in global softness (*S*). This trend suggests enhanced electronic polarizability of the optimized scaffold. Furthermore, the electrophilicity index (ω) is markedly higher for **10c** and **10h** compared to that of **7c**, indicating a greater propensity to participate in electrostatic interactions within protein environments.

MEP surface analysis (Figure 6) highlights a redistribution of the electrostatic potential upon imidazole incorporation. In compounds **10c** and **10h**, the imidazole moiety displays an extended region of positive electrostatic potential, reflecting its polarized character within the conjugated framework. In contrast, compound **7c** shows a more localized electrostatic distribution primarily associated with the nitro group. Importantly, this electronic reorganization occurs without disrupting the extended π-conjugated thienoquinoline core.

Overall, the DFT results indicate that imidazole functionalization enhances the electronic adaptability and the polar interaction capacity of the scaffold. These features are consistent with the persistent electrostatic and hydrogen-bond interactions observed in docking and molecular dynamics simulations, particularly within the catalytic domains of analyzed proteins, thereby supporting the hypothesis of potential multitarget engagement.

## 3 Conclusions

In this study, a new series of imidazole-functionalized thieno­[3,2-*c*]­quinoline-2-carboxylate hybrids were developed starting from previously reported derivatives of type **7** through a rational scaffold optimization strategy. Introduction of the imidazole fragment, conceived within a molecular hybridization approach, enabled the combination of two pharmacologically relevant motifs within a single framework. The incorporation of this privileged heterocycle modulated the polarity and hydrogen-bonding capacity while preserving the rigid polycondensed heteroaromatic core responsible for the antiproliferative profile. In silico ADME analyses indicated improved predicted pharmacokinetic and drug-like properties in the optimized series. Furthermore, extended in silico toxicity profiling revealed a limited number of predicted toxicity alerts, with no significant interactions observed toward off-targets commonly associated with adverse drug effects, supporting a favorable predicted safety profile.

Biological evaluation in the NCI-60 human tumor cell line panel revealed broad antiproliferative activity, with six derivatives displaying submicromolar mean GI_50_ values. Compound **10c** emerged as the most potent analogue, showing consistent activity across multiple aggressive tumor models. Cytostatic selectivity analysis suggested a predominantly growth-inhibitory profile in several cell lines.

Notably, the broad and consistent submicromolar antiproliferative activity observed across multiple NCI-60 subpanels provides experimental validation of the molecular hybridization strategy and highlights the translational relevance of imidazole incorporation within the thienoquinoline framework.

Complementary computational investigations, including docking, MM-GBSA calculations, molecular dynamics simulations, and DFT analysis, supported enhanced predicted binding properties and electronic adaptability of the imidazole-functionalized core relative to those of the parent series. While these results do not assign a specific molecular target, they are consistent with a multitarget interaction potential compatible with the observed broad phenotypic activity. Future studies will be aimed at experimentally assessing selectivity toward nontumoral cell lines to further validate the safety profile of this compound class.

Overall, the present study identifies imidazole-functionalized thienoquinolines as promising anticancer scaffolds and provides a coherent framework for further structure–activity optimization and mechanistic investigations.

## 4 Materials and Methods

### 4.1 Chemistry

#### 4.1.1 General Information

All chemicals and solvents were obtained from commercial suppliers and used as received, unless otherwise specified. Melting points were measured in open capillaries using a Büchi Tottoli apparatus and are reported uncorrected.

^1^H and ^13^C NMR and bidimensional spectra were recorded at 400 and 100 MHz, respectively, on a Bruker AC-E 400 MHz spectrometer in CDCl_3_ or DMSO-*d*_6_. Chemical shifts (δ) are expressed in parts per million (ppm) and referenced to tetramethylsilane (TMS) as the internal standard. Signal multiplicities are indicated as follows: br s (broad singlet), s (singlet), d (doublet), t (triplet), q (quartet), and m (multiplet); rt denotes room temperature.

The purity of all compounds evaluated in biological assays was confirmed to be ≥95% by HPLC/MS analysis. Mass spectrometry was performed by using an Agilent 6540 UHD accurate-mass quadrupole time-of-flight (Q-TOF) spectrometer.

Thin-layer chromatography (TLC) was carried out on silica gel GF254 plates (0.25 mm thickness) and visualized under UV light at 254 nm. Flash column chromatography was performed using Merck silica gel (230–400 mesh ASTM) or a Biotage FLASH40i automated system equipped with prepacked cartridges.

All compounds up to the amino intermediates **16a**–**e** were synthesized according to established literature methods reported by us, and their spectroscopic and analytical data were consistent with previously reported values.^18^ The final step (vii) was carried out as described below.

#### 4.1.2 General Procedure for the Synthesis of Ethyl 8-(((1*H*-Imidazol-4-yl)­methyl)­amino)-3-benzoylamino-thieno­[3,2-*c*]­quinoline-2-carboxylates (**10a**–**j**)

To a stirred suspension of 8-amino-thieno­[3,2-*c*]­quinolines **16a**–**e** (1 equiv) and the appropriate imidazole aldehyde (1.28 equiv) in ethanol, acetic acid was added to adjust the pH to 4–5. The reaction mixture was stirred for 1 h, after which NaBH_3_CN (1.28 equiv) was added, and stirring was continued at room temperature for 12–24 h. Upon completion, the solvent was removed under reduced pressure, and the crude product was purified by column chromatography using dichloromethane/methanol (2–15%) as an eluent. The purified compounds were subsequently recrystallized from diethyl ether/ethanol (2:1).

##### 4.1.2.1 Ethyl 3-Benzoylamino-8-(((1*H*-imidazol-4-yl)­methyl)­amino)-thieno­[3,2-*c*]­quinoline-2-carboxylate (**10a**)

Yield 45%. Mp 145–147 °C. ^1^H NMR (DMSO-*d*_6_) δ: 1.25 (t, 3H, *J* = 7.1 Hz, CH_3_), 4.26–4.39 (m, 4H, 2 × CH_2_), 6.77 (t, 1H, *J* = 5.4 Hz, NH), 7.03 (d, 1H, *J* = 2.6 Hz, H-9), 7.07 (s, 1H, H-5″), 7.33 (dd, 1H, *J* = 9.1, 2.5 Hz, H-7), 7.57–7.70 (m, 4H, H-3′, H-4′, H-5′, H-2″), 7.85 (d, 1H, *J* = 9.0 Hz, H-6), 8.06–8.14 (m, 2H, H-2′, H-6′), 8.85 (s, 1H, H-4), 10.68 (s, 1H, NH), 11.92 (br s, 1H, NH). ^13^C NMR (DMSO-*d*_6_) δ: 14.5 (CH_3_), 39.4 (CH_2_), 61.9 (CH_2_), 98.5 (CH), 121.4, 121.6 (CH), 124.8, 128.4 (CH), 129.1 (CH), 129.6, 131.0 (CH), 132.7 (CH), 134.0, 135.5 (CH), 137.5, 138.1, 141.7, 141.9 (CH), 148.6, 162.0, 166.1. HRMS-ESI [(M+H)^+^]: *m*/*z* calculated for C_25_H_21_N_5_O_3_S: 472.1438; found: 472.1436.

##### 4.1.2.2 Ethyl 8-(((1*H*-Imidazol-4-yl)­methyl)­amino)-3-(4-methylbenzamido)­thieno­[3,2-*c*]­quinoline-2-carboxylate (**10b**)

Yield 42%. Mp 150–152 °C. ^1^H NMR (DMSO-*d*_6_) δ: 1.25 (t, 3H, *J* = 7.1 Hz, CH_3_), 2.42 (s, 3H, CH_3_), 4.26–4.36 (m, 4H, 2 × CH_2_), 6.77 (t, 1H, *J* = 5.4 Hz, NH), 7.02 (d, 1H, *J* = 2.5 Hz, H-9), 7.07 (d, 1H, H-5″), 7.33 (dd, 1H, *J* = 9.1, 2.5 Hz, H-7), 7.40 (d, 2H, *J* = 8.0 Hz, H-3′, H-5′), 7.63 (d, 1H, H-2″), 7.85 (d, 1H, *J* = 9.0 Hz, H-6), 8.00 (d, 2H, *J* = 8 Hz, H-2′, H-6′), 8.84 (s, 1H, H-4), 10.60 (s, 1H, NH), 12.00 (br s, 1H, NH). ^13^C NMR (DMSO-*d*_6_) δ: 14.5 (CH_3_), 21.6 (CH_3_), 39.5 (CH_2_), 61.9 (CH_2_), 98.4 (CH), 121.1, 121.6 (CH), 124.8, 128.4 (CH), 129.6 (CH), 130.9 (CH), 131.1, 135.5 (CH), 137.7, 141.7, 142.0 (CH), 142.9, 148.6, 162.1, 165.9. HRMS-ESI [(M+H)^+^]: *m*/*z* calculated for C_26_H_23_N_5_O_3_S: 486.1594; found: 486.1595.

##### 4.1.2.3 Ethyl 8-(((1*H*-Imidazol-4-yl)­methyl)­amino)-3-(4-methoxybenzoylamino)­thieno­[3,2-*c*]­quinoline-2-carboxylate (**10c**)

Yield 48%. Mp 154–155 °C. ^1^H NMR (DMSO-*d*_6_) δ: 1.25 (t, 3H *J* = 7.1 Hz, CH_3_), 3.87 (s, 3H, OCH_3_), 4.26–4.36 (m, 4H, 2 × CH_2_), 6.76 (t, 1H, *J* = 5.4 Hz, NH), 7.02 (d, 1H, *J* = 2.5 Hz, H-9), 7.07 (s, 1H, H-5″), 7.13 (d, 2H, *J* = 8.8 Hz, H-3′, H-5′), 7.33 (dd, 1H, *J* = 9.1, 2.5 Hz, H-7), 7.62 (s, 1H, H-2″), 7.85 (d, *J* = 9.0 Hz, 1H, H-6), 8.08 (d, 2H, *J* = 8.7 Hz, H-2′, H-6′), 8.84 (s, 1H, H-4), 10.54 (s, 1H, NH), 11.97 (br s, 1H, NH). ^13^C NMR (DMSO-*d*_6_) δ: 14.5 (CH_3_), 39.5 (CH_2_), 56.0 (CH_3_), 61.9 (CH_2_), 98.4 (CH), 114.4 (CH), 120.7, 121.6 (CH), 124.8, 126.0, 129.6, 130.4 (CH), 130.9 (CH), 135.5 (CH), 138.0, 141.7, 142.1 (CH), 148.6, 162.1, 162.9, 165.4. HRMS-ESI [(M+H)^+^]: *m*/*z* calculated for C_26_H_23_N_5_O_4_S: 502.1544; found: 502.1542.

##### 4.1.2.4 Ethyl 8-(((1*H*-Imidazol-4-yl)­methyl)­amino)-3-(4-trifluoromethylbenzoylamino)­thieno­[3,2-*c*]­quinoline–2-carboxylate (**10d**)

Yield 28%. Mp 192–195 °C. ^1^H NMR (400 MHz, DMSO-*d*_6_) δ: 1.25 (t, 3H, *J* = 7.1 Hz, CH_3_), 4.27–4.36 (m, 4H, 2 × CH_2_), 6.77 (t, 1H, *J* = 5.4 Hz, NH), 7.04 (d, 1H, *J* = 2.5 Hz, H-9), 7.07 (s, 1H, H-5″), 7.34 (dd, 1H, *J* = 9.1, 2.5 Hz, H-7), 7.62 (s, 1H, H-2″), 7.86 (d, 1H, *J* = 9.1 Hz, H-6), 7.99 (d, 2H, *J* = 8.3 Hz, H-3′, H-5′), 8.28 (d, 2H, *J* = 8.1 Hz, H-2′, H-6′), 8.87 (s, 1H, H-4), 10.93 (s, 1H, NH), 11.96 (s, 1H, NH). ^13^C NMR (DMSO-*d*_6_) δ: 14.5 (CH_3_), 39.5 (CH_2_), 61.9 (CH_2_), 98.4 (CH), 121.7 (CH), 122.3, 124.8, 126.1, 126.1, 129.3 (CH), 129.7, 131.0 (CH), 132.5, 135.5 (CH), 136.8, 137.9, 138.1, 141.6 (CH), 148.6, 161.7, 165.2. HRMS-ESI [(M+H)^+^]: *m*/*z* calculated for C_26_H_20_F_3_N_5_O_3_S: 540.1312; found: 540.1313.

##### 4.1.2.5 Ethyl 8-(((1*H*-Imidazol-4-yl)­methyl)­amino)-3-(3-chloro-4-fluorobenzamido)­thieno­[3,2-*c*]­quinoline-2-carboxylate (**10e**)

Yield 37%. Mp 207–210 °C. ^1^H NMR (DMSO-*d*_6_) δ: 1.26 (t, 3H, *J* = 7.1 Hz, CH_3_), 4.27–4.36 (m, 4H, 2 × CH_2_), 6.75 (t, 1H, *J* = 5.4 Hz, NH), 7.03 (d, 1H, *J* = 2.5 Hz, H-9), 7.07 (s, 1H, H-5″), 7.34 (dd, 1H, *J* = 9.1, 2.5 Hz, H-7), 7.62 (s, 1H, H-2″), 7.66 (t, 1H, *J* = 8.9 Hz, H-5′), 7.85 (d, 1H, *J* = 9.1 Hz, H-6), 8.11 (ddd, 1H, *J* = 8.7, 4.7, 2.2 Hz, H-6′), 8.32 (dd, 1H, *J* = 7.1, 2.2 Hz, H-2′), 8.85 (s, 1H, H-4), 10.80 (s, 1H, NH), 11.91 (br s, 1H, NH). ^13^C NMR (DMSO-*d*_6_) δ: 14.5 (CH_3_), 40.5 (CH_2_), 61.9 (CH_2_), 98.3, 117.8 (d, *J* = 21.5 Hz, CH), 120.3, 120.4, 121.7 (CH), 122.2, 124.7, 129.6, 129.7 (d, *J* = 8.3 Hz, CH), 130.9 (CH), 131.7, 135.5 (CH), 136.8, 138.1, 141.6 (CH), 141.6, 148.6, 159.9 (d, *J* = 253.1 Hz), 161.6, 164.0. HRMS-ESI [(M+H)^+^]: *m*/*z* calculated for C_25_H_19_ClFN_5_O_3_S: 524.0954; found: 524.0954.

##### 4.1.2.6 Ethyl 3-Benzoylamino-8-(((5-methyl-1H-imidazol-4-yl)­methyl)­amino)­thieno­[3,2-*c*]­quinoline-2-carboxylate (**10f**)

Yield 65%. Mp 148–149 °C. ^1^H NMR (DMSO-*d*_6_) δ: 1.25 (t, 3H, *J* = 7.1 Hz, CH_3_), 2.25 (s, 3H, CH_3_), 4.24–4.36 (m, 4H, 2 × CH_2_), 6.67 (t, 1H, *J* = 5.2 Hz, NH), 7.03 (d, 1H, *J* = 2.5 Hz, H-9), 7.33 (dd, 1H, *J* = 9.1, 2.5 Hz, H-7), 7.48 (s, 1H, H-2″), 7.56–7.71 (m, 3H, H-3′, H-4′, H-5′), 7.84 (d, 1H *J* = 9.0 Hz, H-6), 8.06–8.14 (m, 2H, H-2′, H-6′), 8.84 (s, 1H, H-4), 10.68 (s, 1H, NH), 11.75 (br s, 1H, NH). ^13^C NMR (DMSO-*d*_6_) δ: 14.5 (CH_3_), 19.0 (CH_3_), 39.5 (CH_2_), 61.9 (CH_2_), 98.1 (CH), 121.4, 121.8 (CH), 124.8, 128.4 (CH), 129.1 (CH), 129.6, 130.9 (CH), 132.7 (CH), 133.7 (CH), 134.0, 137.5, 138.1, 141.6, 141.8 (CH), 148.6, 162.0, 166.1. HRMS-ESI [(M+H)^+^]: *m*/*z* calculated for C_26_H_23_N_5_O_3_S: 486.1954; found: 486.1955.

##### 4.1.2.7 Ethyl 8-(((5-Methyl-1H-imidazol-4-yl)­methyl)­amino)-3-(4-methylbenzoylamino)­thieno­[3,2-*c*]­quinoline-2-carboxylate (**10g**)

Yield 33%. Mp 157–159 °C. ^1^H NMR (DMSO-*d*_6_) δ: 1.25 (t, 3H, *J* = 7.1 Hz, CH_3_), 2.30 (s, 3H, CH_3_), 2.42 (s, 3H, CH_3_), 4.26–4.36 (m, 4H, 2 × CH_2_), 6.72 (t, 1H, *J* = 5.2 Hz, NH), 7.03 (d, 1H, *J* = 2.4 Hz, H-9), 7.31 (dd, 1H, *J* = 9.1, 2.5 Hz, H-7), 7.40 (d, 2H, *J* = 7.9 Hz, H-3′, H-5′), 7.86 (d, 1H, *J* = 9.1 Hz, H-6), 7.95–8.03 (m, 3H, H-2′, H-6′, H-2″), 8.86 (s, 1H, H-4), 10.60 (s, 1H, NH), 11.90 (br s, 1H, NH). ^13^C NMR (DMSO-*d*_6_): 10.2 (CH_3_), 14.5 (CH_3_), 21.6 (CH_3_), 38.7 (CH_2_), 61.9 (CH_2_), 98.4 (CH), 121.1, 121.7 (CH), 124.8, 126.1, 128.4 (CH), 129.2, 129.6 (CH), 131.0 (CH), 131.1, 133.6 (CH), 137.7, 138.2, 141.7, 142.2 (CH), 142.9, 148.3, 162.1, 165.9. HRMS-ESI [(M+H)^+^]: *m*/*z* calculated for C_27_H_25_N_5_O_3_S: 500.1751; found: 500.1750.

##### 4.1.2.8 Ethyl 8-(((5-Methyl-1*H*-imidazol-4-yl)­methyl)­amino)-3-(4-methoxybenzoylamino)­thieno­[3,2-*c*] quinoline-2-carboxylate (**10h**)

Yield 51%. Mp 200–202 °C. ^1^H NMR (DMSO-*d*_6_) δ: 1.25 (t, 3H, *J* = 7.1 Hz, CH_3_), 2.25 (s, 3H, CH_3_), 3.87 (s, 3H, OCH_3_), 4.23–4.36 (m, 4H, 2 × CH_2_), 6.68 (t, 1H, *J* = 5.1 Hz, NH), 7.02 (d, 1H, *J* = 2.6 Hz, H-9), 7.13 (d, 2H, *J* = 8.9 Hz, H-3′, H-5′), 7.32 (dd, 1H, *J* = 9.1, 2.5 Hz, H-7), 7.56 (s, 1H, H-2″), 7.83 (d, 1H, *J* = 9.1 Hz, H-6), 8.08 (d, 2H, *J* = 8.8 Hz, H-2′, H-6′), 8.83 (s, 1H, H-4), 10.54 (s, 1H, NH), 11.97 (br s, 1H, NH). ^13^C NMR (DMSO-*d*_6_) δ: 10.5 (CH_3_), 14.5 (CH_3_), 39.5 (CH_2_), 56.0 (CH_3_), 61.9 (CH_2_), 98.2 (CH), 114.4 (CH), 120.6, 121.7 (CH), 124.8, 126.1, 129.6, 130.4 (CH), 130.9 (CH), 133.7 (CH), 138.0, 141.7, 142.1 (CH), 148.6, 162.2, 162.9, 165.4. HRMS-ESI [(M+H)^+^]: *m*/*z* calculated for C_27_H_25_N_5_O_4_S: 516.1700; found: 516.1700.

##### 4.1.2.9 Ethyl 8-(((5-Methyl-1*H*-imidazol-4-yl)­methyl)­amino)-3-(4-(trifluoromethyl)­benzamido)­thieno­[3,2-*c*]­quinoline-2-carboxylate (**10i**)

Yield 30%. Mp. ^1^H NMR (400 MHz, DMSO-*d*_6_) δ: 1.25 (t, 3H, *J* = 7.1 Hz, CH_3_), 2.25 (s, 3H, CH_3_), 4.22–4.36 (m, 4H, 2 × CH_2_), 6.67 (t, 1H, *J* = 5.1 Hz, NH), 7.04 (d, 1H, *J* = 2.5 Hz, H-9), 7.33 (dd, 1H, *J* = 9.1, 2.5 Hz, H-7), 7.48 (s, 1H, H-2″), 7.84 (d, 1H, *J* = 9.0 Hz, H-6), 7.99 (d, 2H, *J* = 8.2 Hz, H-2′, H-6′), 8.28 (d, 2H, *J* = 8.1 Hz, H-3′, H-5′), 8.87 (s, 1H, H-4), 10.94 (s, 1H, NH), 11.77 (s, 1H, NH). ^13^C NMR (DMSO-*d*_6_) δ: 10.5 (CH_3_), 14.5 (CH_3_), 39.5 (CH_2_), 61.9 (CH_2_), 98.1 (CH), 121.8 (CH), 124.8, 126.1 (CH), 129.3 (CH), 129.7, 130.9 (CH), 133.7 (CH), 136.8, 138.0, 141.5 (CH), 141.6, 148.6, 161.8, 165.2. HRMS-ESI [(M+H)^+^]: *m*/*z* calculated for C_27_H_22_F_3_N_5_O_3_S: 554.1468; found: 554.1470.

##### 4.1.2.10 Ethyl 3-(3-Chloro-4-fluorobenzamido)-8-(((5-methyl-1*H*-imidazol-4-yl)­methyl)­amino)­thieno­[3,2-*c*]­quinoline-2-carboxylate (**10j**)

Yield 32%. Mp 149–152 °C. ^1^H NMR (DMSO-*d*_6_) δ: 1.25 (t, 3H, *J* = 7.1 Hz, CH_3_), 2.26 (s, 3H, CH_3_), 4.25–4.38 (m, 4H, 2 × CH_2_), 6.68 (t, 1H, *J* = 5.2 Hz, NH), 7.04 (d, 1H, *J* = 2.5 Hz, H-9), 7.32 (dd, 1H, *J* = 9.1, 2.5 Hz, H-7), 7.63 (s, 1H, H-2″), 7.67 (t, 1H, *J* = 8.9 Hz, H-5′), 7.85 (d, 1H, *J* = 9.0 Hz, H-6), 8.11 (ddd, 1H, *J* = 8.7, 4.7, 2.3 Hz, H-6’), 8.32 (dd, 1H, *J* = 7.1, 2.2 Hz, H-2′), 8.85 (s, 1H, H-4), 10.80 (s, 1H, NH), 12.01 (br s, 1H, NH). ^13^C NMR (DMSO-*d*_6_) δ: 10.4 (CH_3_), 14.5 (CH_3_), 39.2 (CH_2_), 61.9 (CH_2_), 98.1 (CH), 117.8 (d, *J* = 21.7 Hz, CH), 120.3, 120.5, 121.8 (CH), 122.3, 124.7, 129.6, 129.7 (d, *J* = 9.0 Hz, CH), 130.9 (CH), 131.7, 133.7 (CH), 136.7, 138.1, 141.6 (CH), 148.5, 159.9 (d, *J* = 253.1 Hz), 161.7, 164.0. HRMS-ESI [(M+H)^+^]: *m*/*z* calculated for C_26_H_21_ClFN_5_O_3_S: 538.1110; found: 538.1109.

### 4.2 NCI-60 Antiproliferative Screening

#### 4.2.1 Compound Selection Guidelines and Sample Preparation for Screening

The compounds submitted for biological screening were evaluated and selected by the National Cancer Institute (NCI) according to its established and stringent eligibility criteria. The authors provided chemical structure, stereochemistry, molecular weight, physical state, and melting point data during the submission procedure. In general, the NCI prioritizes molecules that contribute novel structural elements to its compound collection including new heterocyclic frameworks, recognized privileged scaffolds, and compounds generated through computer-aided drug design strategies. When a series of closely related analogues are submitted, the NCI typically selects the representative expected to provide the most meaningful biological information. Conversely, compounds exhibiting unfavorable properties—such as high conformational flexibility, high instability, nondrug-like functional groups (e.g., nitroso, diazo, or imine moieties), or structural features known to interfere with assay reliability (PAINS)—are generally excluded from screening.^35^ Following these criteria, seven out of 10 synthesized compounds were selected for biological testing, and after the receipt of 10 mg samples of each compound, the NCI assigned the corresponding following NSC identification numbers: **10a**, NSC850139; **10b**, NSC850141; **10c**, NSC850143; **10e**, NSC850145; **10f**, NSC850140; **10g**, NSC850142; **10h**, NSC850144. Standard operating procedures adopted by the NCI for sample preparation, including requirements for concentration and volume, as well as solubilization and plating protocols, are described elsewhere.^36^

#### 4.2.2 NCI-60 HTS384 Screening Methodology: One-Dose and Five-Doses Assays

The selected compounds were evaluated by the National Cancer Institute (NCI) using the updated HTS384 screening platform, a fully automated, high-throughput assay designed to assess the antiproliferative activity of small molecules across a panel of 60 human cancer cell lines (NCI-60). This platform represents an advancement over the former 96-well assay, as it is based on a 384-well plate format that requires lower cell numbers, allows extended compound exposure, and employs a CellTiter-Glo luminescence-based end point. These features enable more accurate determination of compound potency and tumor-type selectivity. A detailed description of the new developed assay design and experimental workflow is detailed in refs 32–34 .

Compounds **10a** (NSC850139), **10b** (NSC850141), **10c** (NSC850143), **10e** (NSC850145), **10f** (NSC850140), **10g** (NSC850142), and **10h** (NSC850144) were initially evaluated using a single-dose screening protocol at a concentration of 10^–5^ M against the complete NCI-60 human tumor cell line panel. This panel comprises 60 cell lines grouped into nine disease-oriented subpanels, representing major human cancer types, including leukemia, nonsmall-cell lung cancer (NSCLC), colon, central nervous system (CNS), melanoma, ovarian, renal, and breast cancers.^5,6^ The purpose of this preliminary assay is to determine the percentage of cell growth (*G*%) in comparison to untreated cells (*G*% = 100) after treatment by each compound. The resulting data are displayed as a one-dose profile reporting *G*% values across all 60 cell lines. Only compounds meeting predefined NCI threshold criteria with significant growth inhibition in this initial screen were advanced to the subsequent five-dose assay. Further details on the standardized screening procedures and evaluation criteria are available elsewhere.^32–34,36^

The most active compounds **10a** (NSC850139), **10b** (NSC850141), **10c** (NSC850143), **10f** (NSC850140), **10g** (NSC850142), and **10h** (NSC850144) were submitted to a multiple-dose screen using five different concentrations (ranging from 10^–8^ to 10^–4^ M). The dose–response curves obtained from this assay permitted the extrapolation of the GI_50_ (the molar concentration of the compound that inhibits 50% of cell growth), TGI (the molar concentration of the compound leading to total inhibition of cell growth), and LC_50_ (the molar concentration of the compound that induces 50% cell death) values of the selected compounds against each cancer cell line. For each of the mentioned parameters, both dose–response curves and bar graphs were furnished, providing an average activity parameter over all cell lines (for further experimental details about the standardized assay procedures, see refs 32–34 and 36).

### 4.3 In Silico Studies

#### 4.3.1 Ligand Preparation

Ligand preparation for in silico simulations was performed using the LigPrep module implemented in the Maestro graphical interface (Release 2017-1; Schrödinger LLC, New York, NY, USA).^25^ This tool was used to generate high-quality 3D structures suitable for structure-based virtual screening workflows. For each compound, exhaustive enumeration of tautomeric and ionization states, ring conformations, and stereoisomers was carried out based on the input structures, while preserving predefined chiral centers. Unwanted species, such as water molecules and counterions, were removed during the preparation process. Ionization states were calculated by using the Epik method under default settings within a physiological pH range of 7.0 ± 0.4. Subsequently, all generated ligand structures were subjected to energy minimization using the optimized potentials for liquid simulations (OPLS 2005) force field.^43^ The resulting low-energy conformations were then used as inputs for the subsequent virtual screening procedures.

#### 4.3.2 Protein Preparation

The X-ray crystal structures of the molecular targets investigated in this study were retrieved from the Protein Data Bank (PDB) in.pdb format.^44,45^ The structures used were PI3Kα (PDB ID: 8EXL),^37^ PI3Kγ (PDB ID: 3L08),^38^ Casein Kinase 2 (CK2, PDB ID: 3NGA),^39^ PIM1 kinase (PDB ID: 5O11),^40^ Mer kinase (PDB ID: 3TCP),^41^ and PARP1 (PDB ID: 7KK4).^42^

As the raw.pdb files are not directly suitable for molecular modeling due to the presence of crystallographic artifacts (e.g., water molecules, buffer components, metal ions, and cofactors), all protein structures were processed using the Protein Preparation Wizard implemented in the Schrödinger Maestro Suite, applying default settings.^43,46,47^ Briefly, each protein structure was imported into Maestro and preprocessed by assigning bond orders, including hetero groups, removing all water molecules, and determining protonation states of heteroatoms using the Epik tool at a physiologically relevant pH (7.0 ± 0.4). Disulfide bonds were generated, and missing side chains or loops located near the binding site were reconstructed by using the Prime module.

Subsequently, multimeric protein assemblies were simplified by removing redundant subunits. Finally, hydrogen-bond networks were optimized, and the protein structures were subjected to restrained energy minimization using the OPLS 2005 force field, with a convergence criterion defined by a maximum RMSD of 0.3 Å for atomic displacement.^43^ The resulting prepared protein structures were then used for subsequent molecular docking studies.

#### 4.3.3 Structure-Based Studies: Induced Fit Docking (IFD) Simulations

Induced fit docking (IFD) simulations were carried out on the top-ranked targets identified during the virtual screening workflow using the Schrödinger Induced Fit Docking tool, which is recognized for its ability to account for the flexibility of both ligands and protein receptors. The receptor models, previously prepared and minimized using the Protein Preparation Wizard, were employed as input.

The IFD scoring function, defined as IFD score = 1.0 × Glide GScore +0.05 × Prime Energy, combines the protein–ligand interaction energy with the overall system energy and was used to rank the predicted binding poses under default parameters.^48,49^ The docking protocol was validated by successfully redocking the cocrystallized ligands into their respective binding sites, achieving a root-mean-square deviation (RMSD) below 0.50 Å, confirming the reliability of the procedure.

#### 4.3.4 MM-GBSA Analysis

Molecular mechanics with generalized Born and surface area (MM-GBSA) calculations were performed using the Prime module (Release 2017-1; Schrödinger LLC, New York, NY, USA) to estimate the relative binding free energies of selected protein–ligand complexes. The analysis was conducted on the top-ranked derivatives identified from induced fit docking (IFD) studies using the corresponding best-ranked docked complexes as input structures.

Binding free energies were calculated by employing a single-trajectory approach, incorporating molecular mechanics energies and solvation effects. The VSGB solvation model was applied (water as the solvent) in combination with the OPLS-2005 force field. The total binding energy was decomposed into individual energetic contributions, including van der Waals (vdW), Coulomb (electrostatic), lipophilic, hydrogen-bonding, and polar solvation (GB) terms, allowing qualitative comparison with the respective cocrystallized inhibitors.

#### 4.3.5 Molecular Dynamics (MD) Simulations

Molecular dynamics (MD) simulations were performed to investigate the dynamic stability of the selected protein–ligand complexes in an explicit solvent environment. Simulations were carried out using the Desmond MD engine as implemented in Schrödinger Maestro (Release 2023-4; version 13.8.155, MMshare 6.4.195)^50^ on Linux x86_64 systems. Calculations were executed on a high-performance workstation equipped with an Intel Xeon CPU and an NVIDIA GPU under Ubuntu 22.04 LTS (64-bit).

Initial coordinates for each complex were derived from the corresponding docking poses and processed by using the System Builder module. Each system was solvated in an orthorhombic simulation box filled with SPC water molecules by applying a 10 Å buffer between the solute and the box boundaries. Appropriate counterions were introduced to ensure overall charge neutrality.

All simulations were performed by using the OPLS4 force field. Following energy minimization and a multistep relaxation protocol provided by Desmond, production runs were conducted for 100 ns under periodic boundary conditions in the NPT ensemble. Temperature was maintained at 300 K, and pressure was controlled at 1.01325 bar. Approximately 1000 trajectory frames were recorded for subsequent analysis.

Postsimulation analyses were performed using the simulation interaction diagram and trajectory analysis tools. Stability of the systems was assessed by monitoring the total and potential energy, temperature, pressure, and volume throughout the simulations. Structural stability was evaluated through root-mean-square deviation (RMSD) of protein backbone atoms and ligand heavy atoms, while residue-level flexibility was analyzed via root-mean-square fluctuation (RMSF). Protein–ligand contact persistence and ligand torsional behavior were also examined to characterize interaction stability during the simulation time.

#### 4.3.6 Density Functional Theory (DFT) Analysis

Density functional theory (DFT) calculations were performed using the Jaguar module implemented in Schrödinger Maestro.^51^ Single-point energy calculations were carried out at the M06-2X hybrid meta-GGA functional level in combination with the 6-31G** basis set, employing a nonrelativistic Hamiltonian. The M06-2X functional was selected due to its reliable performance in describing noncovalent interactions, thermochemical properties, and frontier orbital energies of organic systems, particularly for heteroaromatic and π-conjugated compounds. The 6-31G** basis set was chosen as a balanced split-valence polarized basis set, providing an adequate description of valence electron distribution and hydrogen-bonding contributions while maintaining reasonable computational cost for medium-sized drug-like molecules.

All calculations were performed in the gas phase without implicit solvation. The SCF spin treatment was set to automatic (restricted formalism for closed-shell systems). SCF convergence was achieved using the DIIS algorithm, with an energy convergence threshold of 5 × 10^–5^ Hartree and an RMS density matrix convergence threshold of 5 × 10^–6^. The integration grid density was set to medium. Frontier molecular orbital energies (HOMO and LUMO) were extracted from the converged wave functions. Molecular electrostatic potential (MEP) surfaces were generated by mapping the electrostatic potential onto the electron density isosurface.

Global reactivity descriptors were calculated according to Koopmans’ approximation using the DFT-derived orbital energies: Δ*E* = *E*_LUMO_ – *E*_HOMO_; μ = (*E*_HOMO_ + *E*_LUMO_)/2; η = (*E*_LUMO_ – *E*_HOMO_)/2; *S* = 1/(2η); ω = μ^2^/(2η), where μ is the chemical potential, η is the chemical hardness, *S* is the global softness, and ω is the electrophilicity index. All energies are reported in Hartree.

#### 4.3.7 Physicochemical, Drug-Likeness and ADME-Tox Property Prediction

ADME properties of the selected compounds were predicted using the QikProp module (Release 2017-1; Schrödinger LLC, New York, NY, USA), a fast and reliable tool available within the Schrödinger Maestro Suite (Maestro Version 11.1.012, Release 2017-1). QikProp calculates physically meaningful descriptors and pharmacologically relevant properties for individual molecules or compound libraries. Input structures, provided as .sdf files, were first prepared and energy-minimized using the LigPrep workflow (see previous section) before being processed in QikProp under normal settings.

Additional ADME parameters were obtained using the SwissADME web servers by uploading the SMILES representation of each compound. This combined approach enabled a comprehensive evaluation of the absorption, distribution, metabolism, and excretion profiles of the investigated molecules.^27^ Toxicity assessment was performed with a ProTox 3.0 Web server, by uploading the SMILES representation of each compound.^28–30^

## Supplementary Material





## Notes

Table S1**:** QikProp output matrix for compounds **7a**–**e** and **10a**–**j**; Table S2**:** SwissADME output matrix for compounds **7a**–**e** and **10a**–**j**; Table S3**:** ProTox 3.0 output matrix for compounds **10a**–**j** (XLSX)
Table S4**:** G% values for compounds **10a**–**c**,**e**–**h** against the NCI-60 panel (one dose assay); Table S5**:** GI_50_ values for compounds **10a**–**c**,**f**–**h** against the NCI-60 panel (five-dose assay); Table S6**:** TGI values for compounds **10a**–**c**,**f**–**h**against the NCI-60 panel (five-dose assay); Table S7**:** LC_50_ values for compounds **10a**–**c**,**f**–**h** against the NCI-60 panel (five-dose assay); Table S8**:** Induced Fit Docking results (IFD, Prime Energy and docking scores) for compounds **10a**–**c**,**f**–**h**; Figure S1**:** toxicity radar chart predicted by ProTox-3.0 web server for compounds **10a**–**j**; Figure S2**:** Predicted binding profiles of compounds **10a**–**j** toward a panel of key off-targets associated with adverse drug effects, as evaluated using the ProTox-3.0 web server;Figures S3–S24: ^1^HNMR and ^13^CNMR spectra of compounds **10a**–**j**; Figure S25**:** RMSD and 2D lig-interaction map analysis from MD simulations of cocrystallized reference ligands (PDF)

## Abbreviations

ADME: absorption, distribution, metabolism, and excretion
CK2: casein kinase 2
CNS: central nervous system
CSI: cytostatic selectivity index
DFT: density functional theory
FMO: frontier molecular orbital
GI50: concentration required to inhibit cell growth by 50%
HBA: hydrogen bond acceptor
HBD: hydrogen bond donor
HOA%: human oral absorption
HOMO: highest occupied molecular orbital
HTS: high-throughput screening
IFD: induced fit docking
LC50: lethal concentration required to kill 50% of cells
LRoF: Lipinski’s rule of five
LUMO: lowest unoccupied molecular orbital
MD: molecular dynamics
MDCK: Madin–Darby canine kidney cells
MEP: molecular electrostatic potential
MM-GBSA: molecular mechanics generalized Born surface area
MTC: medullary thyroid carcinoma
NCI: National Cancer Institute
NSCLC: nonsmall-cell lung cancer
PAINS: pan-assay interference compounds
PARP1: poly­(ADP-ribose) polymerase 1
PDB: Protein Data Bank
PI3K: phosphoinositide 3-kinase
P-gp: *P*-glycoprotein
RMSD: root-mean-square deviation
SASA: solvent-accessible surface area
TGI: total growth inhibition
TNBC: triple-negative breast cancer
