# Supplementary material for: Imidazole-Functionalized Thieno[3,2‑c]quinolines as Promising Antiproliferative Agents: Design, Synthesis, NCI-60 Screening, and Computational Analysis
Source: ACS Omega. 2026 May 7;11(19):29118–35. doi: 10.1021/acsomega.6c02681 (PMC13191679; doi:10.1021/acsomega.6c02681)
Supplement: Supplementary file 2 [file ao6c02681_si_002.pdf]

Supporting Material

Imidazole-Functionalized Thieno[3,2-*c*]quinolines as Promising Antiproliferative Agents: Design, Synthesis, NCI-60 Screening and Computational Analysis

AUTHOR NAMES: *Gabriele La Monica[a], Alessia Bono[a,b], Federica Alamia[a], Dennis Tocco[a], Antonino Lauria[a], Annamaria Martorana[a,]**

*Corresponding author: annamaria.martorana@unipa.it

AUTHOR ADDRESS:

[a]Dipartimento di Scienze e Tecnologie Biologiche Chimiche e Farmaceutiche "STEBICEF", University of Palermo, Viale delle Scienze– Ed. 17, 90128 Palermo, Italy

[b] Fondazione Umberto Veronesi (FUV), via Solferino 19, 20121 Milano, Italy.

Table of contents of Supporting Material

Supporting Tables:

- **Table S1:** QikProp output matrix for compounds **7a–e** and **10a–j** (provided as a separate .xlsx file).
- **Table S2:** SwissADME output matrix for compounds **7a–e** and **10a–j** (provided as a separate .xlsx file).
- **Table S3:** Pro-tox 3.0 output matrix for compounds **10a–j** (provided as a separate .xlsx file).
- **Table S4:** G% values for compounds **10a–c,e–h** against the NCI60 panel (one dose assay).
- **Table S5:** GI_{50} values for compounds **10a–c,f–h** against the NCI60 panel (five-dose assay).
- **Table S6:** TGI values for compounds **10a–c,f–h** against the NCI60 panel (five-dose assay).
- **Table S7:** LC_{50} values for compounds **10a–c,f–h** against the NCI60 panel (five-dose assay).
- **Table S8:** Induced Fit Docking results (IFD, Prime Energy and docking scores) for compounds **10a–c,f–h**.

Supporting Figures

- **Figure S1:** toxicity radar chart predicted by ProTox 3.0 web server for compounds **10a–j**.
- **Figure S2:** Predicted binding profiles of compounds **10a–j** toward a panel of key off-targets associated with adverse drug effects, as evaluated using the ProTox-3.0 web server.
- **Figures S3–24:** ^{1}HNMR and ^{13}CNMR spectra of compounds **10a–j**
  - *Figures S3-4*: ^{1}HNMR and ^{13}CNMR spectra of compound **10a** (d_6-DMSO).
  - *Figures S5-6*: ^{1}HNMR and ^{13}CNMR spectra of compound **10b** (d_6-DMSO).
  - *Figures S7-8*: ^{1}HNMR and ^{13}CNMR spectra of compound **10c** (d_6-DMSO).
  - *Figures S9-10*: ^{1}HNMR and ^{13}CNMR spectra of **10d** (d_6-DMSO).
  - *Figures S11-12:* ^{1}HNMR and ^{13}CNMR spectra of **10e** (d_6-DMSO).
  - *Figures S13-14*: ^{1}HNMR and ^{13}CNMR spectra of **10f** (d_6-DMSO).
  - *Figures S15-16*: ^{1}HNMR and ^{13}CNMR spectra of **10g** (d_6-DMSO).
  - *Figures S17-18*: ^{1}HNMR and ^{13}CNMR spectra of **10h** (d_6-DMSO).
  - *Figures S19-20*: ^{1}HNMR and ^{13}CNMR spectra of **10i** (d_6-DMSO).
  - *Figures S21-22*: ^{1}HNMR and ^{13}CNMR spectra of **10j** (d_6-DMSO).
  - *Figures S23:* Stacked ^{1}HNMR spectra of compounds **10a–j**.
  - *Figures S24*: Detailed ^{13}C NMR assignment and 2D NMR characterization of representative compound **10c.**
- **Figure S25:** RMSD and 2D lig-interaction map analysis from MD simulations of co-crystallized reference ligands.

Supporting Tables

Table S1-3: provided as separate .xlsx files.

Table S4: G% values for compounds **10a–c,e–h** against the NCI60 panel (one dose assay).

Growth inhibition percentage values (G%) provided by the National Cancer Institute for compounds **10a–c,e–h** tested against the full NCI60 panel.

Panel	Cell Line	Compounds G% (NSC code)						
		10a (850139)	10b (850141)	10c (850143)	10e (850145)	10f (850140)	10g (850142)	10h (850144)
LEUKEMIA	CCRF-CEM	-94.05	-94.99	-95.25	92.03	-95.83	-95.2	-95.13
	HL-60(TB)	-96.71	-94.6	-94.59	92.2	-96.11	-94.75	-95.06
	K-562	-25.79	54.38	-97.61	93.27	-82.67	50.04	48.36
	MOLT-4	-66.77	-87.42	-96.75	82.71	-97.3	-96.81	-96.78
	RPMI-8226	-95.72	-95.49	-98.03	53.63	-98.29	-97.91	-98.13
	SR	N.A.	N.A.	N.A.	N.A.	N.A.	N.A.	N.A.
NSCLC	A549/ATCC	44.86	-74.43	-91.71	85.22	-91.6	23.53	-91.89
	EKVX	28.87	-38.23	-96.71	73.89	-61.06	-22.3	-96.87
	HOP-62	-70.76	-97.19	-97.82	40.34	-98.07	-97.29	-97.8
	HOP-92	-47.46	-89.84	-98.6	50.66	-93.97	-68.44	-98.67
	NCI-H226	-77.39	-77.11	-98.52	16.96	-86.46	-14.93	-79.83
	NCI-H23	53.6	-57.2	-97.37	83.08	-78.46	-42.11	-97.1
	NCI-H322M	27.6	-86.6	-97.82	75.72	-94.55	-1.91	-68.2
	NCI-H460	8.54	-84.65	-86.59	91.87	-86.87	-87.21	-86.93
	NCI-H522	0.82	-91.78	-98.71	93.6	-98.65	-95.08	-98.77
COLON CANCER	COLO-205	-38.7	-55.11	-95.93	79.83	-74.39	10.27	-63.52
	HCC-2998	32.38	-52.14	-97.59	77.28	-97.21	-82.6	-97.72
	HCT-116	-53.63	-77.36	-92.16	90	-92.68	-91.26	-92.5
	HCT-15	29.8	-67.13	-93.28	93.33	-93.12	-92.66	-93.42
	HT29	-87.33	-94.16	-97.18	92.23	-97.54	-96.68	-97.14
	KM12	-41.47	-72.95	-93.49	83.43	-93.86	-93.26	-91.61
	SW-620	-36.96	-58.05	-97.24	82.85	-97.29	-91.09	-97.35
CNS CANCER	SF-268	15.93	-85.19	-96.99	68.37	-92.68	-53.19	-97.29
	SF-295	67.97	-92.58	-98.18	104.65	-5.78	23.28	-83.42
	SF-539	-94.58	-96.84	-96.77	83.64	-96.68	-96.67	-96.79
	SNB-19	19.12	-70.38	-96.4	71.44	-69.17	-41.78	-89.98
	SNB-75	-81.48	-98.09	-98.58	-9.4	-95.28	-72.65	-98.58
	U251	-32.02	-90.02	-96.18	83.35	-96.12	-87.82	-96.37

MELANOMA	LOX_IMVI	24.21	-70.58	-96.99	108.96	-96.73	-81.98	-96.93
	MALME-3M	-97.28	-97.02	-98.13	50.51	-98.18	-98.24	-98.34
	M14	-10.59	-27.83	-96.91	105.9	-95.99	-51.03	-96.29
	MDA-MB-435	27.17	-23.89	-96.56	94.44	-95.79	-80.82	-97.02
	SK-MEL-2	-80.83	-97.65	-98.05	46.14	-97.92	-97.63	-98.1
	SK-MEL-28	-81.08	-94.71	-98.41	66.04	-97.95	-90.53	-98.07
	SK-MEL-5	-39.5	-86.29	-96.63	64.83	-97.07	-86.26	-94.35
	UACC-257	-77.71	-96.53	-98.16	72.63	-98.32	-97.86	-98.31
	UACC-62	-74.57	-93.35	-97.38	97.93	-97.31	-96.16	-97.54
OVARIAN CANCER	IGROV1	-2.35	-76.43	-99.05	43.4	-94.26	1.57	-98.81
	OVCAR-3	-5.35	-94.44	-98.96	63.03	-98.83	-96.82	-98.96
	OVCAR-4	-95.13	-92.82	-98.48	41.54	-97.78	-89.17	-98.38
	OVCAR-5	N.A.	N.A.	N.A.	N.A.	N.A.	N.A.	N.A.
	OVCAR-8	-74.55	-93.58	-93.98	57.71	-94.37	-88.23	-94.4
	NCI/ADR-RES	59.48	17.43	-59.29	88.99	-16.44	15.56	-2.18
	SK-OV-3	27.77	-95.24	-97.19	81.62	-97.18	-83.94	-97.24
RENAL CANCER	786-0	-91.6	-97.57	-98.32	58.11	-98.33	-97.1	-98.28
	A498	-73.68	-96.29	-97.32	66.13	-97.21	-92.36	-97.34
	ACHN	-59.9	-71.05	-96.37	67.42	-90.32	-15.13	-92.16
	CAKI-1	-95.8	-97.86	-98.43	86.65	-98.37	-97.74	-98.65
	RXF-393	-97.68	-97.52	-98.11	11.53	-98.24	-97.36	-97.95
	SN12C	9.62	-96.15	-96.38	63.23	-96.55	-94.58	-96.39
	TK-10	-18.6	-88.29	-99.06	91.06	-98.58	-97.81	-98.7
	UO-31	N.A.	N.A.	N.A.	N.A.	N.A.	N.A.	N.A.
PROSTATE CANCER	PC-3	-15.5	-90.95	-97.26	61.89	-97.05	-75.6	-97.6
	DU-145	53.62	-14.25	-94.33	87.63	-82.58	-28.45	-84.09
BREAST CANCER	MCF7	-56.15	-90.72	-93.48	72.61	-94.51	-85.62	-93.81
	MDA-MB-231/ATCC	N.A.	N.A.	N.A.	N.A.	N.A.	N.A.	N.A.
	HS-578T	14.12	-98.66	-99.08	51.94	-77.45	-59.96	-99.08
	BT-549	-24.27	-97.31	-97.12	73.36	-97.51	-96.36	-97.29
	T-47D	N.A.	N.A.	N.A.	N.A.	N.A.	N.A.	N.A.
	MDA-MB-468	-90.71	-91.76	-98.99	23.16	-99.56	-98.86	-99.49
Mean G% (compound)		-31.97	-77.75	-96.08	71.36	-90.22	-68.45	-90.26
Delta		65.71	20.91	3.00	80.76	9.34	30.41	9.23
Range		165.65	153.04	39.79	118.36	93.78	148.90	147.85

N.A.: not available.

Table S5: GI_{50} values for compounds **10a–c,f–h** against the NCI60 panel (five-dose assay).

GI_{50} (the molar concentration of the compound that inhibits 50% of cell growth) values provided by the National Cancer Institute for compounds **10a–c,f–h** tested against the full NCI60 panel. Mean values are provided both per compound (columns), representing the average GI_{50} across all tested cell lines, and per cell line (rows), corresponding to the average GI_{50} of the tested compounds against each cell line.

Panel	Cell Line	Compounds $LOG_{10}(GI_{50})$ (NSC code)						
		10a (850139)	10b (850141)	10c (850143)	10f (850140)	10g (850142)	10h (850144)	Mean GI_{50} (cell line)
LEUKEMIA	CCRF-CEM	-6.2	-5.86	-6.19	-5.78	-6.7	-6.45	-6.20
	HL-60(TB)	-5.85	-5.9	-6.61	-5.75	-6.65	-6.67	-6.24
	K-562	-5.75	-5.74	-5.93	-5.72	-6.13	-5.82	-5.85
	MOLT-4	-5.89	-5.83	-6.17	-5.78	-6.63	-6.39	-6.12
	RPMI-8226	-5.88	-5.71	-5.97	-5.69	-6.53	-6.11	-5.98
	SR	-6.26	-5.91	-6.79	-5.99	-6.68	-6.76	-6.40
NSCLC	A549/ATCC	-4.51	-5.54	-5.87	-5.75	-5.55	-5.54	-5.46
	EKVX	-6.16	-6.63	-6.39	-6.32	-6.71	-6.39	-6.43
	HOP-62	N.A.	N.A.	N.A.	N.A.	N.A.	N.A.	N.A.
	HOP-92	-5.96	-5.89	-6.7	-5.86	-6	-5.98	-6.07
	NCI-H226	-5.56	-6.26	-6	-5.91	-6.09	-5.99	-5.97
	NCI-H23	-4.88	-5.79	-5.85	-5.77	-5.78	-5.82	-5.65
	NCI-H322M	-5.38	-5.68	-5.8	-5.66	-5.66	-5.64	-5.64
	NCI-H460	-5.17	-5.57	-6.29	-5.72	-5.83	-5.81	-5.73
	NCI-H522	-5.38	-5.53	-5.31	-5.61	-5.54	-5.58	-5.49
COLON CANCER	COLO-205	-5.29	-5.67	-5.76	-5.62	-5.52	-5.6	-5.58
	HCC-2998	-5.08	-5.56	-5.75	-5.7	-5.89	-5.72	-5.62
	HCT-116	-5.57	-5.71	-6.09	-5.75	-5.93	-5.81	-5.81
	HCT-15	-5.47	-5.7	-5.81	-5.75	-6.28	-6.02	-5.84
	HT29	-5.79	-4.65	-5.84	-5.76	-5.78	-5.81	-5.61
	KM12	-4.92	-5.37	-6.4	-5.64	-6.11	-5.72	-5.69
	SW-620	-5.77	-5.46	-5.85	-5.77	-5.83	-5.78	-5.74
CNS CANCER	SF-268	-4.58	-5.8	-5.93	-5.63	-5.79	-5.7	-5.57
	SF-295	N.A.	N.A.	N.A.	N.A.	N.A.	N.A.	N.A.
	SF-539	-5.78	-5.78	-6.33	-5.81	-5.82	-5.9	-5.90
	SNB-19	-5.17	-5.58	-5.85	-5.72	-5.77	-5.74	-5.64
	SNB-75	-5.46	-5.71	-6.49	-5.74	-5.7	-5.69	-5.80

	**U251**	N.A.	N.A.	N.A.	N.A.	N.A.	N.A.	N.A.
**MELANOMA**	**LOX_IMVI**	-5.64	-5.79	-6.46	-5.78	-6.28	-6.45	-6.07
	**MALME-3M**	-5.7	-5.95	-5.91	-5.77	-5.94	-6.27	-5.92
	**M14**	-5.4	-5.56	-5.85	-5.65	-5.7	-5.71	-5.65
	**MDA-MB-435**	N.A.	N.A.	N.A.	N.A.	N.A.	N.A.	N.A.
	**SK-MEL-2**	-6.36	-6.07	-6.7	-5.86	-6.7	-6.46	-6.36
	**SK-MEL-28**	-5.78	-5.83	-6.01	-5.8	-5.82	-6.18	-5.90
	**SK-MEL-5**	-5.67	-5.88	-5.84	-5.83	-5.82	-6	-5.84
	**UACC-257**	-5.67	-5.74	-6.01	-5.72	-5.89	-6.3	-5.89
	**UACC-62**	-5.63	-5.67	-6.34	-5.71	-6.23	-5.92	-5.92
**OVARIAN CANCER**	**IGROV1**	-5.73	-5.91	-5.98	-5.82	-5.88	-5.87	-5.87
	**OVCAR-3**	-5.75	-6.16	-6.51	-5.85	-5.96	-6.02	-6.04
	**OVCAR-4**	-5.95	-5.95	-6.69	-5.84	-5.92	-5.95	-6.05
	**OVCAR-5**	-4.4	-5.27	-5.78	-5.32	-5.66	-5.27	-5.28
	**OVCAR-8**	-5.67	-5.85	-5.9	-5.8	-5.95	-5.89	-5.84
	**NCI/ADR-RES**	>-4.00	-5.47	-5.73	-5.42	-5.49	>-4.00	-5.53
	**SK-OV-3**	-5.27	-5.82	-6.7	-5.78	-5.83	-5.83	-5.87
**RENAL CANCER**	**786-0**	-5.81	-5.98	-5.92	-5.89	-5.94	-5.92	-5.91
	**A498**	-5.56	-5.84	-5.99	-5.78	-5.83	-5.88	-5.81
	**ACHN**	-5.83	-5.83	-5.84	-5.83	-5.85	-5.81	-5.83
	**CAKI-1**	-5.85	-5.78	-6.3	-5.81	-5.9	-5.82	-5.91
	**RXF-393**	N.A.	N.A.	N.A.	N.A.	N.A.	N.A.	N.A.
	**SN12C**	-5.92	-6.79	-5.98	-6.39	-6.97	-6.31	-6.39
	**TK-10**	-5.66	-5.79	-6.43	-5.78	-5.83	-5.96	-5.91
	**UO-31**	N.A.	N.A.	N.A.	N.A.	N.A.	N.A.	N.A.
**PROSTATE CANCER**	**PC-3**	-5.35	-5.78	-6.48	-5.76	-5.85	-5.83	-5.84
	**DU-145**	-5.39	-5.31	-5.96	-5.74	-5.75	-5.8	-5.66
**BREAST CANCER**	**MCF7**	-5.62	-5.84	-6.38	-5.77	-6.22	-5.98	-5.97
	**MDA-MB-231/ATCC**	-5.65	-5.86	-6.55	-5.79	-6.19	-6.19	-6.04
	**HS-578T**	-4.36	-5.83	-6.28	-5.68	-5.67	-5.71	-5.59
	**BT-549**	N.A.	N.A.	N.A.	N.A.	N.A.	N.A.	N.A.
	**T-47D**	-5.54	-5.81	-5.87	-5.78	-5.81	-5.76	-5.76
	**MDA-MB-468**	N.A.	N.A.	N.A.	N.A.	N.A.	N.A.	N.A.
**Mean GI_{50} (compound)**		-5.15	-5.52	-6.05	-5.74	-5.9	-5.48	

N.A.: not available.

Table S6: TGI values for compounds **10a–c,f–h** against the NCI60 panel (five-dose assay).

TGI (the molar concentration of the compound leading to total inhibition of cell growth) values provided by the National Cancer Institute for compounds **10a–c,f–h** tested against the full NCI60 panel. Mean values are provided both per compound (columns), representing the average TGI across all tested cell lines, and per cell line (rows), corresponding to the average TGI of the tested compounds against each cell line.

Panel	Cell Line	Compounds LOG_{10}(TGI) (NSC code)						
		10a (850139)	10b (850141)	10c (850143)	10f (850140)	10g (850142)	10h (850144)	Mean TGI (cell line)
LEUKEMIA	CCRF-CEM	-5.69	-5.57	-5.74	-5.51	-6.45	-5.99	-5.83
	HL-60(TB)	-5.56	-5.6	-6.37	-5.5	-6.42	-6.44	-5.98
	K-562	-5.13	-5.48	-5.61	-5.47	-5.69	-5.54	-5.49
	MOLT-4	-5.55	-5.54	-5.73	-5.51	-6.31	-5.88	-5.75
	RPMI-8226	-5.48	-5.46	-5.64	-5.46	-6.22	-5.7	-5.66
	SR	-5.81	-5.61	-6.52	-5.65	-6.45	-6.5	-6.09
NSCLC	A549/ATCC	>-4.00	-4.94	-5.57	-5.49	-4.73	-4	-4.95
	EKVX	-5.36	-5.89	-5.82	-5.72	-5.86	-5.68	-5.72
	HOP-62	N.A.	N.A.	N.A.	N.A.	N.A.	N.A.	N.A.
	HOP-92	-5.4	-5.58	-6.2	-5.57	-5.66	-5.65	-5.68
	NCI-H226	-4.27	-5.56	-5.66	-5.15	-5.38	-4.15	-5.03
	NCI-H23	-4.03	-5.48	-5.56	-5.51	-5.49	-5.53	-5.27
	NCI-H322M	-4.39	-5.4	-5.53	-5.36	-5.28	-5.17	-5.19
	NCI-H460	-4.17	-5.15	-5.77	-5.47	-5.54	-5.5	-5.27
	NCI-H522	-5.09	-5.32	N.A.	-5.4	-5.33	-5.38	-5.30
COLON CANCER	COLO-205	-4.3	-5.44	-5.5	-5.37	-5.04	-5.16	-5.14
	HCC-2998	>-4.00	-5.17	-5.5	-5.46	-5.56	-5.46	-5.43
	HCT-116	-4.84	-5.35	-5.68	-5.48	-5.61	-5.52	-5.41
	HCT-15	-4.62	-5.22	-5.53	-5.49	-5.72	-5.64	-5.37
	HT29	-5.46	>-4.00	-5.55	-5.5	-5.5	-5.52	-5.51
	KM12	-4.27	-4.71	-5.76	-5.29	-5.68	-5.26	-5.16
	SW-620	-4.9	-4.91	-5.56	-5.51	-5.53	-5.51	-5.32
CNS CANCER	SF-268	>-4.00	-5.46	-5.61	-5.25	-5.37	-5	-5.34
	SF-295	N.A.	N.A.	N.A.	N.A.	N.A.	N.A.	N.A.
	SF-539	-5.5	-5.52	-5.78	-5.54	-5.54	-5.59	-5.58
	SNB-19	>-4.00	-4.9	-5.56	-5.36	-5.19	-5.26	-5.25
	SNB-75	-5.01	-5.41	-6.08	-5.42	-5.46	-5.38	-5.46
	U251	N.A.	N.A.	N.A.	N.A.	N.A.	N.A.	N.A.
MELANOMA	LOX_IMVI	-5.29	-5.52	-5.92	-5.52	-5.76	-5.88	-5.65
	MALME-3M	-5.29	-5.62	-5.6	-5.51	-5.62	-5.77	-5.57

	**M14**	-4.6	-5.24	-5.56	-5.43	-5.46	-5.46	-5.29
	**MDA-MB-435**	-5.74	N.A.	N.A.	N.A.	N.A.	N.A.	-5.74
	**SK-MEL-2**	-5.46	-5.67	-6.45	-5.57	-6.2	-5.92	-5.88
	**SK-MEL-28**	-5.09	-5.54	-5.67	-5.53	-5.54	-5.69	-5.51
	**SK-MEL-5**	-5.41	-5.54	-5.56	-5.55	-5.53	-5.59	-5.53
	**UACC-257**	N.A.	-5.46	-5.67	-5.48	-5.59	-5.78	-5.60
	**UACC-62**	-5.24	-5.41	-5.85	-5.47	-5.75	-5.59	-5.55
**OVARIAN CANCER**	**IGROV1**	-4.6	-5.54	-5.65	-5.52	-5.46	-4	-5.13
	**OVCAR-3**	-5.1	-5.44	-5.87	-5.57	-5.6	-5.4	-5.50
	**OVCAR-4**	-5.62	-5.53	-6.37	-5.56	-5.6	-5.62	-5.72
	**OVCAR-5**	>-4.00	-4.47	-5.52	-4.3	N.A.	-4	-4.57
	**OVCAR-8**	-5.18	-5.38	-5.59	-5.53	-5.61	-5.55	-5.47
	**NCI/ADR-RES**	>-4.00	-4.87	-5.37	-4.73	>-4.00	-4	-4.74
	**SK-OV-3**	-4.56	-5.54	-6.31	-5.51	-5.53	-5.55	-5.50
**RENAL CANCER**	**786-0**	-5.42	-5.65	-5.61	-5.59	-5.59	-5.6	-5.58
	**A498**	-5.06	-5.53	-5.65	-5.51	-5.55	-5.46	-5.46
	**ACHN**	-5.45	-5.53	-5.56	-5.55	-5.55	-5.34	-5.50
	**CAKI-1**	-5.49	-5.28	-5.76	-5.54	-5.59	-5.48	-5.52
	**RXF-393**	N.A.	N.A.	N.A.	N.A.	N.A.	N.A.	N.A.
	**SN12C**	>-4.00	-4.84	-5.65	-5.65	-5.47	-4	-5.12
	**TK-10**	-5.27	-5.44	-5.85	-5.52	-5.54	-5.62	-5.54
	**UO-31**	N.A.	N.A.	N.A.	N.A.	N.A.	N.A.	N.A.
**PROSTATE CANCER**	**PC-3**	-4.6	-5.47	-6.02	-5.5	-5.54	-5.44	-5.43
	**DU-145**	>-4.00	-4.73	-5.63	-5.45	-5.41	-5.18	-5.28
**BREAST CANCER**	**MCF7**	-5.03	-5.53	-5.86	-5.51	-5.73	-5.64	-5.55
	**MDA-MB-231/ATCC**	-5.18	-5.5	-6.19	-5.52	-5.71	-5.68	-5.63
	**HS-578T**	>-4.00	-5.5	-5.75	-5.36	-5.42	-5.37	-5.48
	**BT-549**	N.A.	N.A.	N.A.	N.A.	N.A.	N.A.	N.A.
	**T-47D**	-5	-5.5	-5.58	-5.49	-5.48	-5.4	-5.41
	**MDA-MB-468**	N.A.	N.A.	N.A.	N.A.	N.A.	N.A.	N.A.
**Mean (compound)**		-4.51	-5.1	-5.68	-5.33	-5.3	-4.9	

N.A.: not available.

Table S7: LC_{50} values for compounds **10a–c,f–h** against the NCI60 panel (five-dose assay).

LC_{50} (the molar concentration of the compound that induces 50% cell death) values provided by the National Cancer Institute for compounds **10a–c,f–h** tested against the full NCI60 panel.

Panel	Cell Line	Compounds $LOG_{10}(LC_{50})$ (NSC code)					
		10a (850139)	10b (850141)	10c (850143)	10f (850140)	10g (850142)	10h (850144)
LEUKEMIA	CCRF-CEM	-5.26	-5.28	-5.36	-5.25	-6.19	-5.47
	HL-60(TB)	-5.27	-5.29	-6.12	-5.24	-6.2	-6.21
	K-562	-4.49	-5.22	-5.29	-5.23	-5.32	-5.25
	MOLT-4	-5.2	-5.25	-5.36	-5.25	-5.96	-5.43
	RPMI-8226	-5.09	-5.21	-5.32	-5.23	-5.77	-5.35
	SR	-5.4	-5.3	-6.24	-5.32	-6.21	-6.24
NSCLC	A549/ATCC	>-4.00	>-4.00	-5.27	-5.23	>-4.00	>-4.00
	EKVX	>-4.00	-5.36	-5.41	-5.24	>-4.00	N.A.
	HOP-62	N.A.	N.A.	N.A.	N.A.	N.A.	N.A.
	HOP-92	-4.53	-5.27	-5.62	-5.28	-5.33	-5.32
	NCI-H226	>-4.00	-5.03	-5.33	-4.36	>-4.00	>-4.00
	NCI-H23	>-4.00	-5.16	-5.28	-5.24	-5.19	-5.24
	NCI-H322M	>-4.00	-5.11	-5.26	-5.07	-4.05	>-4.00
	NCI-H460	>-4.00	-4.58	-5.35	-5.21	-5.24	-5.18
	NCI-H522	-4.38	-5.11	N.A.	-5.2	-5.12	-5.19
COLON CANCER	COLO-205	>-4.00	-5.2	-5.24	-5.13	-4.3	>-4.00
	HCC-2998	>-4.00	-4.48	-5.24	-5.23	-5.22	N.A.
	HCT-116	-4.1	>-4.00	-5.33	-5.21	-5.29	-5.24
	HCT-15	>-4.00	-4.53	-5.25	-5.23	-5.32	-5.27
	HT29	-5.13	>-4.00	-5.26	-5.23	-5.22	-5.23
	KM12	>-4.00	-4.23	>-4.00	-4.86	-5.32	>-4.00
	SW-620	-4.19	-4.39	-5.27	-5.25	-5.24	-5.25
CNS CANCER	SF-268	>-4.00	-5.11	-5.29	-4.72	-4.71	>-4.00
	SF-295	N.A.	N.A.	N.A.	N.A.	N.A.	N.A.
	SF-539	-5.23	-5.26	-5.38	-5.26	-5.27	-5.28
	SNB-19	>-4.00	-4.19	-5.27	-5	>-4.00	>-4.00
	SNB-75	>-4.00	-5.12	-5.55	-5.11	-5.21	N.A.
	U251	N.A.	N.A.	N.A.	N.A.	N.A.	N.A.
MELANOMA	LOX_IMVI	-4.85	-5.25	-5.45	-5.25	-5.37	-5.43
	MALME-3M	-4.76	-5.29	-5.3	-5.25	-5.31	-5.37
	M14	>-4.00	-4.71	-5.28	-5.21	-5.23	-5.2
	MDA-MB-435	-5.26	N.A.	N.A.	N.A.	N.A.	N.A.
	SK-MEL-2	-5.14	-5.31	-6.2	-5.28	-5.61	-5.45
	SK-MEL-28	>-4.00	-5.26	-5.33	-5.26	-5.25	-5.31
	SK-MEL-5	-5.15	-5.2	-5.27	-5.27	-5.24	-5.18

	**UACC-257**	N.A.	-5.18	-5.33	-5.24	-5.29	-5.39
	**UACC-62**	-4.72	-5.14	-5.42	-5.23	-5.37	-5.26
**OVARIAN CANCER**	**IGROV1**	>-4.00	-5.16	-5.32	-5.22	-5.03	N.A.
	**OVCAR-3**	>-4.00	-4.73	-5.43	-5.28	-5.24	>-4.00
	**OVCAR-4**	-5.29	-5.1	-6.04	-5.27	-5.28	-5.28
	**OVCAR-5**	>-4.00	>-4.00	-5.25	>-4.00	>-4.00	>-4.00
	**OVCAR-8**	-4.47	-4.81	-5.29	-5.25	-5.26	-5.21
	**NCI/ADR-RES**	>-4.00	>-4.00	-5	>-4.00	>-4.00	>-4.00
	**SK-OV-3**	>-4.00	-5.27	-5.82	-5.25	-5.23	-5.26
**RENAL CANCER**	**786-0**	-5.03	-5.32	-5.3	-5.29	-5.25	-5.28
	**A498**	-4.39	-5.22	-5.32	-5.24	-5.26	-5.04
	**ACHN**	-5.07	-5.23	-5.27	-5.27	-5.24	>-4.00
	**CAKI-1**	-5.14	-4.64	-5.37	-5.26	-5.28	-5.13
	**RXF-393**	N.A.	N.A.	N.A.	N.A.	N.A.	N.A.
	**SN12C**	>-4.00	-4.02	-5.32	-5.23	-4.55	>-4.00
	**TK-10**	-4.54	-5.09	-5.42	-5.25	-5.26	-5.28
	**UO-31**	N.A.	N.A.	N.A.	N.A.	N.A.	N.A.
**PROSTATE CANCER**	**PC-3**	>-4.00	-5.16	-5.5	-5.24	-5.22	N.A.
	**DU-145**	>-4.00	-4.29	-5.3	-5.16	-5.08	>-4.00
**BREAST CANCER**	**MCF7**	-4.26	-5.22	-5.42	-5.25	-5.32	-5.3
	**MDA-MB-231/ATCC**	-4.5	-5.15	-5.67	-5.25	-5.31	-5.26
	**HS-578T**	-4	-5.17	-5.37	-5.05	-5.17	-5.02
	**BT-549**	N.A.	N.A.	N.A.	N.A.	N.A.	N.A.
	**T-47D**	>-4.00	-5.18	-5.28	-5.21	-5.15	-5.05
	**MDA-MB-468**	N.A.	N.A.	N.A.	N.A.	N.A.	N.A.
**Mean (compound)**		-4.23	-4.66	-5.33	-4.95	-4.73	-4.51

N.A.: not available.

Table S8: Induced Fit Docking results (IFD, Prime Energy and docking scores) for compounds **10a–c,f–h**.

Complete Induced Fit Docking (IFD) results, including IFD scores, Docking scores, and Prime energy values obtained for compounds **10a–c,f–h,** evaluated in the five-dose NCI assay, against selected targets previously reported to be inhibited by thienoquinoline derivatives (PI3Ks, CK2, PIM1, Mer kinase and PARP1). For each target, the first series of compounds **7a–e** corresponding co-crystallized inhibitors and reference ligands are included as controls.

Target	Compound§	Docking Score	Prime Energy	IFD Score
PI3Kα (PDB id 8EXL)*	**10c** *	-12.152	-40568.2	-2040.65
	**10h**	-12.315	-40540.8	-2039.38
	**10b**	-11.298	-40559.7	-2039.37
	**10g**	-11.162	-40551.5	-2038.77
	**10f**	-11.503	-40543.8	-2038.72
	omipalisib **(CID 25167777)**	-10.103	-40558.1	-2038.1
	**7b**	-11.533	-40512.1	-2037.14
	**compound 1 (TQ PI3Kα inh.)**	-9.847	-40543.4	-2037.1
	**10a**	-10.050	-40533.9	-2036.83
	**7c**	-10.661	-40505.7	-2035.95
	dactolisib **(CID 11977753)**	-11.063	-40494.5	-2035.79
	**7e**	-11.038	-40493.7	-2035.72
	**7a**	-10.985	-40486.1	-2035.29
	taselisib **(cocryst. lig. 8EXL, CID 51001932)**	-9.526	-40506.2	-2034.85
	**7d**	-10.143	-40489.7	-2034.63
PI3Kγ (PDB id 3L08)	***omipalisib*** **(cocryst. lig. 3L08, CID 25167777)**	-11.925	-36429.2	-1833.48
	**10h**	-11.795	-36420	-1832.83
	dactolisib **(CID 11977753)**	-12.246	-36358.7	-1830.18
	**10f**	-9.275	-36395.4	-1829.07
	**10c**	-9.246	-36394.5	-1829.06
	**10g**	-8.277	-36411.4	-1828.88
	**10a**	-9.56	-36378.8	-1828.59
	**10b**	-8.264	-36400.7	-1828.39
	**7b**	-8.828	-36356.3	-1826.64
	**7a**	-9.279	-36346.3	-1826.59
	**7e**	-8.74	-36350.7	-1826.27
	**compound 2 (TQ PI3Kγ inh.)**	-7.296	-36376.3	-1826.11
	**7d**	-9.056	-36341	-1826.11
	**7c**	-6.958	-36363.3	-1825.12

**Casein Kinase 2 (PDB id 3NGA)***	**CX-4945 (cocryst. lig. 3NGA, CID 24748573)**	-11.884	-15525.4	-788.16
	**10c ***	-10.798	-15529.8	-787.38
	**10h**	-9.479	-15542.2	-786.62
	**10b**	-10.246	-15513	-785.99
	**10f**	-9.354	-15529.1	-785.84
	**10g**	-9.054	-15524.6	-785.32
	**7c**	-10.76	-15488.7	-785.2
	**10a**	-9.523	-15510.8	-785.15
	**compound 3 (TQ CK2 inh.)**	-10.085	-15454.3	-782.8
	**7e**	-9.276	-15470.1	-782.78
	**7a**	-9.282	-15469.9	-782.78
	**7b**	-9.814	-15445.7	-782.1
	**7d**	-8.652	-15445.9	-780.95
**PIM1 kinase (PDB id 5O11)***	**10h***	-9.955	-12234.3	-621.7
	**10c**	-10.728	-12207.3	-621.18
	**10f**	-9.156	-12232.5	-620.81
	**10g**	-9.3	-12225.9	-620.62
	**10b**	-8.753	-12224	-620.04
	**CX-4945 (cocryst. lig. 3NGA, CID 24748573)**	-9.04	-12197.6	-618.92
	**10a**	-8.854	-12193.9	-618.64
	**compound 3 (TQ CK2 inh.)**	-7.527	-12171.9	-616.12
	**7b**	-6.125	-12180.2	-615.13
	**7c**	-6.314	-12171.8	-614.9
	**7a**	-7.052	-12146.9	-614.4
	**7e**	-6.47	-12157.8	-614.36
	**7d**	-6.458	-12143.8	-613.65
**Mer kinase (PDB id 3TCP)**	**UNC569 (cocryst. lig. 3TCP, CID 53355503)**	-9.97	-11305.4	-575.24
	**10b**	-9.497	-11218.8	-570.53
	**10f**	-9.738	-11210.2	-570.28
	**compound 4 (TQ Mer inh.)**	-8.412	-11219.3	-569.38
	**10c**	-8.983	-11192.8	-568.71
	**10g**	-8.453	-11200.1	-568.49
	**7c**	-8.474	-11197	-568.33
	**10a**	-8.928	-11185.7	-568.3
	**10h**	-8.24	-11195.7	-568.05
	**7b**	-8.581	-11188.2	-567.99

	**7d**	-8.198	-11173.1	-566.85
	**7a**	-8.248	-11152.6	-565.88
	**7e**	-7.658	-11145.7	-564.94
**PARP1 (PDB id 7KK4)***	**10h***	-9.959	-14736.7	-746.82
	**compound 5 (TQ PARP1 inh.)**	-11.892	-14693.7	-746.57
	**10f**	-9.873	-14732.8	-746.54
	**10g**	-9.347	-14733.1	-746.03
	**compound 6 (TQ PARP1 inh.)**	-11.53	-14680.1	-745.53
	**10b**	-9.017	-14728	-745.51
	olaparib **(cocryst. lig. 7KK4, CID 23725625)**	-13.448	-14637.3	-745.31
	**10a**	-9.377	-14715.5	-745.24
	rucaparib **(CID 9931954)**	-10.629	-14681.3	-744.71
	**10c**	-8.333	-14715.2	-744.18
	talazoparib **(CID 135565082)**	-11.305	-14628.6	-742.74
	**7e**	-7.432	-14696.2	-742.24
	**7d**	-8.818	-14659.1	-741.77
	**7c**	-8.786	-14658.2	-741.7
	**7a**	-7.626	-14665.4	-740.89
	**7b**	-7.554	-14661.4	-740.62

(*) Targets selected for subsequent molecular dynamics (MD) simulations; for each of these targets, the best-ranked compound was chosen as the representative ligand for further MD analysis.
(§) Compounds of type 10 evaluated in the five-dose NCI assay are highlighted **in green**. Nitro precursors are shown **in red** [1]. Reference inhibitors are reported **in black**, including known inhibitors, co-crystallized ligands, and previously reported thienoquinoline derivatives active (compound 1 [2], compound 2 [3], compound 3 [4], compound 4 [5], compound 5 [6], compound 6 [7]) against the corresponding target.

Supporting Figures

Figure S1: toxicity radar chart predicted by ProTox 3.0 web server for compounds **10a–j.**

The blue area represents the predicted probability of activity for each toxicity endpoint for the investigated compounds, while the orange area corresponds to the average probability for known active compounds within each toxicity class. The analysis encompasses a wide range of toxicity endpoints, including organ toxicity, toxicological endpoints, nuclear receptor signalling pathways, stress response pathways, molecular initiating events (MIEs), and metabolism-related targets.

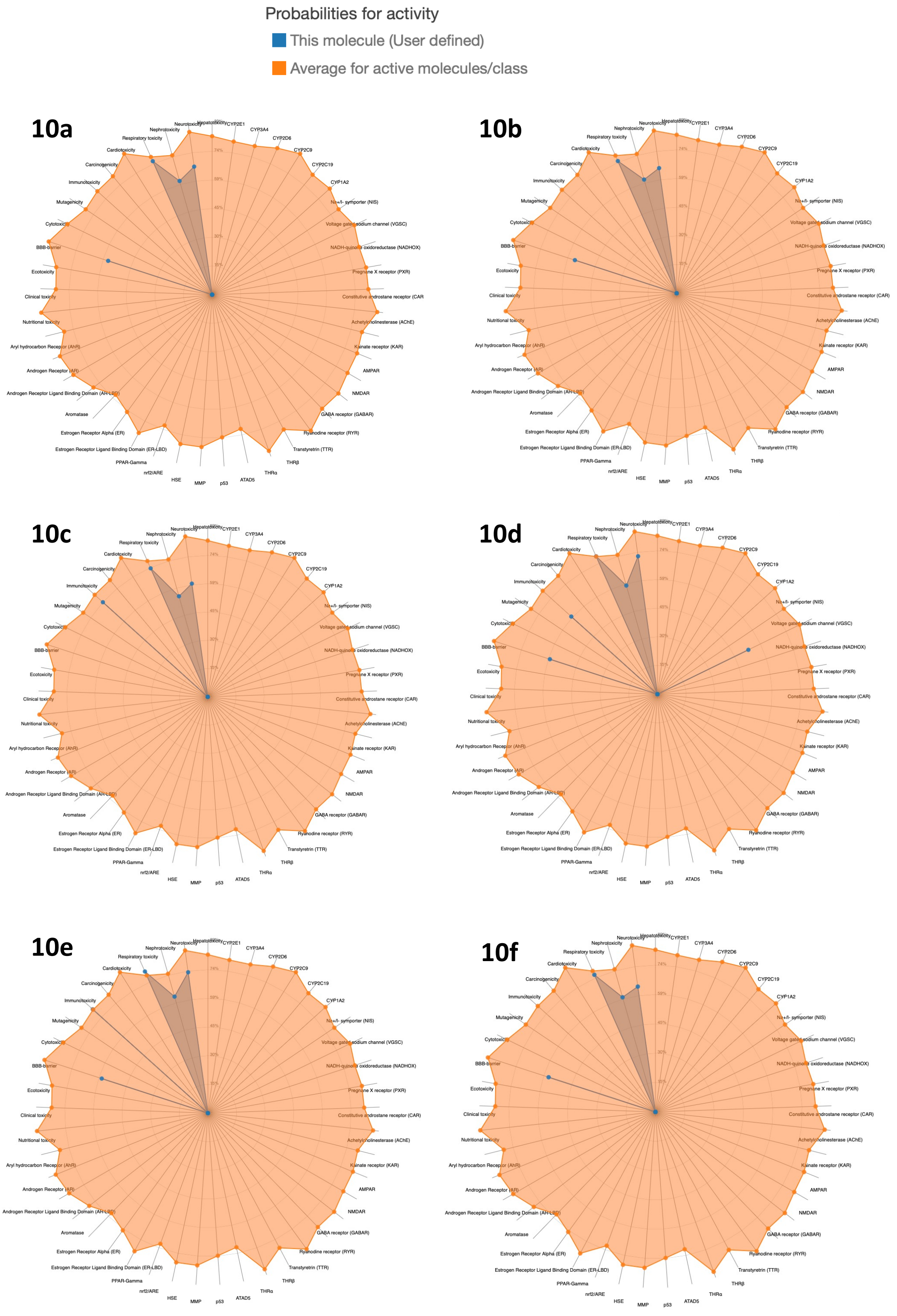

Continued on next page…

10g

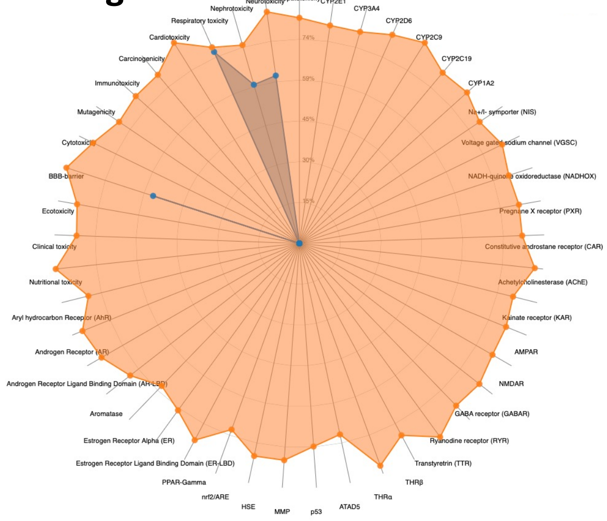

10h

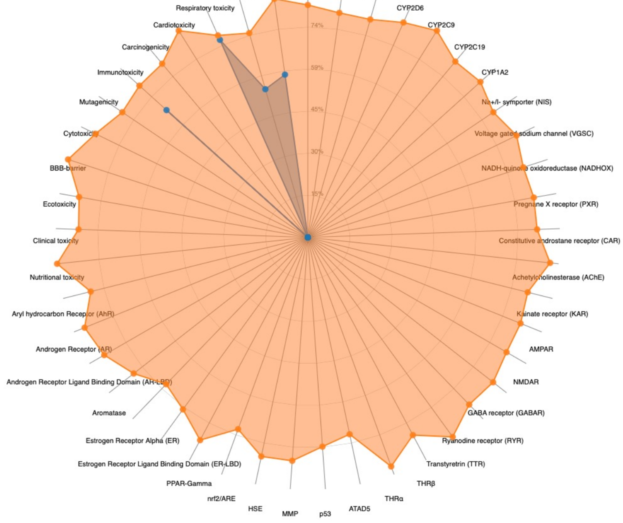

10i

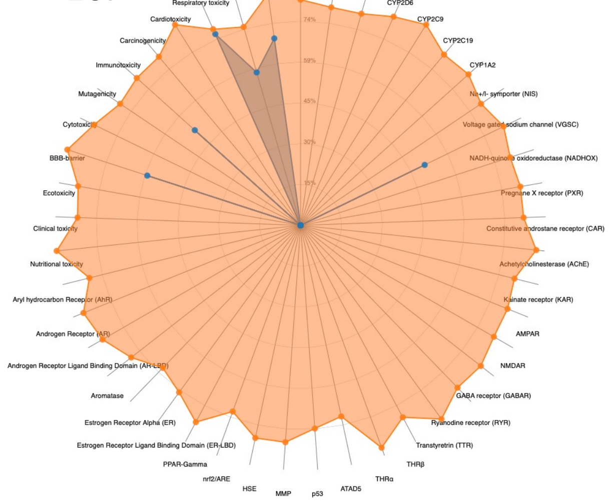

10j

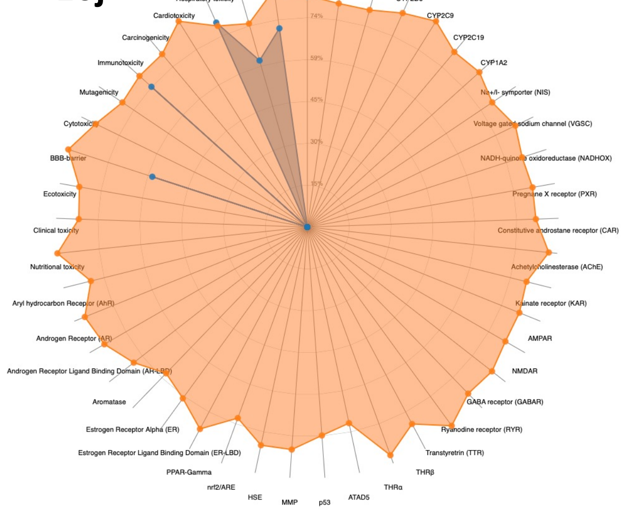

Figure S2: Predicted binding profiles of compounds **10a–j** toward a panel of key off-targets associated with adverse drug effects, as evaluated using the ProTox-3.0 web server.

The four-color heat map represents the probability of interaction, ranging from no binding (black) to probable binding (yellow–red scale). The selected off-target panel includes AA2AR (adenosine A2A receptor), ADRB2 (β2-adrenergic receptor), ANDR (androgen receptor), AOFA (amine oxidase A), CRFR1 (corticotropin-releasing factor receptor 1), DRD3 (dopamine D3 receptor), ESR1 and ESR2 (estrogen receptors α and β), GCR (glucocorticoid receptor), HRH1 (histamine H1 receptor), NR1I2 (pregnane X receptor), OPRK and OPRM (κ- and μ-opioid receptors), PDE4D (phosphodiesterase 4D), PGH1 (prostaglandin G/H synthase 1), and PRGR (progesterone receptor). Overall, no significant off-target binding liabilities were predicted for the investigated compounds.

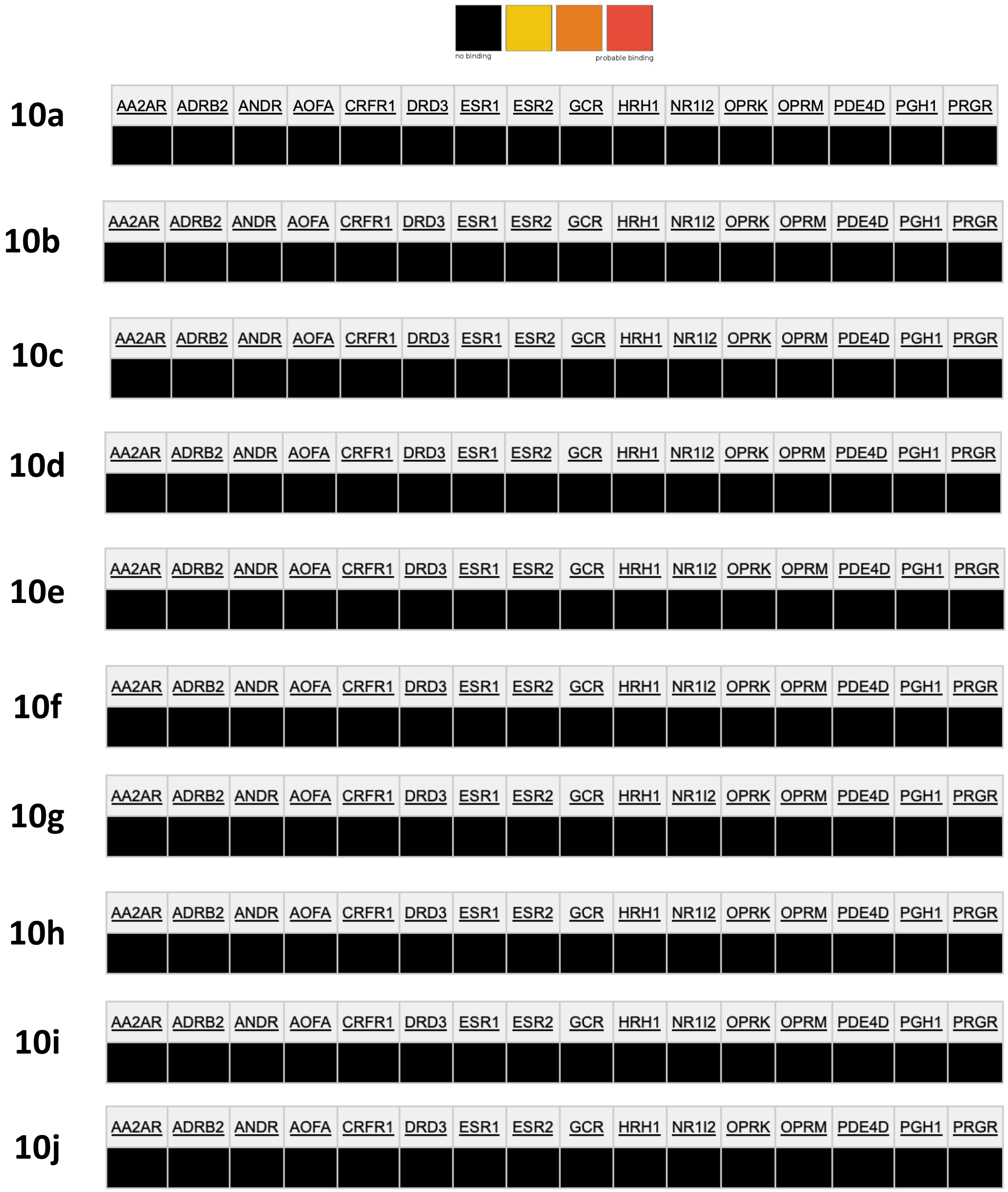

Figure S3: ^{1}HNMR spectrum of compound **10a** (400 MHz, d_6-DMSO)

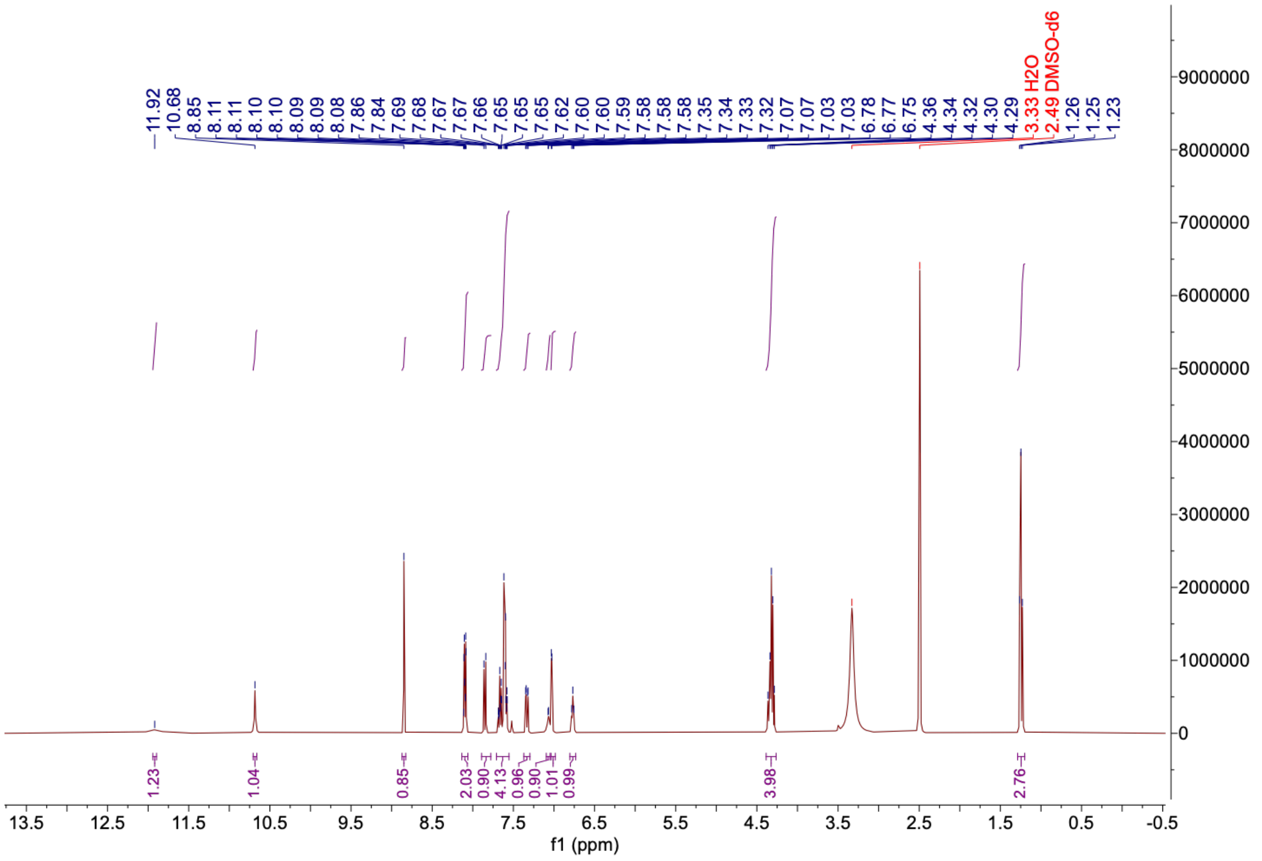

Figure S4: ^{13}CNMR spectrum of compound **10a** (100 MHz, d_6-DMSO)

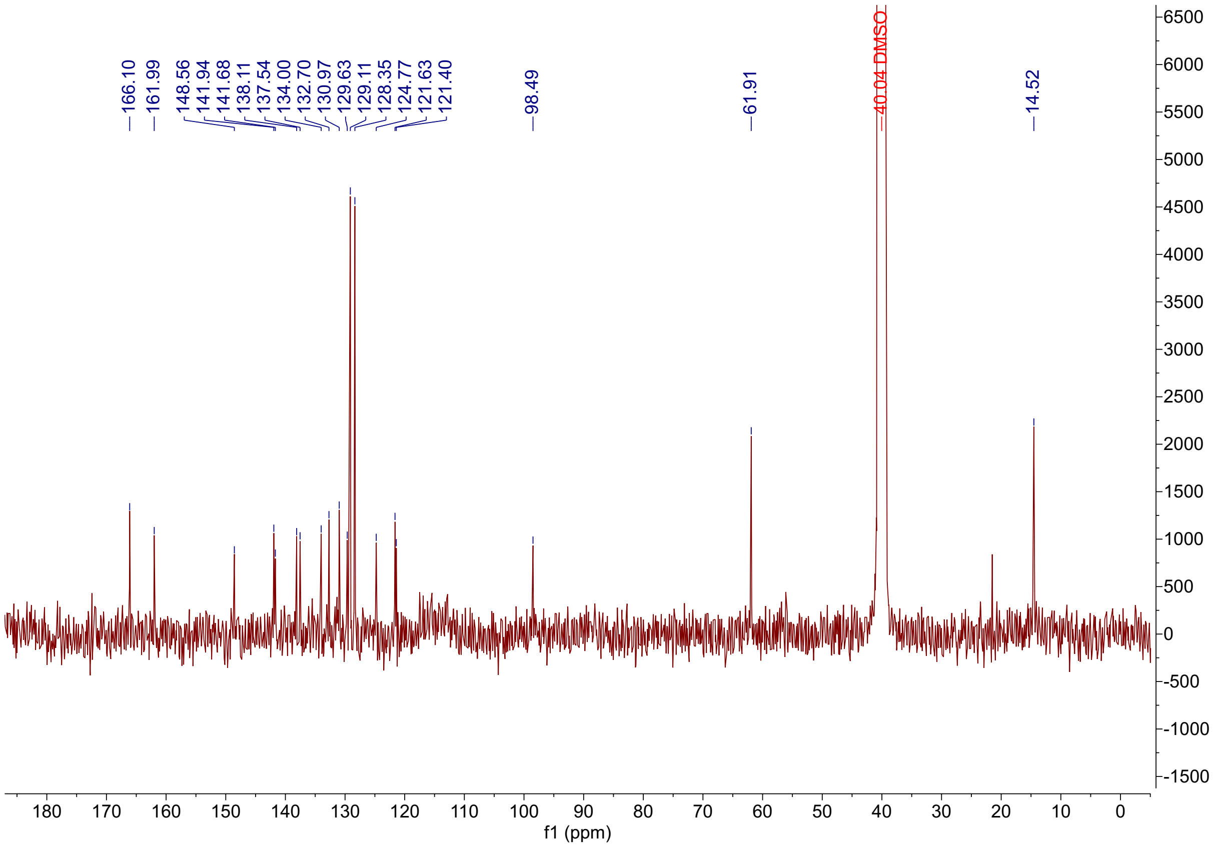

Figure S5: ^{1}HNMR spectrum of compound **10b** (400 MHz, d_6-DMSO)

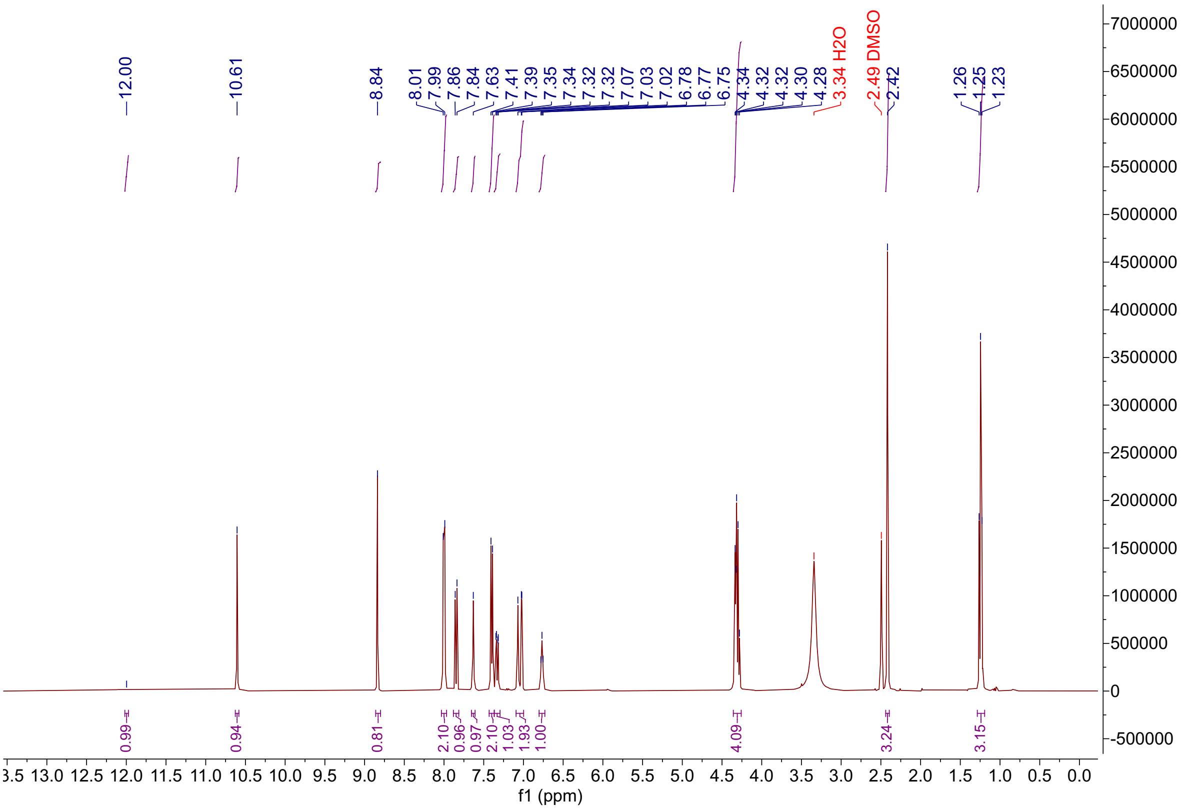

Figure S6: ^{13}CNMR spectrum of compound **10b** (100 MHz, d_6-DMSO)

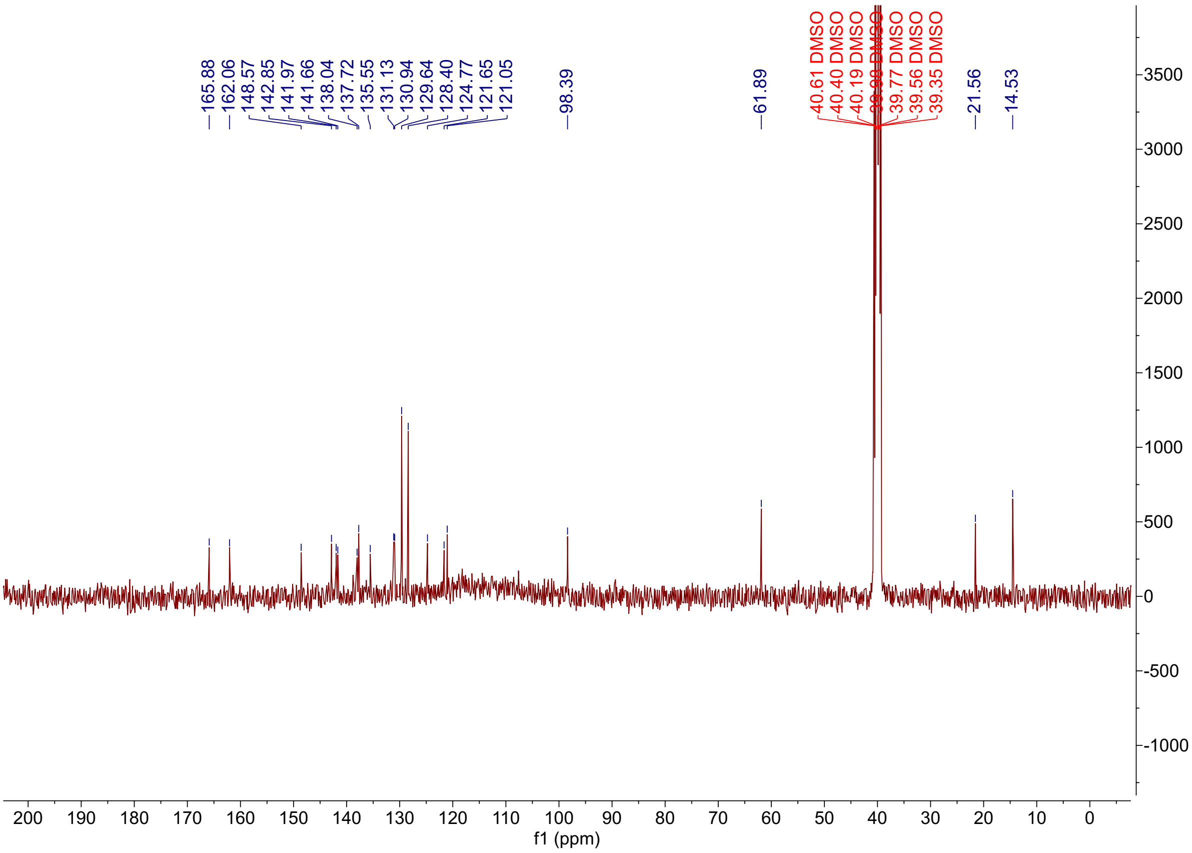

Figure S7: ^{1}HNMR spectrum of compound **10c** (400 MHz, d_6-DMSO)

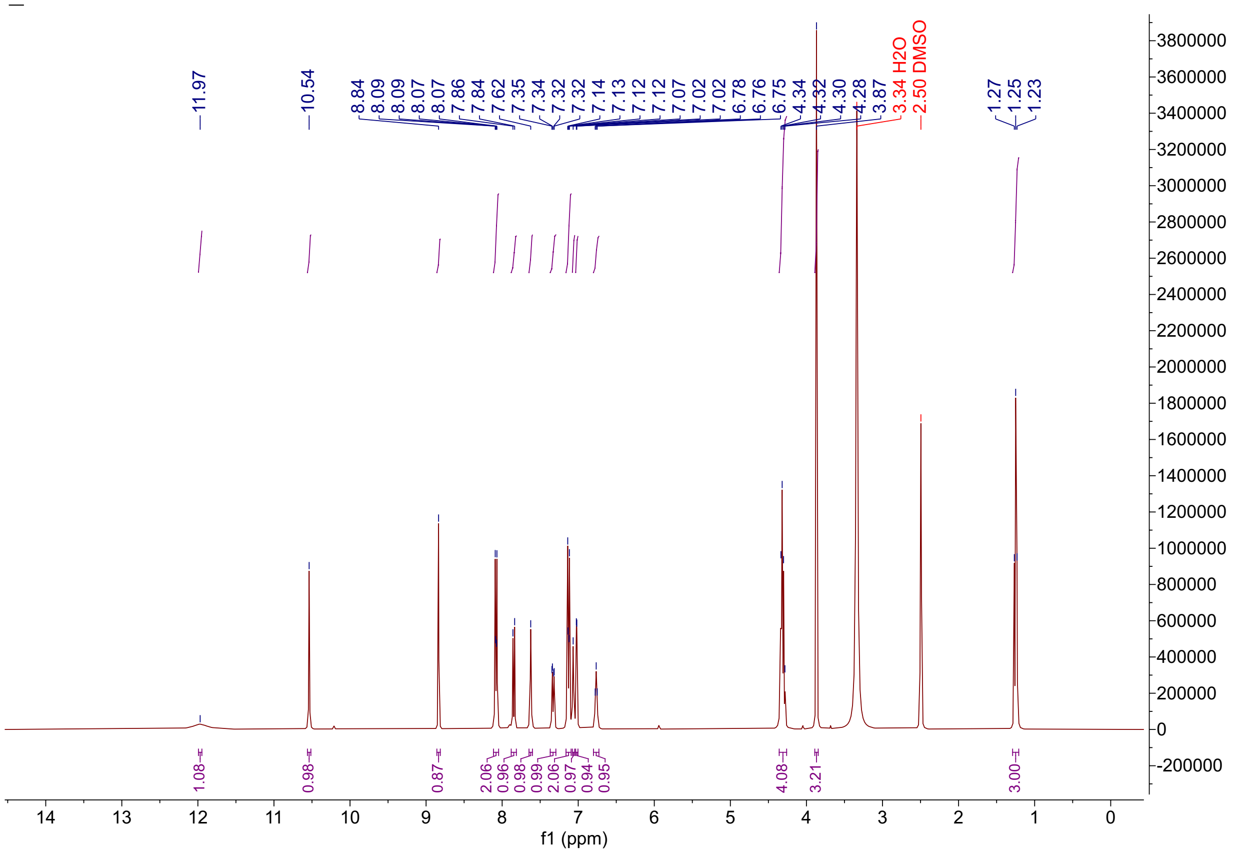

Figure S8: ^{13}CNMR spectrum of compound **10c** (100 MHz, d_6-DMSO)

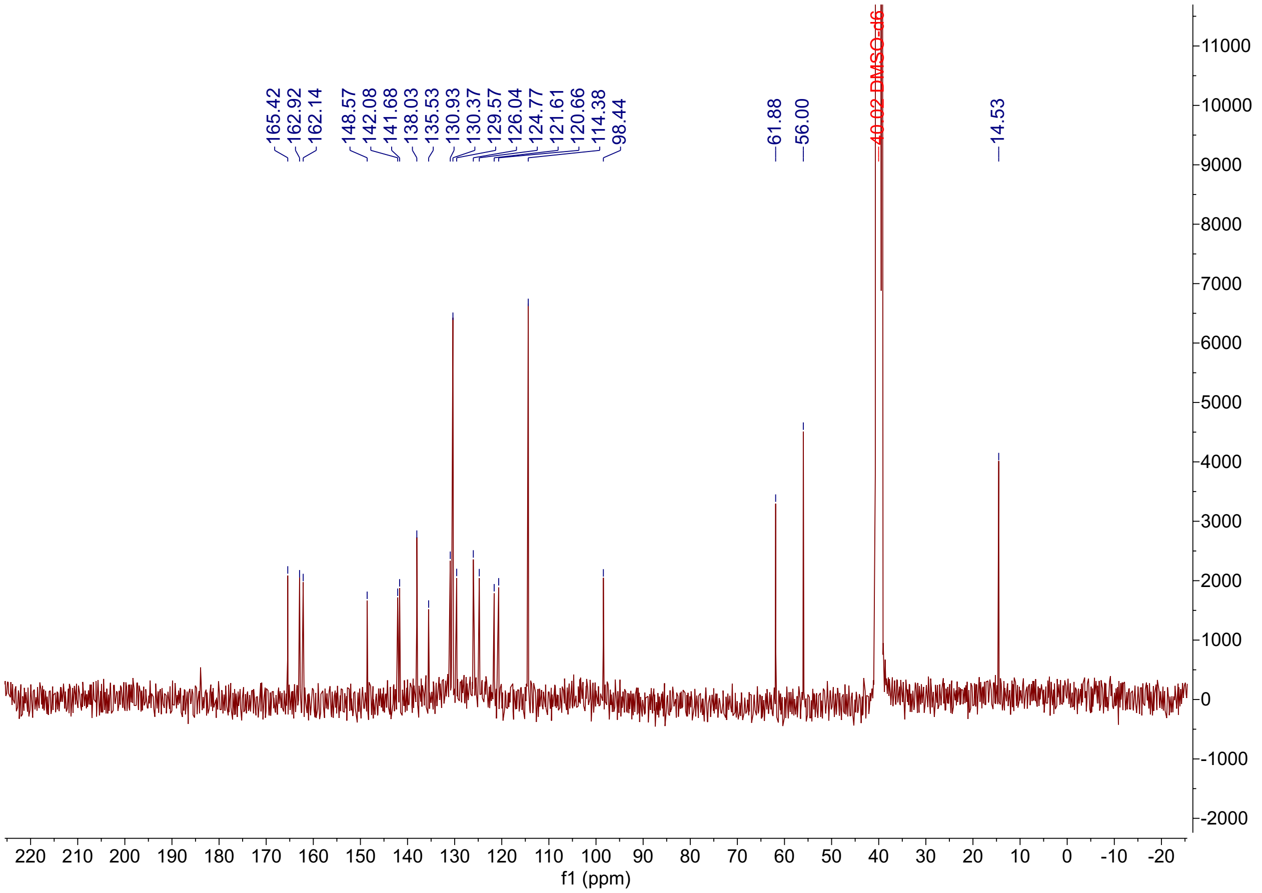

Figure S9: ^{1}HNMR spectrum of compound **10d** (400 MHz, d_6-DMSO)

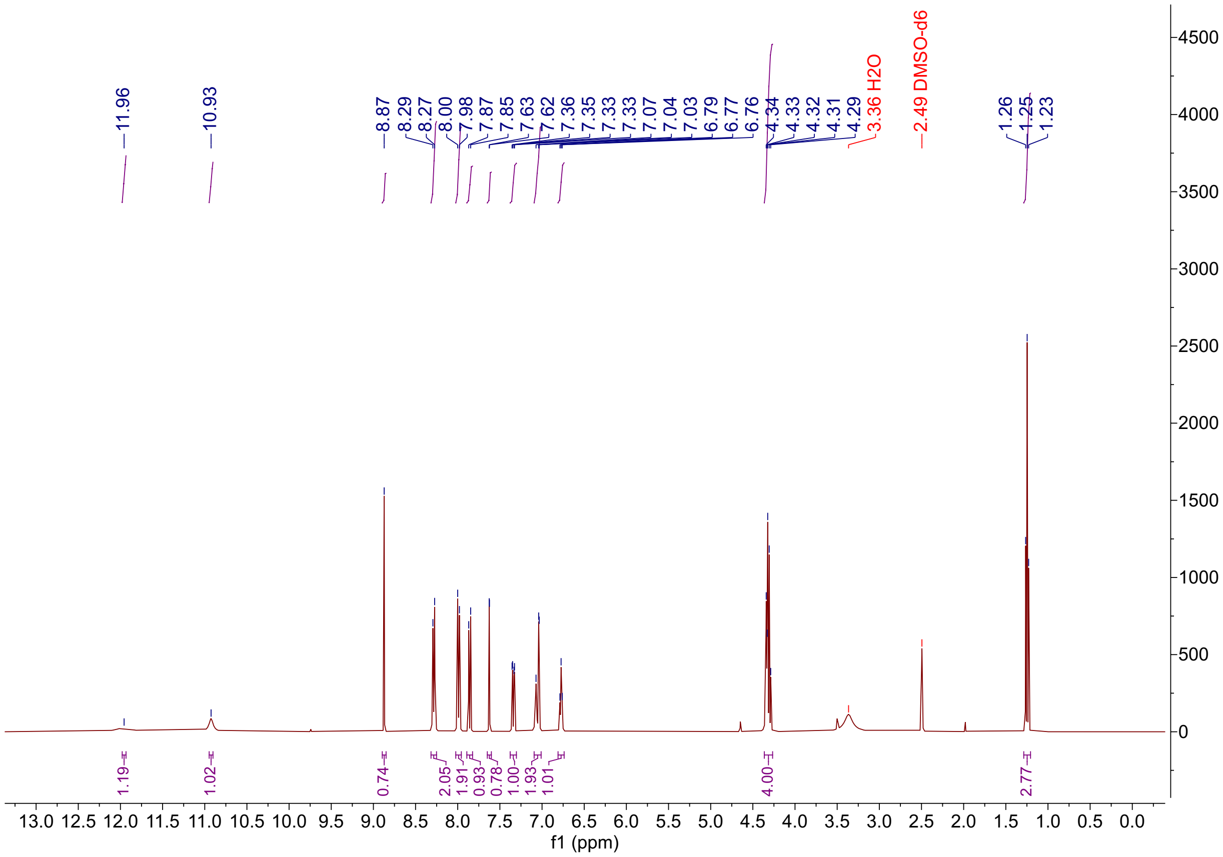

Figure S10: ^{13}CNMR spectrum of compound **10d** (100 MHz, d_6-DMSO)

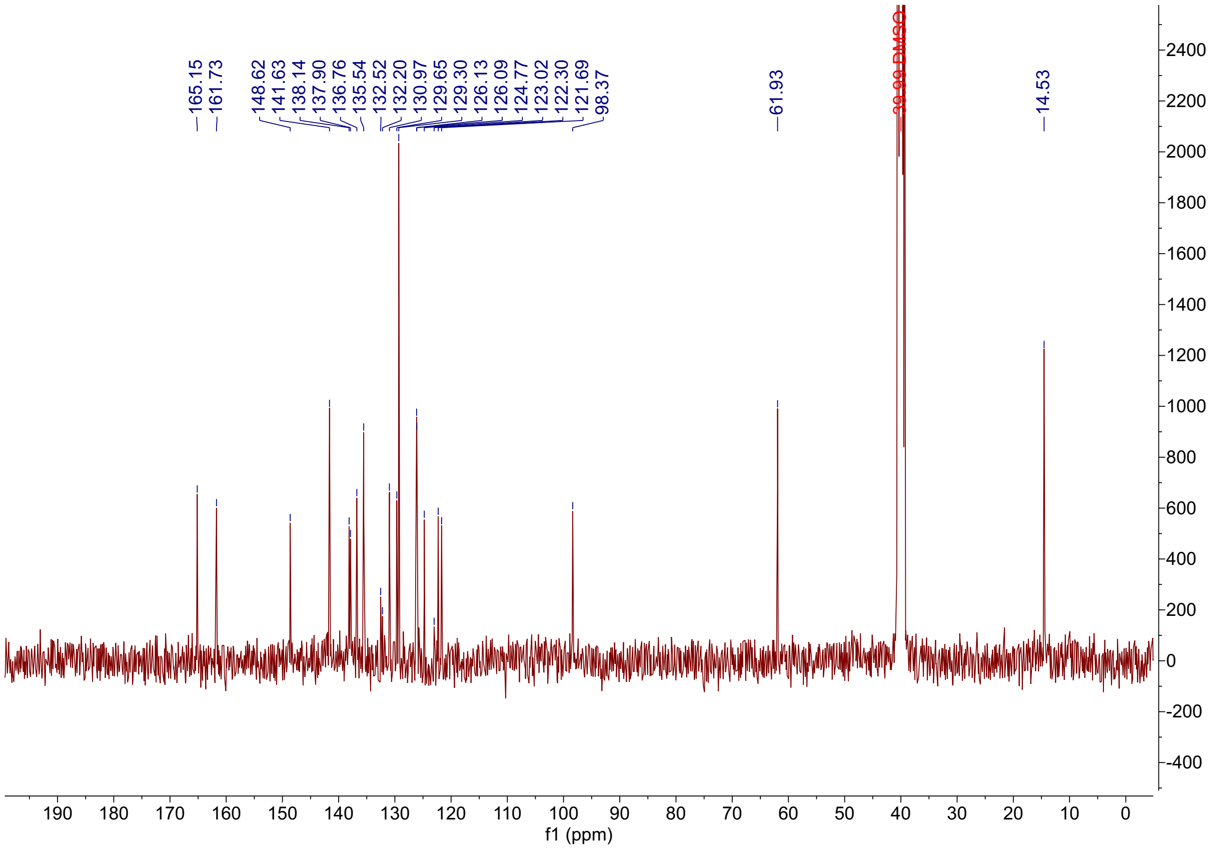

Figure S11: ^{1}HNMR spectrum of compound **10e** (400 MHz, d_6-DMSO)

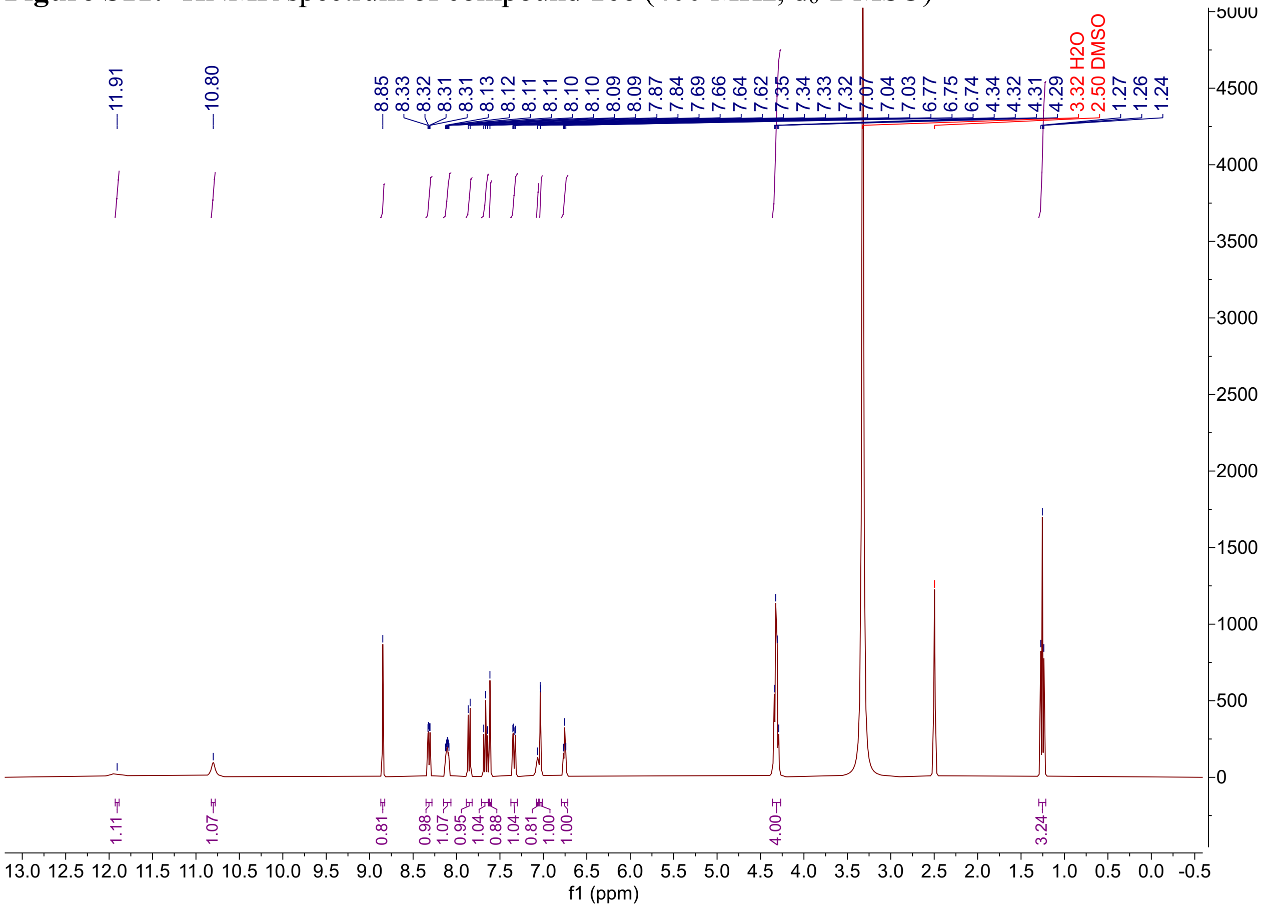

Figure S12: ^{13}CNMR spectrum of compound **10e** (100 MHz, d_6-DMSO)

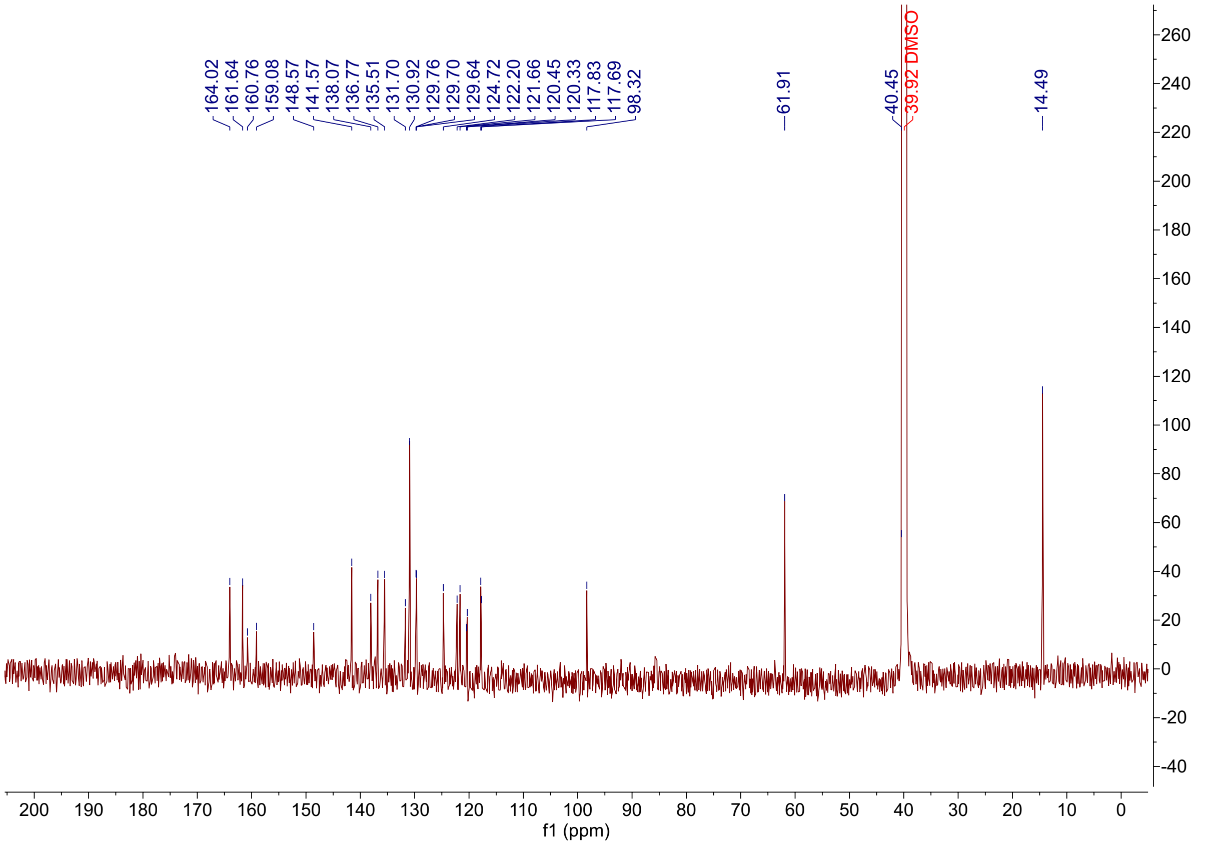

Figure S13: ^{1}HNMR spectrum of compound **10f** (400 MHz, d_6-DMSO)

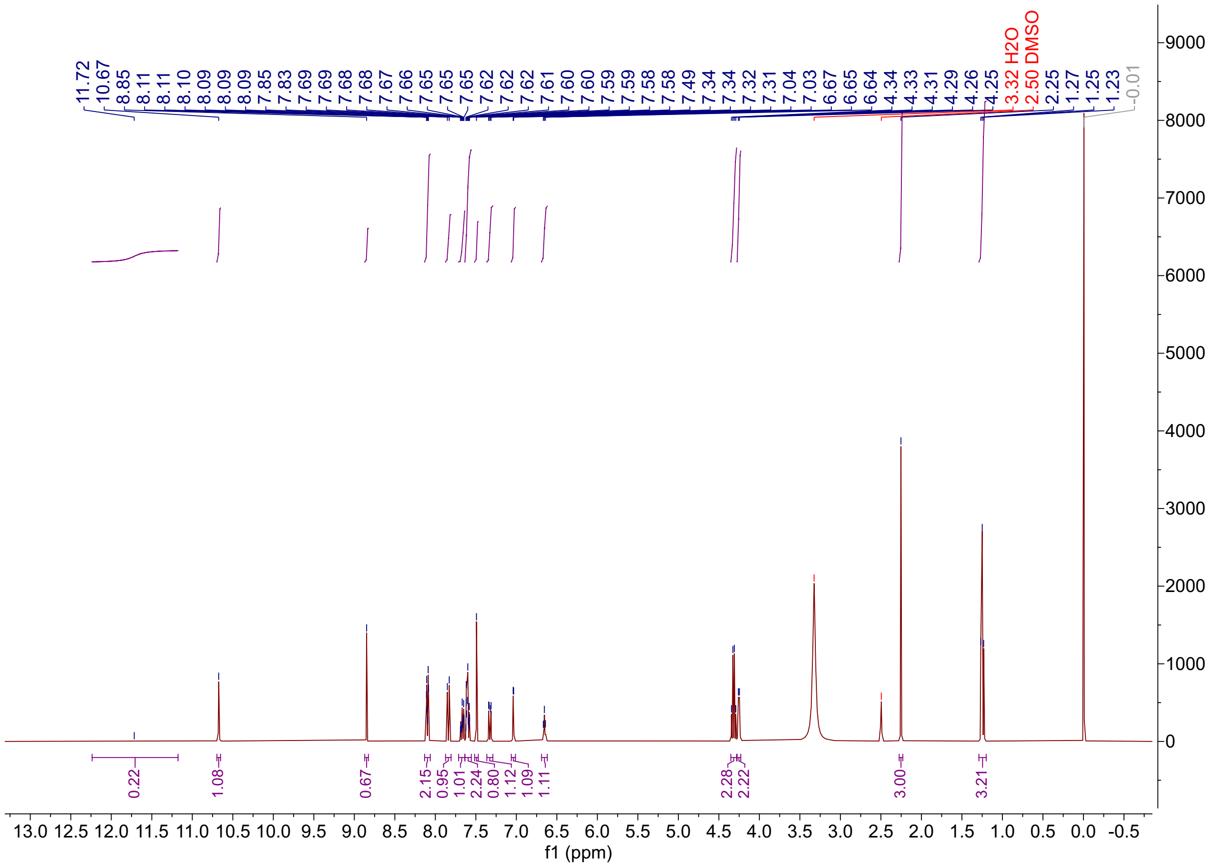

Figure S14: ^{13}CNMR spectrum of compound **10f** (100 MHz, d_6-DMSO)

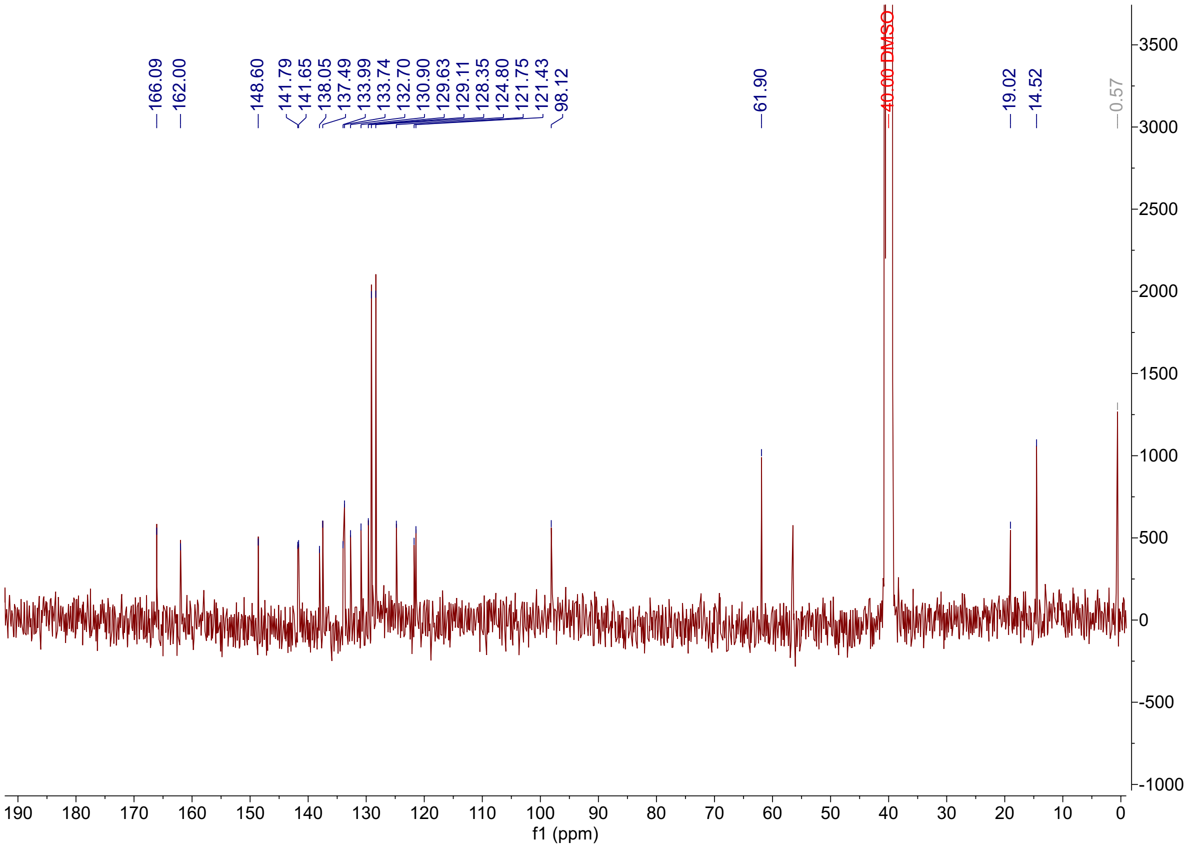

Figure S15: ^{1}HNMR spectrum of compound **10g** (400 MHz, d_6-DMSO)

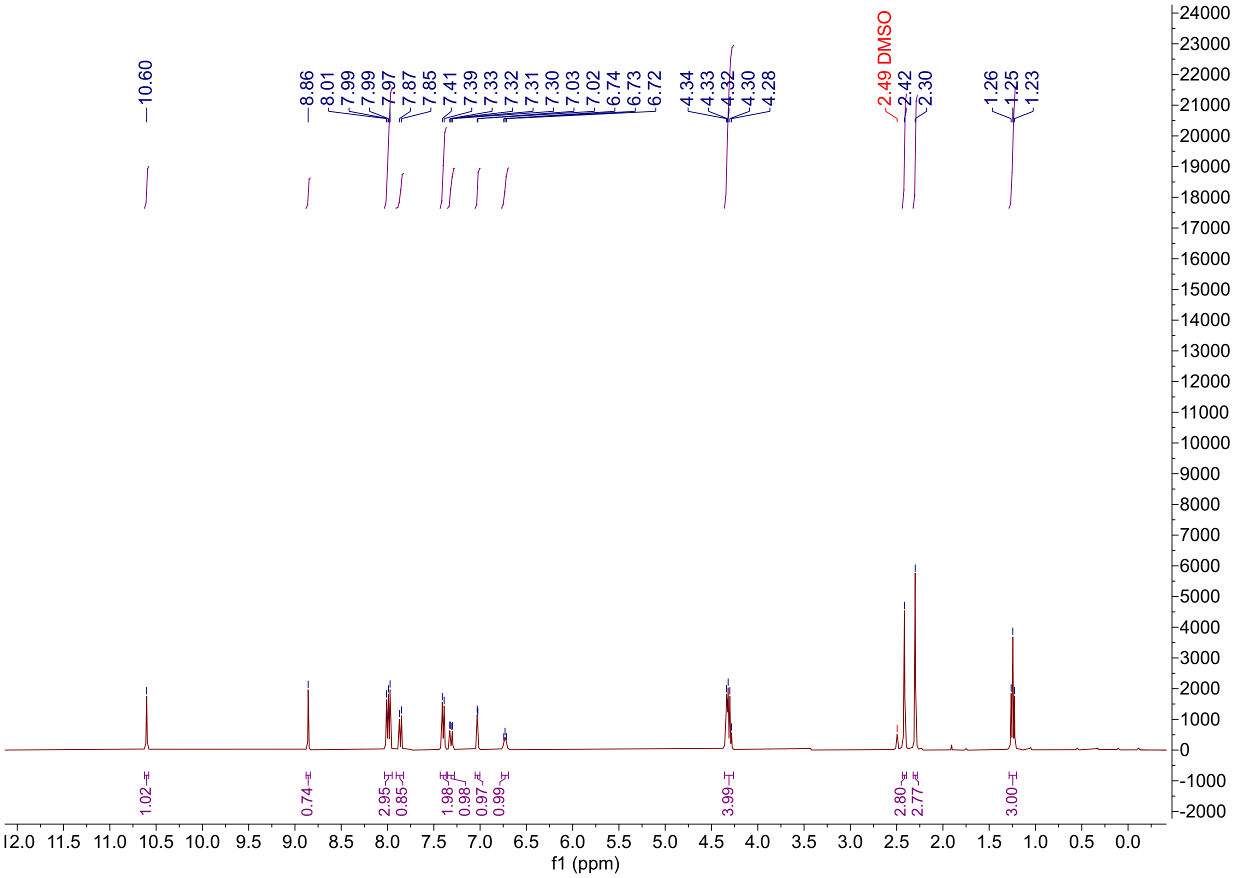

Figure S16: ^{13}CNMR spectrum of compound **10g** (100 MHz, d_6-DMSO)

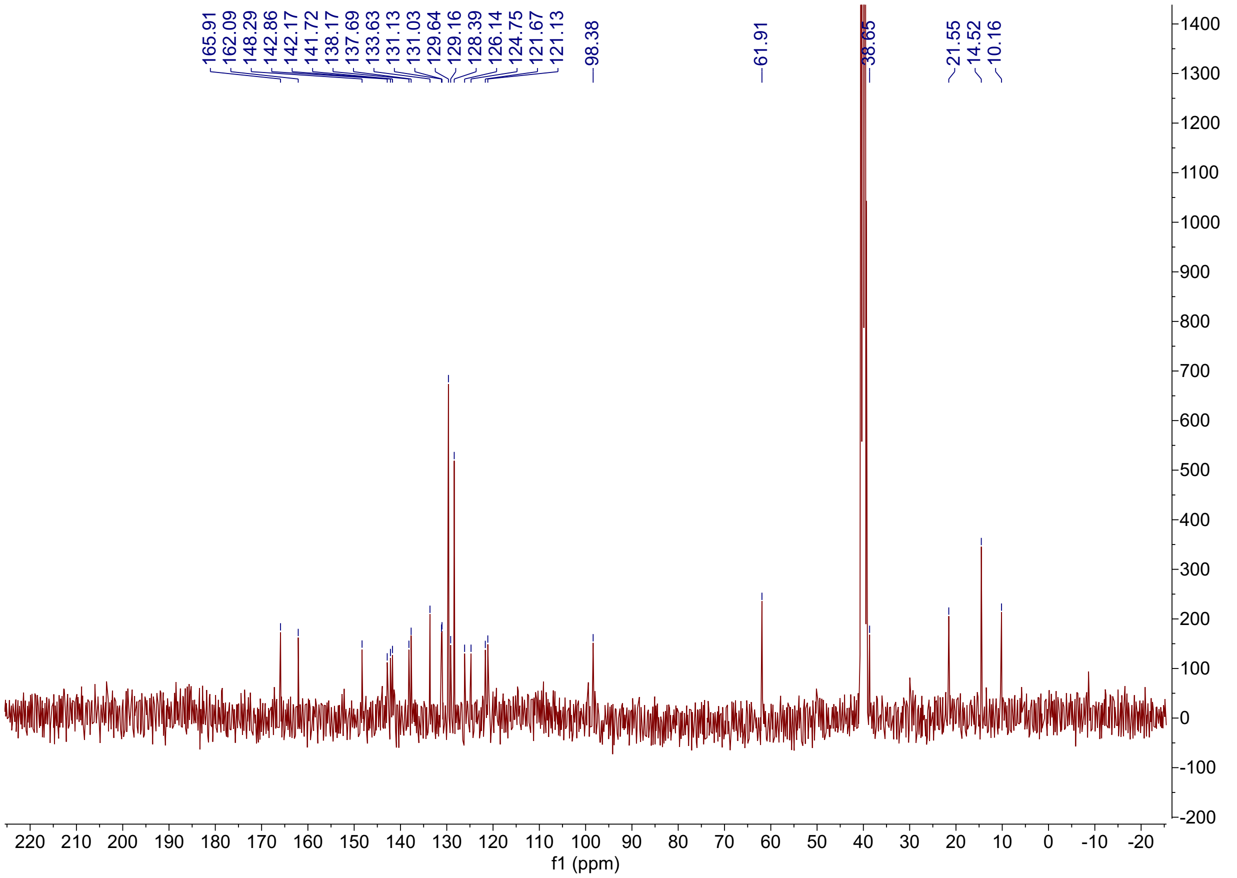

Figure S17: ^{1}HNMR spectrum of compound **10h** (400 MHz, d_6-DMSO)

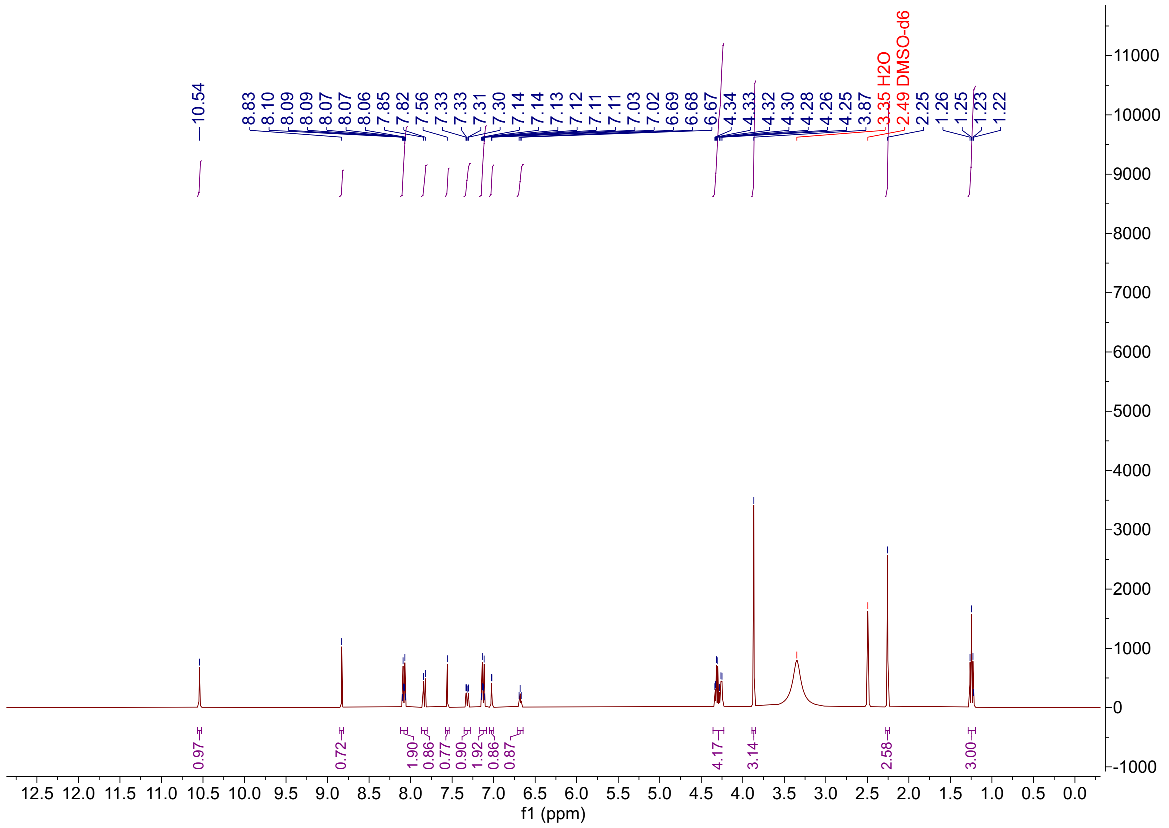

Figure S18: ^{13}CNMR spectrum of compound **10h** (100 MHz, d_6-DMSO)

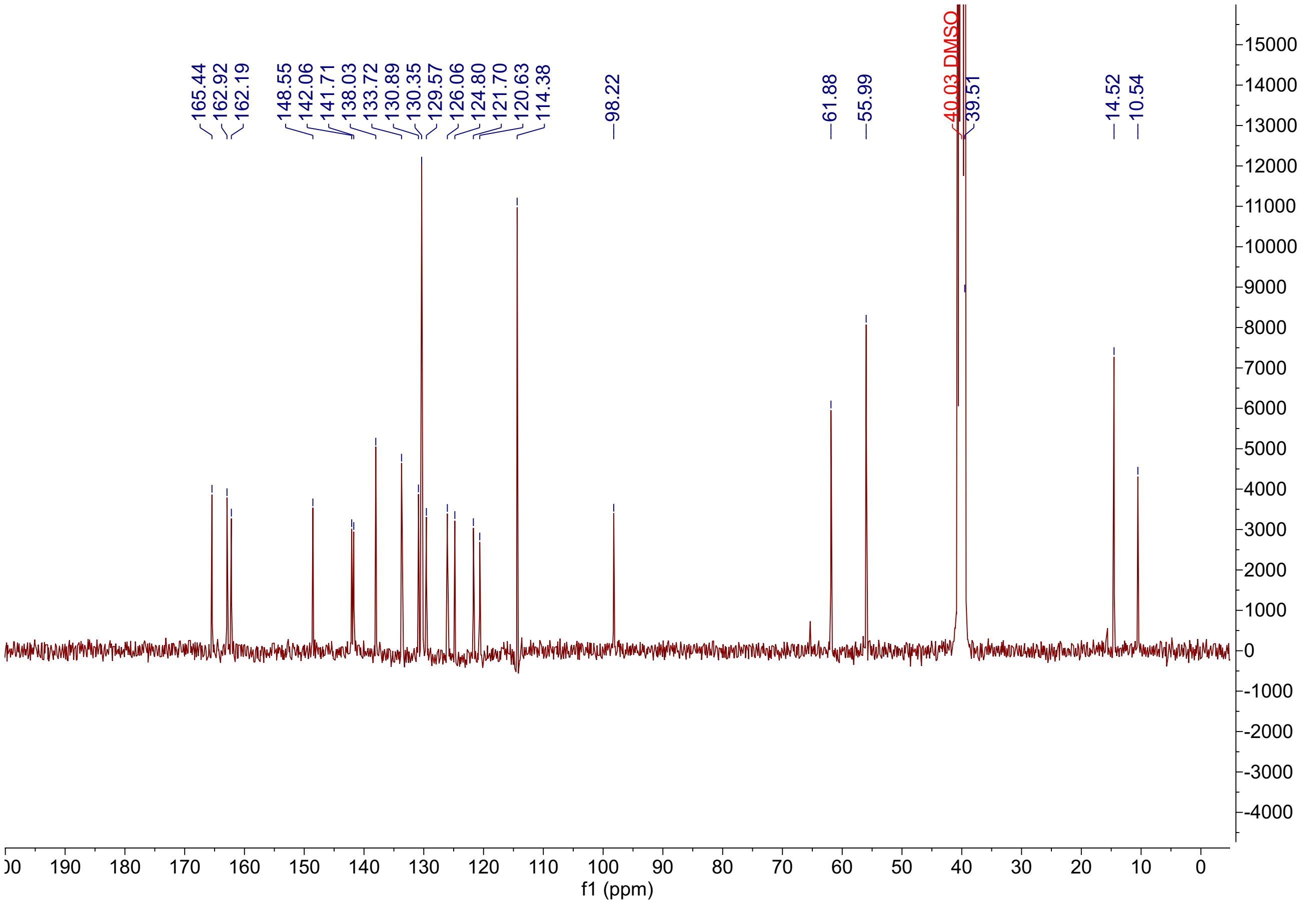

Figure S19: ^{1}HNMR spectrum of compound **10i** (400 MHz, d_6-DMSO)

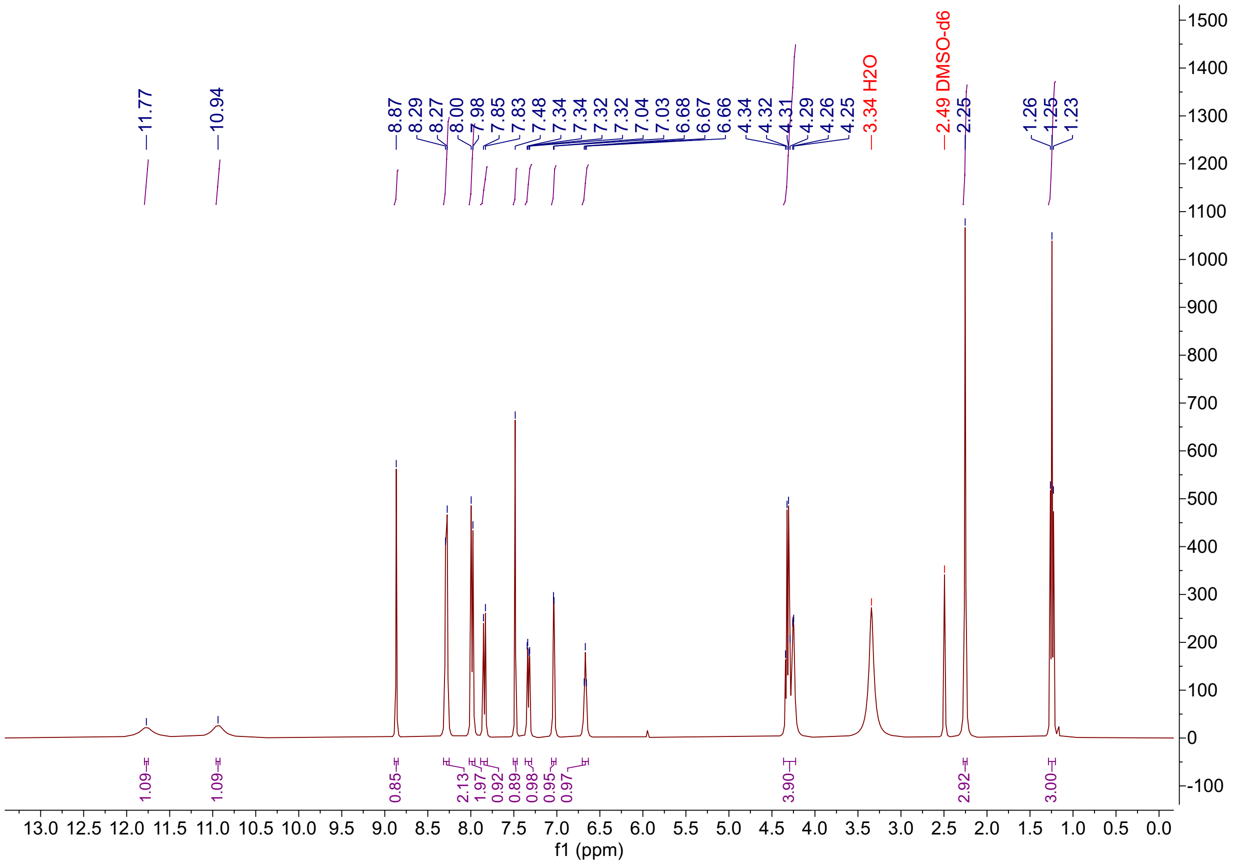

Figure S20: ^{13}CNMR spectrum of compound **10i** (100 MHz, d_6-DMSO)

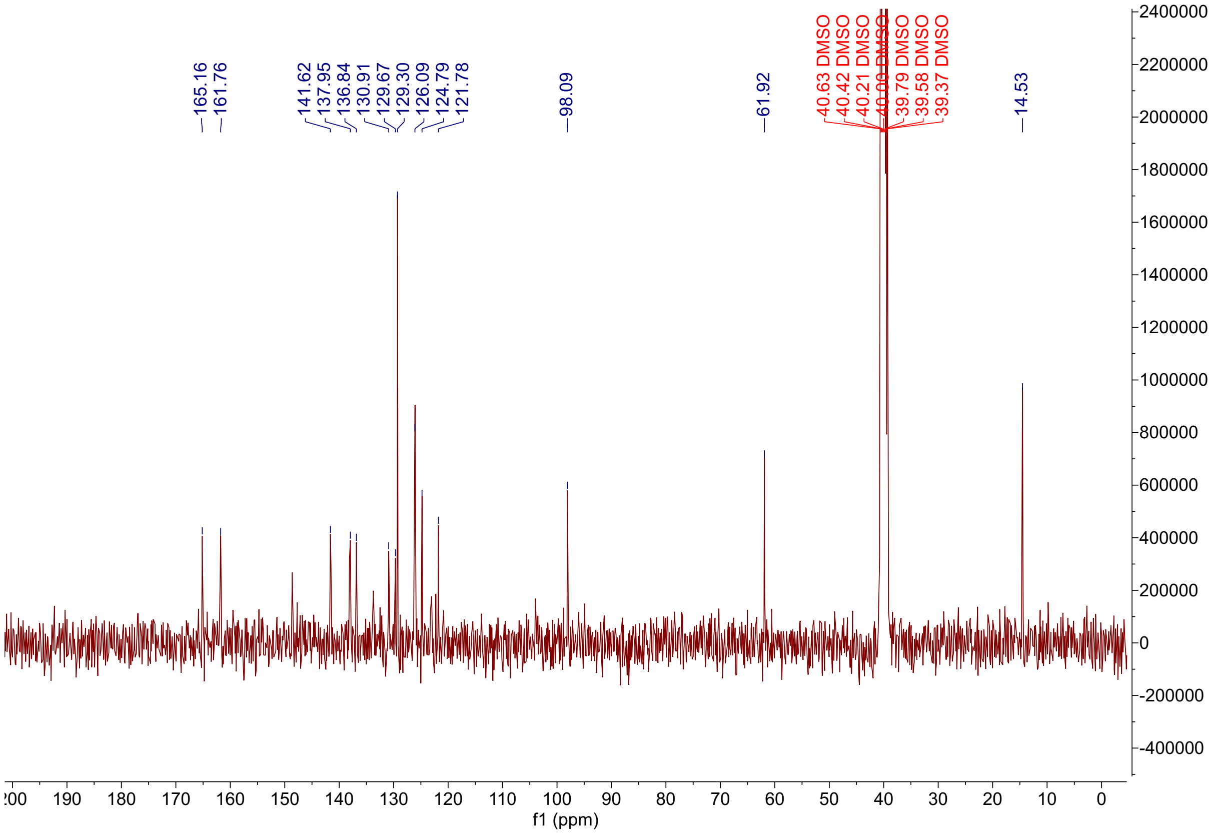

Figure S21: ^{1}HNMR spectrum of compound **10j** (400 MHz, d$_6$-DMSO)

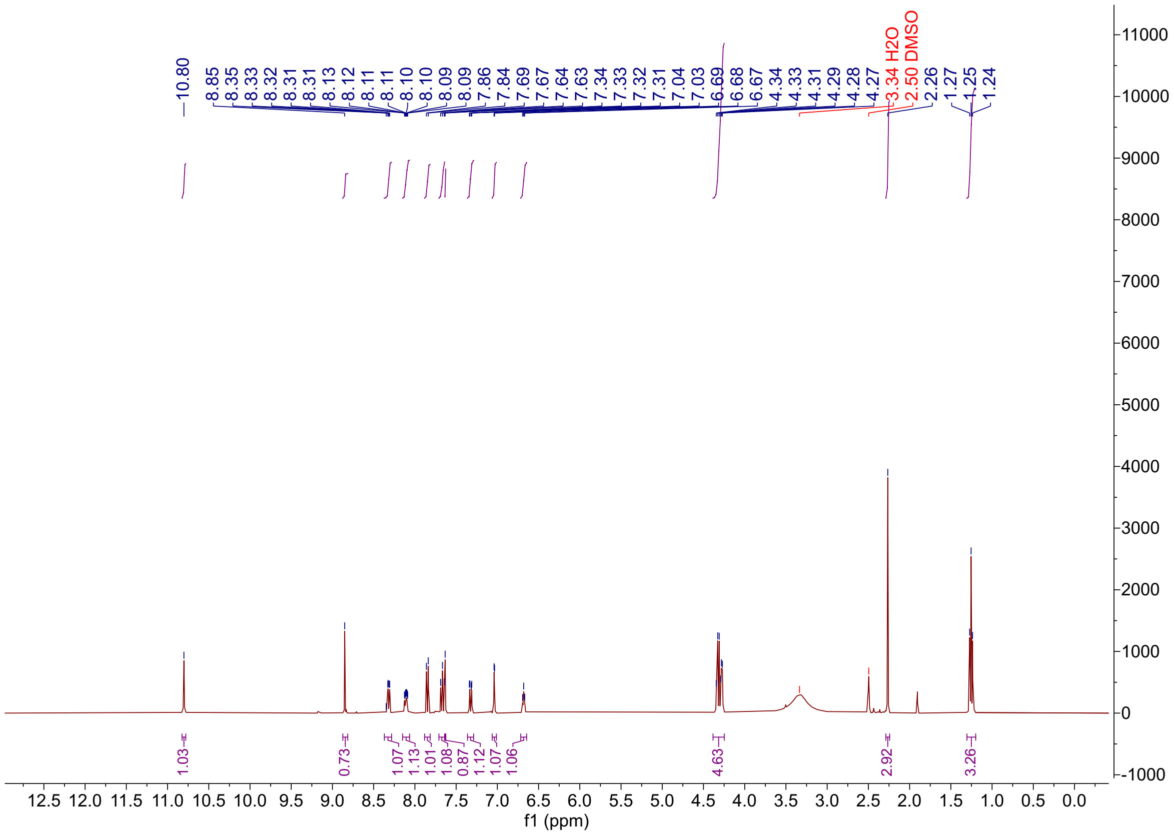

Figure S22: ^{13}CNMR spectrum of compound **10j** (100 MHz, d$_6$-DMSO)

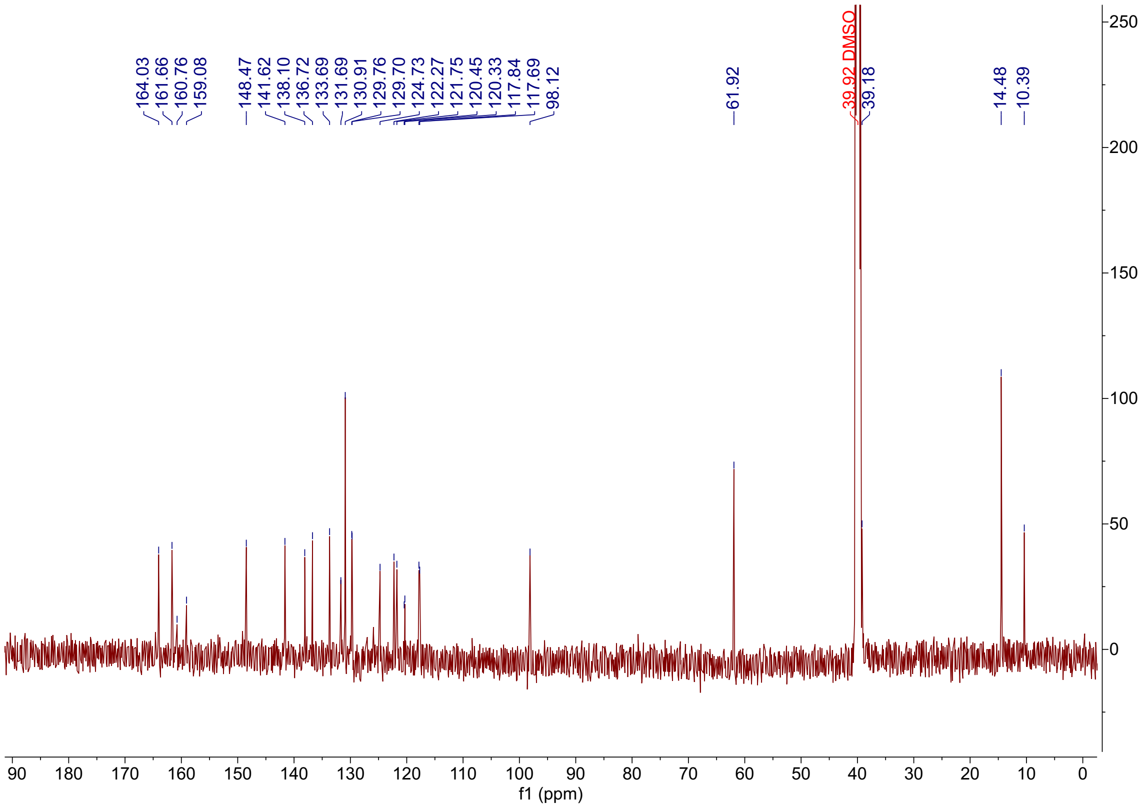

Figure S23: Stacked ^{1}HNMR spectra for compounds **10a–j** (400 MHz, d_6-DMSO)

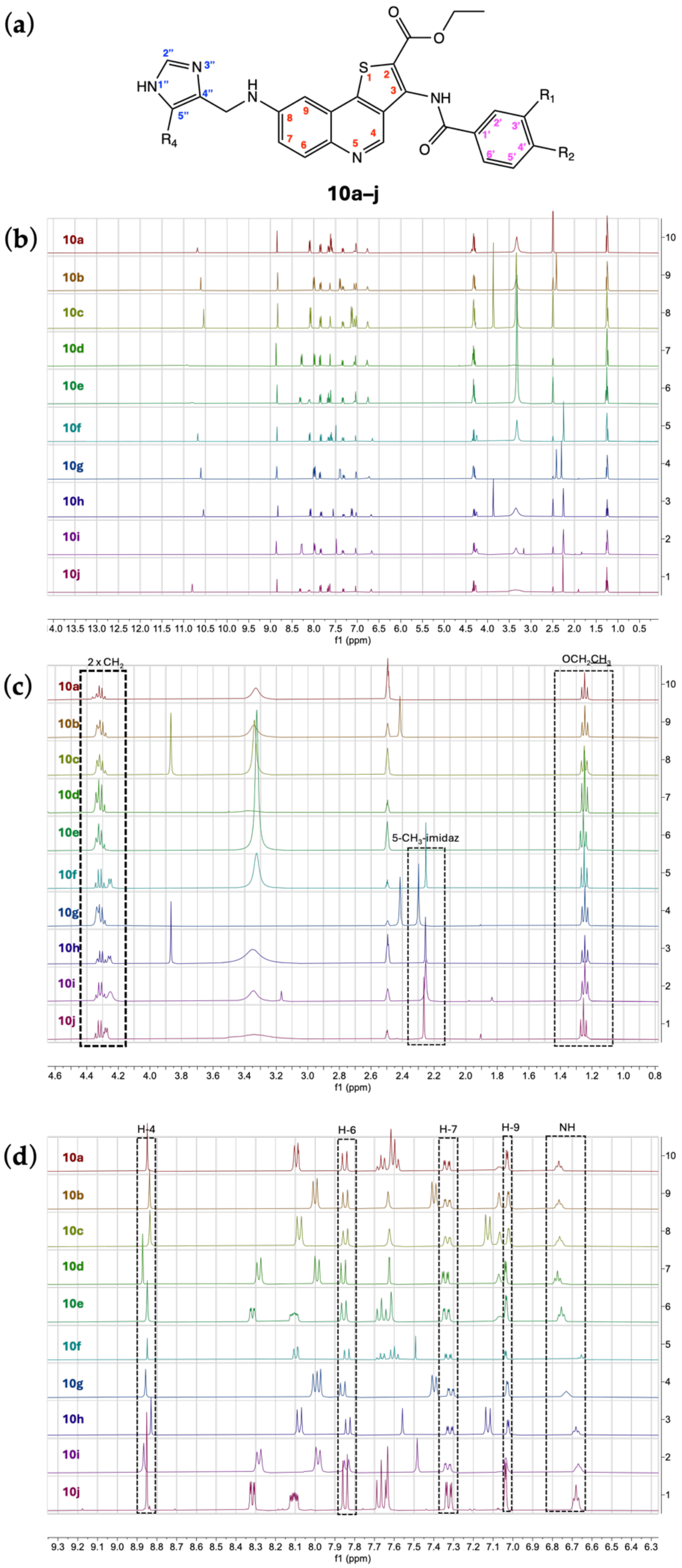

(**a**) General chemical structure of the imidazol-thienoquinoline derivatives **10a–j**, with appropriate numbering of the positions (X, X′, and X″ indicate the substitution sites on the thienoquinoline core, the benzoyl moiety, and the imidazole ring, respectively); (**b**) Stacked 1HNMR spectra of the final compounds **10a–j**; (**c**) Expanded view of the aliphatic region (0–5 ppm), highlighting the signals of the ethoxycarbonyl fragment, the methylene group adjacent to the imidazole ring, and the methyl group present in the methyl-imidazole derivatives **10f–j**; (**d**) Expanded view of the aromatic region (6–9.5 ppm), highlighting the characteristic signals of the NH, the thienoquinoline and imidazole moieties. All spectra were recorded in DMSO-d_6.

Figure S24: Detailed ^{13}C NMR assignment and 2D NMR characterization of representative compound **10c.**

(**a**) Chemical structure of compound **10c** with assigned ^{13}C NMR chemical shifts (δC, ppm). (**b**) DEPT-135 spectrum of compound **10c**, allowing differentiation of CH, CH_2, and CH_3 carbon signals. (**c**) ^1H–^{13}C HSQC spectrum of compound **10c**, showing direct one-bond correlations between proton (δH, ppm, horizontal axis) and carbon (δC, ppm, vertical axis) chemical shifts. (**d**) ^1H–^{13}C HMBC spectrum of compound **10c**, highlighting long-range heteronuclear correlations (^2J and ^3J) used to confirm the carbon framework and complete signal assignment.

(a)

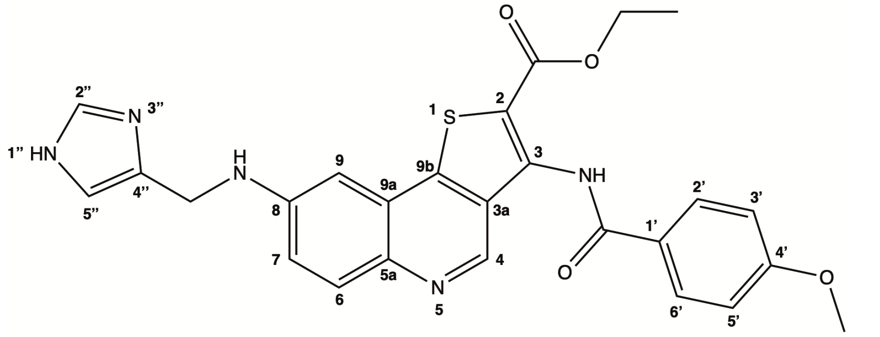

^{13}C NMR (DMSO-d_6) δ: 14.5 (q, $OCH_2\underline{CH_3}$), 39.5 (t, $NH\underline{CH_2}$), 56.0 (q, OCH_3), 61.9 (t, $O\underline{CH_2}CH_3$), 98.4 (d, CH, C9), 114.4 (dx2, CH, C3', C5', C5''), 120.7 (s, C2), 121.6 (s, CH, C7), 124.8 (s, C3a), 126.0 (s, C9a), 129.6 (s, C1'), 130.4 (s+d, C+CH, C2', C6', C4''), 130.9 (d, CH, C6), 135.5 (d, CH, C2''), 138.0 (s, C5a), 141.7 (s, C8), 142.1 (s+d, C+CH, C4), 148.6 (s, C9b), 162.1 (s, C4'), 162.9 (s, $NH\underline{CO}$), 165.4 (s, $\underline{CO}OCH_2CH_3$).

(b)

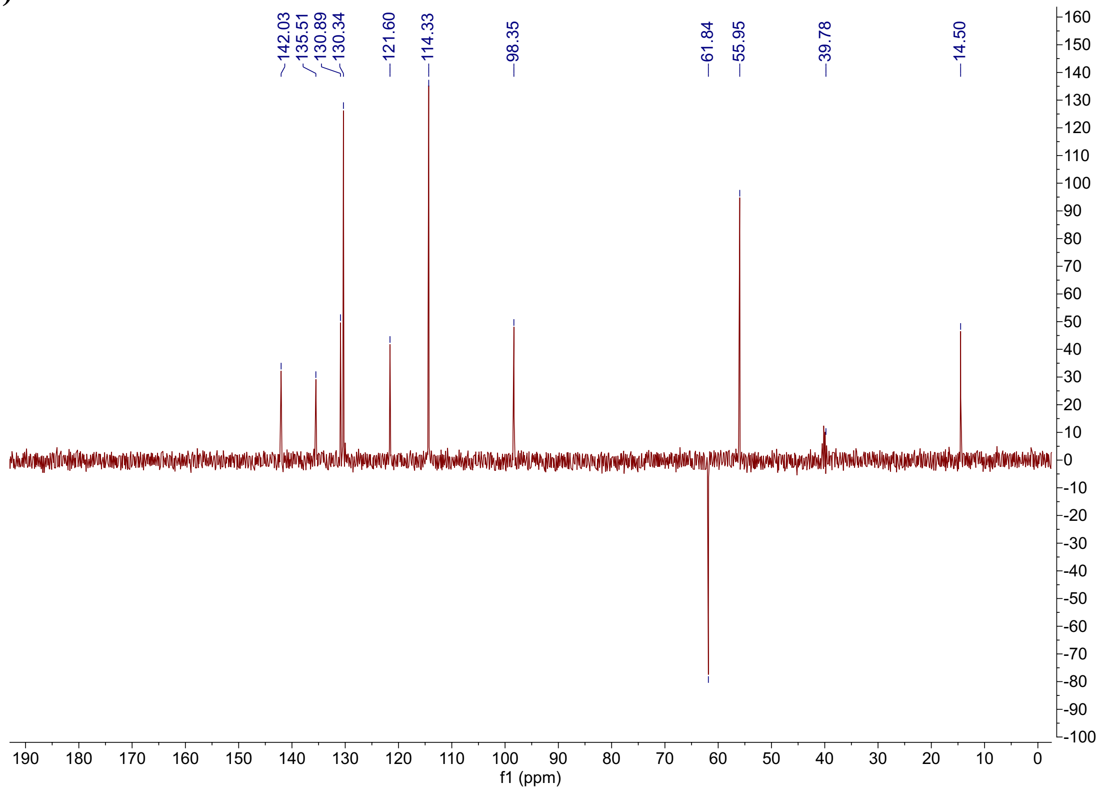

(c)

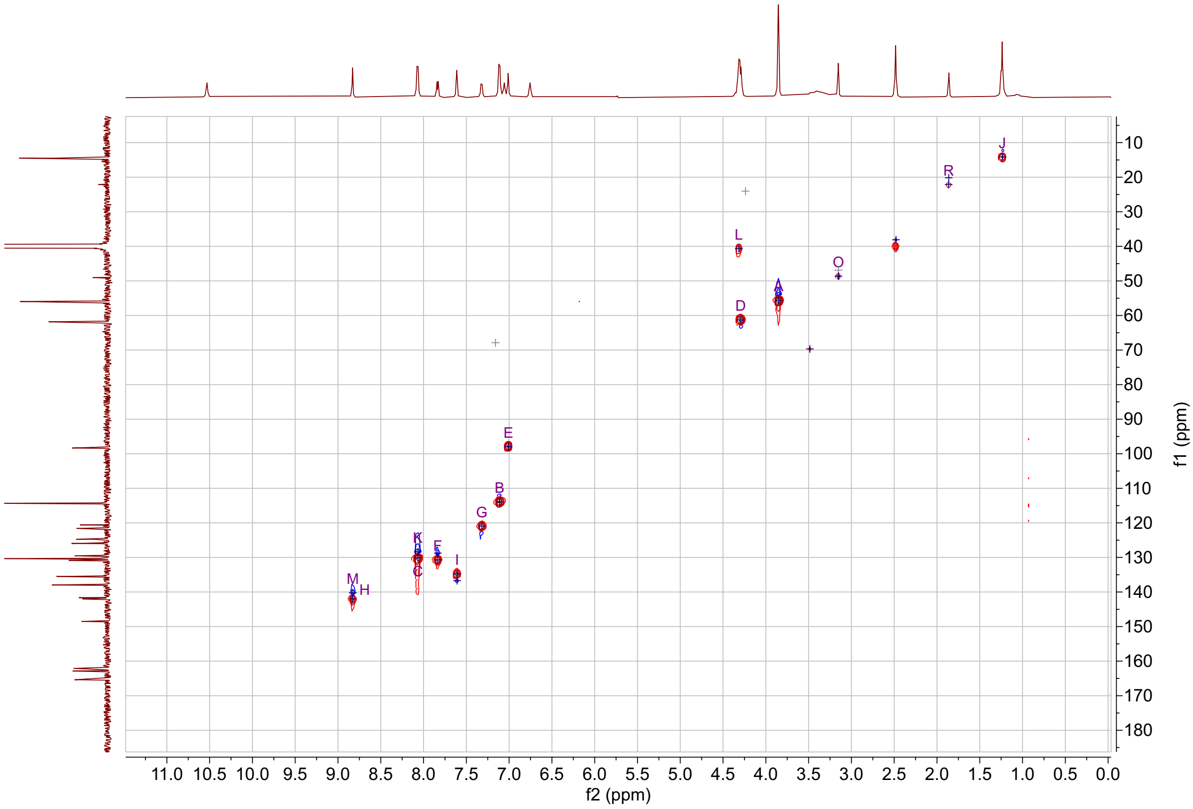

(d)

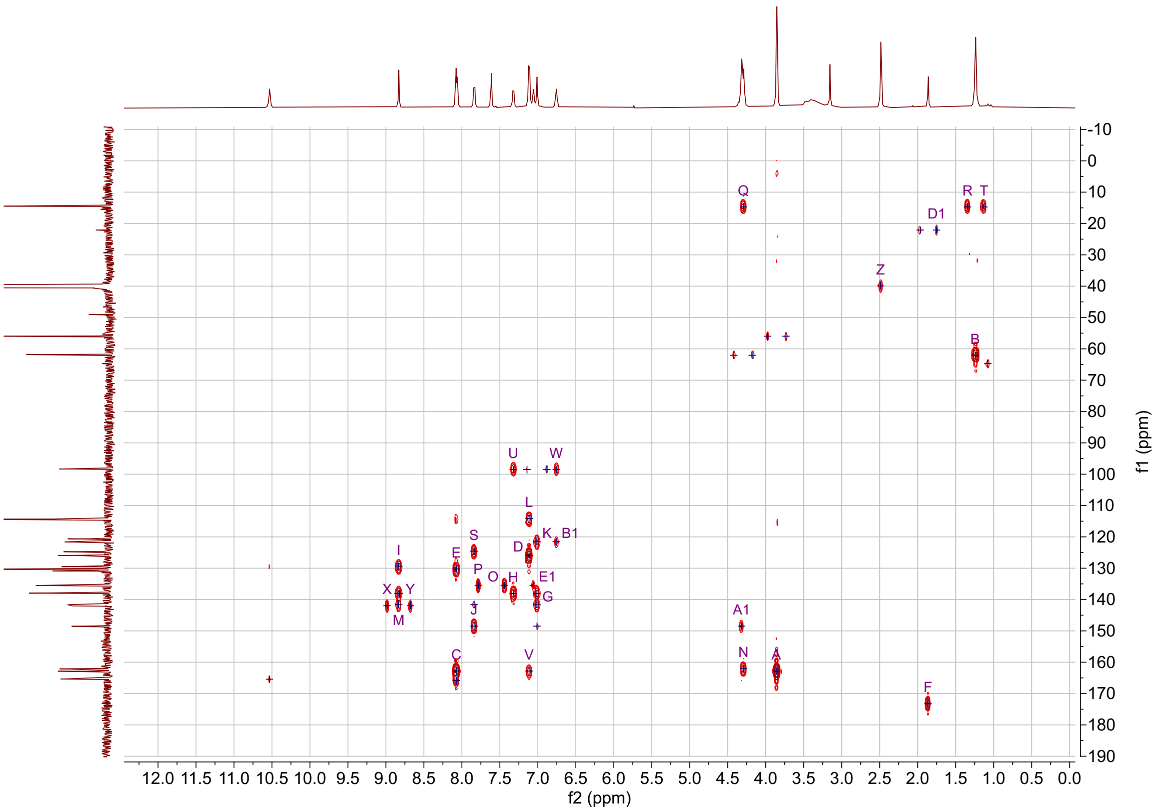

Figure S25: RMSD and 2D lig-interaction map analysis from MD simulations of co-crystallized reference ligands

Molecular dynamics (100 ns) simulations of the co-crystallized reference inhibitors in complex with PI3Kα (PDB ID: 8EXL), CK2 (PDB ID: 3NGA), PIM1 (PDB ID: 5O11), PARP1 (PDB ID: 7KK4), respectively. Protein backbone RMSD and ligand RMSD profiles are reported to enable direct comparison with the corresponding type **10** derivatives (panels **a–d**). Representative protein–ligand interaction diagrams showing persistent hydrogen bonds, electrostatic, and hydrophobic contacts observed during the trajectories (panels **e–h**).

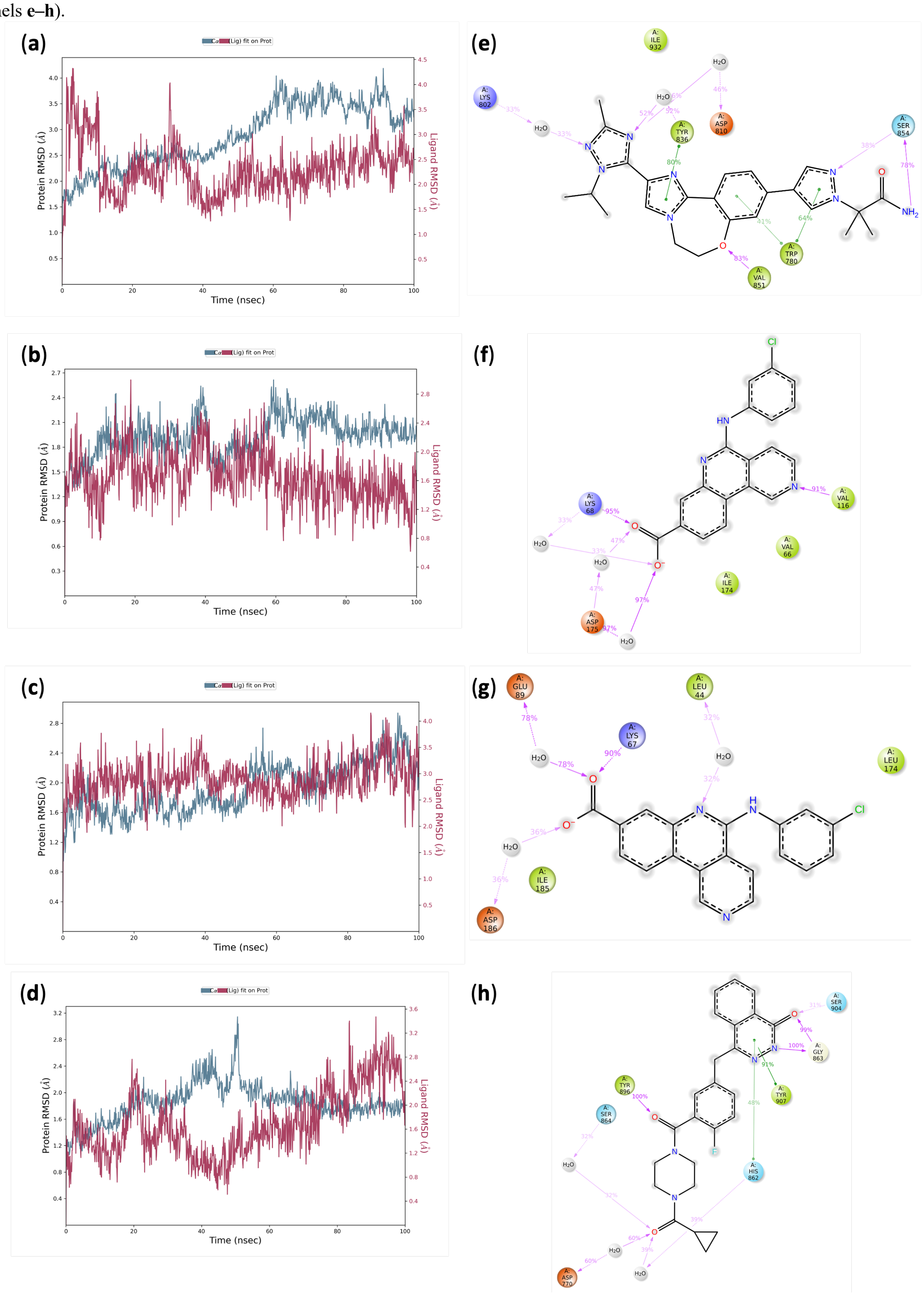

Bibliography

1. La Monica, G.; Pizzolanti, G.; Baiamonte, C.; Bono, A.; Alamia, F.; Mingoia, F.; Lauria, A.; Martorana, A. Design and Synthesis of Novel Thieno[3,2-c]Quinoline Compounds with Antiproliferative Activity on RET-Dependent Medullary Thyroid Cancer Cells. *ACS Omega* **2023**, *8*, 34640–34649, doi:10.1021/acsomega.3c03578.
2. Harwood, S.; Ketcham, J.M.; Ivetac, A.; Smith, C.R.; Marx, M.A.; Pearson, K.E.; Lawson, J.D. Substituted Tricyclic Compounds 2024.
3. Reddy, E.P.; Reddy, M.V.R. 3-aminothieno[3,2-c]Quinoline Derivatives, Methods of Preparation and Uses 2015.
4. Pierre, F.; Regan, C.F.; Chevrel, M.-C.; Siddiqui-Jain, A.; Macalino, D.; Streiner, N.; Drygin, D.; Haddach, M.; O'Brien, S.E.; Rice, W.G.; et al. Novel Potent Dual Inhibitors of CK2 and Pim Kinases with Antiproliferative Activity against Cancer Cells. *Bioorg. Med. Chem. Lett.* **2012**, *22*, 3327–3331, doi:10.1016/j.bmcl.2012.02.099.
5. Da, C.; Stashko, M.; Jayakody, C.; Wang, X.; Janzen, W.; Frye, S.; Kireev, D. Discovery of Mer Kinase Inhibitors by Virtual Screening Using Structural Protein–Ligand Interaction Fingerprints. *Bioorg. Med. Chem.* **2015**, *23*, 1096–1101, doi:10.1016/j.bmc.2015.01.001.
6. Cai, S.X.; Tian, Y.E.; Wang, X.; Zhang, L. Substituted Tricyclic Compounds as PARP Inhibitors and the Use Thereof 2023.
7. Salvati, E.; Botta, L.; Amato, J.; Di Leva, F.S.; Zizza, P.; Gioiello, A.; Pagano, B.; Graziani, G.; Tarsounas, M.; Randazzo, A.; et al. Lead Discovery of Dual G-Quadruplex Stabilizers and Poly(ADP-Ribose) Polymerases (PARPs) Inhibitors: A New Avenue in Anticancer Treatment. *J. Med. Chem.* **2017**, *60*, 3626–3635, doi:10.1021/acs.jmedchem.6b01563.
